# Synthesis, biological evaluation, and molecular modeling studies of new benzoxazole derivatives as PARP-2 inhibitors targeting breast cancer

**DOI:** 10.1038/s41598-022-20260-1

**Published:** 2022-09-28

**Authors:** Nadeen M. El-Ghobashy, Selwan M. El-Sayed, Ihsan A. Shehata, Mahmoud B. El-Ashmawy

**Affiliations:** Department of Medicinal Chemistry Pharmacy, Faculty of Pharmacy, Mansoura University, Mansoura, 35516 Egypt

**Keywords:** Biochemistry, Drug discovery, Chemistry

## Abstract

Many benzoxazole-based and similar scaffolds were reported to have wide-range of anticancer activities. In this study, four series of benzoxazole derivatives were designed by combining benzoxazole scaffold with different amines via a reversed phenyl amide linker to produce the compounds of series **A**, **B** and **C**. A fourth new hybrid of benzoxazole with 1,2,3 triazole ring (series **D**) was also designed. The designed compounds were synthesized and screened for their anti-breast cancer activity against MDA-MB-231 and MCF-7 cell lines using MTT assay. The most potent cytotoxic compounds; **11–14, 21, 22, 25–27** were further evaluated for their in vitro PARP-2 enzyme inhibition. Compounds **12** and **27** proved to be the most active PARP-2 inhibitors with IC_50_ values of 0.07 and 0.057 µM, respectively. Compounds **12** and **27** caused cell cycle arrest in mutant MCF-7 cell line at G2/M and G1/S phase, respectively and they possessed significant apoptosis-promoting activity. Docking results of compounds **12** and **27** into PARP-2 pocket demonstrated binding interactions comparable to those of olaparib. Their predicted pharmacokinetic parameters and oral bioavailability appeared to be appropriate. Collectively, it could be concluded that compounds **12** and **27** are promising anti-breast cancer agents that act as PARP-2 inhibitors with potent apoptotic activity.

## Introduction

Breast cancer is one of the most prevalent malignancies in women worldwide. According to the 2020 World Health Organization (WHO) statistics, this type of cancer affected approximately 2.3 million women, causing 685,000 fatalities globally^1^. In Egypt, breast cancer represents the most widespread malignant tumor between women, constituting about 38.8% of all cancers. In 2020, there were approximately 22,700 Egyptian women diagnosed with breast cancer. It represented the second most common cause of cancer-related deaths, after liver cancer, with a mortality rate of around 11%^2^. Because breast cancer is a heterogeneous disease showing differentiation in phenotypes and morphological features and leading to various clinical conducts; various options are used for treatment including surgical interventions, radiotherapy, chemotherapy and hormonal therapy^3,4^.

**Figure 1:**
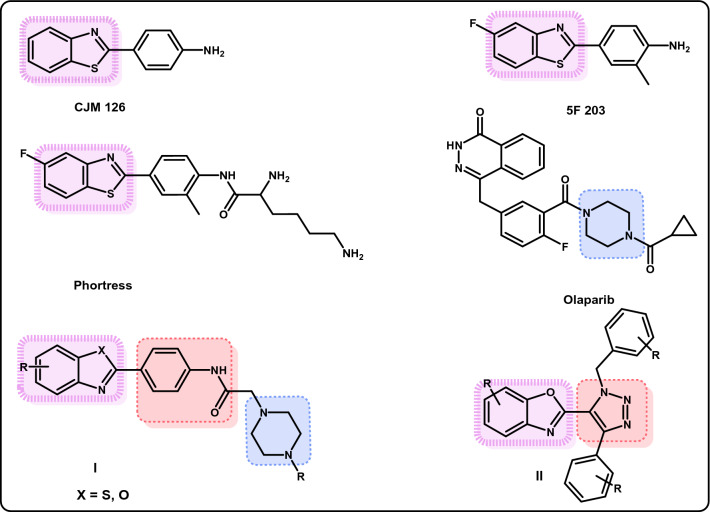
Chemical structures of some reported anticancer agents.

Breast cancer treatment with a group of drugs called Poly (ADP-ribose) polymerase (PARP) inhibitors exhibited promising results; they can be used alone or in combination with other chemotherapeutic agents or radiotherapy^4^. PARP family is a group of proteins involved in many critical cellular processes including DNA single-strand break (SSB) repair and cell death control^5,6^. Treatment with PARP inhibitors is a therapeutic strategy that relies on the inhibition of DNA damage repair by base excision, which is a pivotal process for cells, especially those with homologous recombination repair defects caused by BRCA1 and BRCA2 mutations^6,7^. These two gene mutations (BRCA1 and BRCA2) were reported to be the main cause for triple negative breast cancer development^8^. Since PARP inhibitors were found to inhibit the repair of DNA damage caused by these two mutations, inhibition of PARP was considered as a successful strategy for breast cancer treatment^8^. Design and development of many PARP inhibitors were performed, and some were found clinically useful. Olaparib (Fig. 1), which is a PARP-1 and PARP-2 inhibitor, was approved by the US Food and Drug Administration (FDA) to treat certain types of BRCA1/2 mutated breast cancer^8^. Herein, new prototypes of PARP-2 inhibitors were designed aiming to develop antitumor therapeutics to treat breast cancer.

Benzothiazoles and their related bioisosteres, benzoxazoles, represent useful scaffolds with wide-range of anticancer activities against different tumors^9–12^. About 25 years ago, a simple 2-(4-aminophenyl) benzothiazole (CJM 126) (Fig. 1) was reported as an original lead compound to design and synthesize new molecules with anticancer activity towards human breast cancer cell lines^13–15^. A fluorinated analogue, namely: 2-(4-amino-3-methylphenyl)-5-fluoro-benzothiazole (5F 203) (Fig. 1) showed remarkable antitumor activity against National Cancer Institute (NCI) 60 cell panel^16^. Its lysylamide prodrug was then prepared to improve lipophilicity and water solubility and named Phortress^16^ (Fig. 1). Herein, the benzothiazole moiety of phortress was isosterically replaced by a benzoxazole one. However, we extended the molecule with other parts, that ended in the structural comparison with olaparib, the PARP inhibitor. In addition, benzothiazole and benzoxazole derivatives **I** containing a phenyl reversed amide linker were reported to have potent anticancer activity against MCF-7 and MDA-MB-231 breast cancer cell lines^12,17^. The presence of the 5-chloro substituent on the benzoxazole ring of **I** appeared important to contribute positively to the overall anticancer activity of the compounds^17^. Moreover, literature survey revealed that compounds **II** containing both a benzoxazole scaffold and 1,2,3-triazole functionality exhibited potent anticancer activity (Fig. 1)^18^.

**Figure 2:**
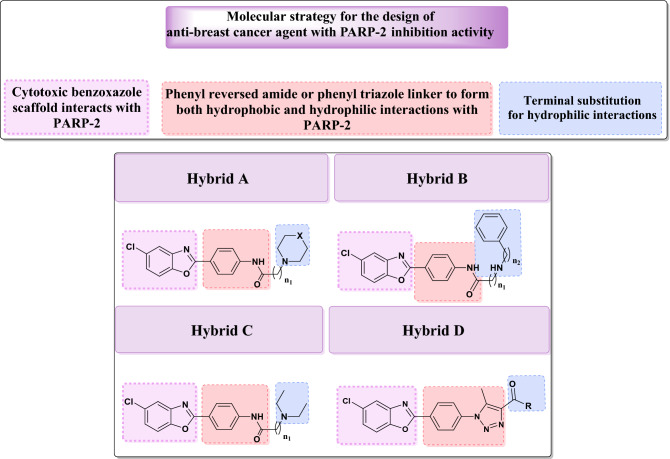
Molecular strategy for the design of new benzoxazole hybrids as anticancer agents.

Molecular hybridization is an effective approach in drug discovery that has been applied to combine two or more pharmacophoric moieties of previously reported active compounds to obtain new hybrid compounds that might improve affinity and efficacy^19^. This strategy is used, herein, to design the new anti-breast cancer agents with PARP-2 inhibitory activity by combining the benzoxazole scaffold^10^ with a terminal amine via a phenyl reversed amide linker as in hybrids **A**, **B** and **C**, or to combine the benzoxazole moiety with 1,2,3 triazole ring via a phenyl linker as in hybrid **D** (Fig. 2). The phenyl reversed amide acts not only as a linker, but also as possible source of both hydrophobic and hydrophilic interactions with PARP-2 enzyme. The 5-chloro substituent on the benzoxazole ring was retained in the newly designed hybrids, based on its previous role in the anticancer activity of compounds **I**^17^.

Thus, new benzoxazole hybrids **A**, **B**, **C** and **D** (Fig. 2) were synthesized and evaluated for in vitro cytotoxicity against MDA-MB-231 and MCF-7 breast cancer cell lines. These two cell lines were chosen based on their solid connection in the previously reported activity of other benzoxazole derivatives (**I**)^12,17^. To explore their action, the most potent compounds were assessed for their PARP-2 enzyme inhibition potency and their cell cycle and apoptosis effects. In addition, some in silico studies, including Lipinski's rule of five compliances, ADMET and molecular modeling studies were included.

## Results and discussion

### Chemistry

**Figure 3:**
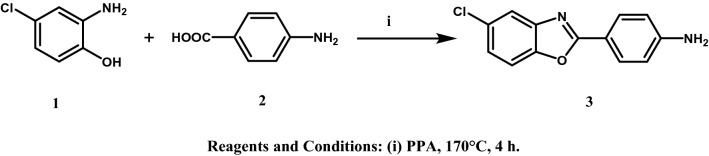
Synthetic scheme of compound **3**.

**Figure 4:**
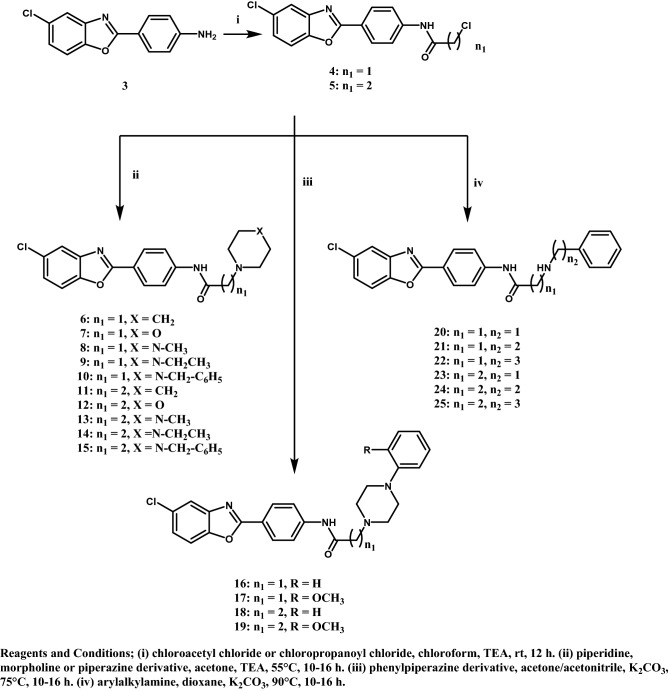
Synthetic scheme of target benzoxazole derivatives **6–25.**

**Figure 5:**
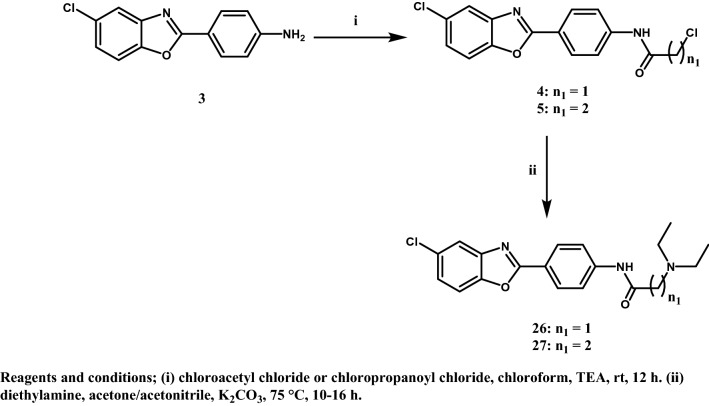
Synthetic scheme of target benzoxazole derivatives **26, 27**.

**Figure 6:**
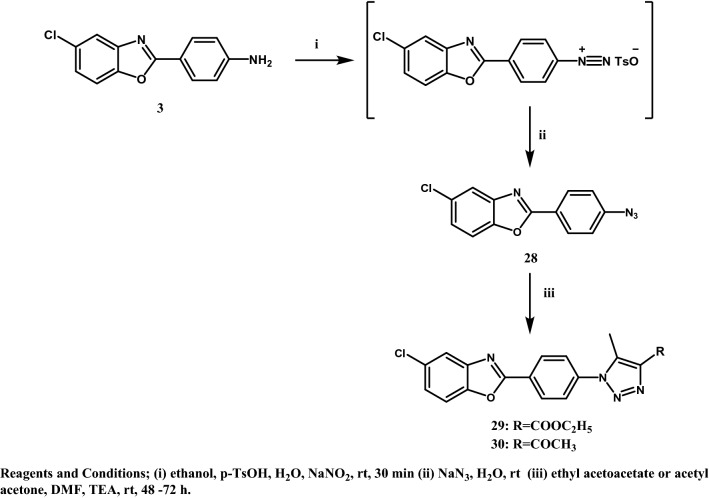
Synthetic scheme of target benzoxazole derivatives **28–30**.

All the designed compounds were synthesized according to the methods depicted in Figs. 3, 4, 5 and 6. The starting material 4-(5-chlorobenzoxazol-2-yl)aniline (**3)**^20^ was prepared by reacting 2-amino-4-chlorophenol (**1)** and 4-aminobenzoic acid (**2)** in polyphosphoric acid (PPA) via a ring closure reaction (Fig. 3) according to reported reaction conditions^17,21^. The amine function of **3** was acylated with 2-chloroacetyl chloride and 3-chloropropanoyl chloride in chloroform using triethylamine (TEA) as a basic catalyst to give the corresponding amides **4** and **5**, respectively. Structures were confirmed by ^1^H NMR through the appearance of aliphatic protons at δ 4.34 ppm for compound **4** and at δ 2.91 and 3.92 ppm for compound **5,** in addition to an amide exchangeable proton at δ 10.74 ppm for compound **4** and 10.49 ppm for compound **5**. The next step consisted of refluxing **4** or **5** with the appropriate secondary or primary amine in the presence of triethylamine (TEA) or anhydrous potassium carbonate (K_2_CO_3_), to yield compounds **6**–**27** (Figs. 4 and 5). The structures of compounds **6–15** were confirmed by ^1^H NMR through the appearance of extra aliphatic peaks at range δ 1.16—3.88 ppm ensuring the addition of the cyclic secondary amines. The structures of compounds **16–19** were confirmed by ^1^H NMR through the appearance of extra aliphatic peaks at range from δ 2.82 to 3.39 ppm that ensured presence of piperazine; in addition to the characteristic methoxy protons peaks for compounds **17** and **19** that appeared at δ 3.91 and 3.92 ppm, respectively. The addition of the primary arylalkylamines in compounds **20–25** were confirmed by ^1^H NMR through the appearance of extra aliphatic protons at range from δ 1.87 to 3.93 ppm. The structures of compounds **26** and **27** were confirmed by ^1^H NMR through the appearance of extra aliphatic protons at range from δ 0.98 to 2.69 ppm. In Fig. 6, the aryl azide intermediate **28** was prepared via a diazotization reaction of the aryl amine **3** with sodium nitrite under acidic conditions followed by the addition of sodium azide. The structure of compound **28** was confirmed by ^1^H NMR through the disappearance of the NH_2_ protons in **3** in addition to the appearance of infrared (IR) absorption band at 2128 cm^-1^ representing formation of the azide group. Finally, ethyl acetoacetate and acetyl acetone were added to the aryl azide in dimethylformamide (DMF) using TEA as catalyst to afford the corresponding triazoles **29** and **30**, respectively. The structures of compounds **29** and **30** were confirmed by ^1^H NMR through the appearance of aliphatic peaks at range from δ 1.49 to 4.51 ppm. Beside ^1^H NMR, the structures of the synthesised compounds were confirmed by ^13^C NMR and mass spectrometry (MS). Some IR were also included.

### Biological evaluation

In vitro cytotoxicity screening.

All the synthesized compounds **3–30** were evaluated for in vitro antitumor activity via standard MTT assay^22,23^ using two human breast cancer cell lines, namely; MDA-MB-231 and MCF-7. Sorafenib was used as a standard anticancer drug. The cytotoxicity results were expressed as IC_50_ which is the concentration that causes 50% cell viability inhibition (Table 1). The tested compounds showed different anticancer potencies. Regarding MDA-MB-231 cell line, compounds **11** and **12** displayed remarkable cytotoxic activities with IC_50_ values of 5.63 and 6.14 µM, respectively. These two compounds were proved to be more potent than the standard anticancer drug sorafenib (IC_50_ = 7.47 µM). Compound **13** showed very potent cytotoxic activity with IC_50_ value of 7.52 µM, while compounds **14, 18, 21, 22, 25, 26** and **27** showed moderate cytotoxic activity with IC_50_ values of 10.78, 19.21, 12.18, 17.37, 15.56, 18.57 and 11.32 µM, respectively. The other compounds were found to have weak or no cytotoxic activity. Concerning MCF-7 cell line, compounds **11** and **12** showed remarkable cytotoxic activities with IC_50_ values of 3.79 and 6.05 µM, respectively. Again, these two compounds were proved to be more potent than the standard anticancer drug sorafenib (IC_50_ value of 7.26 µM). Compound **13** showed very strong cytotoxic activity with IC_50_ value of 8.38 µM, while compounds **3, 14, 21, 25** and **27** showed moderate cytotoxic activity with IC_50_ values of 13.54, 12.47, 16.87, 18.46 and 16.70 µM, respectively. Other compounds were found to have weak or no cytotoxic activity.

**Table 1:** IC_50_ values of compounds **3–30** against MDA-MB-231 and MCF-7 cell lines in (µM).


Compd. No	R	n_1_	n_2_	X	In vitro Cytotoxicity (IC_50_, µM)	
					MDA-MB-231	MCF-7
3	–	–	–	NH_2_	23.98 ± 1.8	13.54 ± 1.1
4	–	1	–	–	43.21 ± 2.7	46.68 ± 3.0
5	–	2	–	–	55.69 ± 3.2	72.84 ± 3.7
6	–	1	–	CH_2_	42.50 ± 2.8	50.35 ± 3.0
7	–	1	–	O	51.63 ± 3.0	61.81 ± 3.5
8	–	1	–	N–CH_3_	30.91 ± 2.3	38.21 ± 2.7
9	–	1	–	N–CH_2_–CH_3_	39.45 ± 2.6	45.93 ± 2.8
10	–	1	–	N–CH_2_–C_6_H_5_	71.74 ± 3.5	86.42 ± 4.2
11	–	2	–	CH_2_	5.63 ± 0.3	3.79 ± 0.2
12	–	2	–	O	6.14 ± 0.5	6.05 ± 0.4
13	–	2	–	N–CH_3_	7.52 ± 0.6	8.38 ± 0.6
14	–	2	–	N–CH_2_–CH_3_	10.78 ± 0.9	12.47 ± 0.9
15	–	2	–	N–CH_2_–C_6_H_5_	35.29 ± 2.5	43.72 ± 2.8
16	H	1	–	–	78.83 ± 3.8	> 100
17	OCH_3_	1	–	–	48.02 ± 2.9	55.80 ± 3.3
18	H	2	–	–	19.21 ± 1.6	29.16 ± 2.3
19	OCH_3_	2	–	–	22.48 ± 1.9	32.83 ± 2.4
20	–	1	1	–	47.59 ± 2.8	53.91 ± 3.2
21	–	1	2	–	12.18 ± 1.0	16.87 ± 1.3
22	–	1	3	–	17.37 ± 1.4	27.44 ± 2.1
23	–	2	1	–	62.13 ± 3.4	81.37 ± 4.0
24	–	2	2	–	26.54 ± 2.1	34.52 ± 2.5
25	–	2	3	–	15.56 ± 1.2	18.46 ± 1.5
26	–	1	–	–	18.57 ± 1.3	35.03 ± 2.5
27	–	2	–	–	11.32 ± 0.9	16.70 ± 1.4
28	–	–	–	N_3_	< 100	> 100
29	COOC_2_H_5_	–	–	–	76.01 ± 3.8	> 100
30	COCH_3_	–	–	–	73.87 ± 3.6	93.53 ± 4.9
Sorafenib	–	–	–	–	7.47 ± 0.3	7.26 ± 0.3

Significant values are in bold.

#### Poly (ADP-ribose) polymerase 2 (PARP-2) inhibition assay

The most promising compounds **11–14, 21, 22** and **25–27** were subjected to in vitro Poly (ADP-ribose) polymerase 2 (PARP-2) inhibition assay as an attempt to investigate their possible mechanism of cytotoxicity. Results were expressed as IC_50_ values (Table 2). Olaparib was used as reference drug (IC_50_ value of 0.02 µM). Compounds **12, 14, 25** and **27** displayed the highest (PARP-2) inhibitory activity with IC_50_ values of 0.07, 0.084, 0.074 and 0.057 µM, respectively. Compounds **11** and **13** appeared with slightly less inhibitory activity against PARP-2, with IC_50_ values of 0.19 and 0.106 µM respectively. The slight chemical change between compounds **11** and **12** could explain such difference, due to the presence of oxygen atom in **12**. Compounds **22** and **26** showed intermediate (PARP-2) inhibitory activity with IC_50_ values 0.267 and 0.292 µM, respectively. Compound **21** proved to be the least potent (PARP-2) inhibitory activity with IC_50_ value of 0.406 µM.

**Table 2:** Poly (ADP-ribose) polymerase 2 (PARP-2) inhibition results (IC_50_ µM) of compounds **11–14**, **21, 22** and **25–27** against olaparib.


Compd. No	X	n_1_	n_2_	IC_50_ (µM)
11	CH_2_	2	–	0.190 ± 0.010
12	O	2	–	0.070 ± 0.004
13	N–CH_3_	2	–	0.106 ± 0.006
14	N–CH_2_–CH_3_	2	–	0.084 ± 0.005
21	–	1	2	0.406 ± 0.022
22	–	1	3	0.267 ± 0.015
25	–	2	3	0.074 ± 0.004
26	–	1	–	0.292 ± 0.016
27	–	2	–	0.057 ± 0.003
Olaparib	–	–	–	0.020 ± 0.002

#### Cell cycle analysis

**Figure 7:**
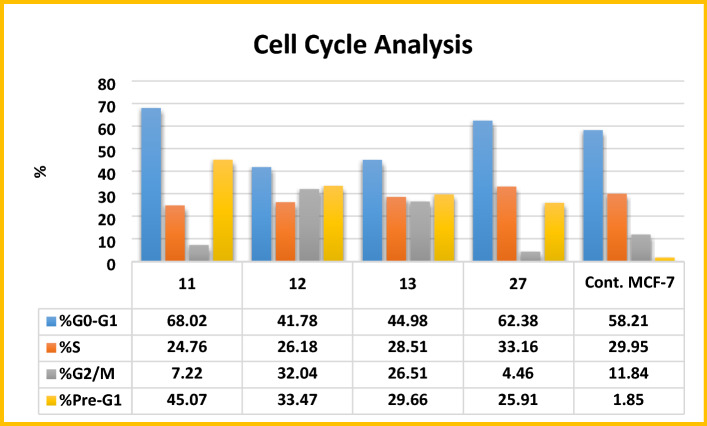
Cell cycle phase distribution in MCF-7 cell line treated with vehicle control and the newly synthesised compounds; **11**, **12**, **13** and **27**.

**Figure 8:**
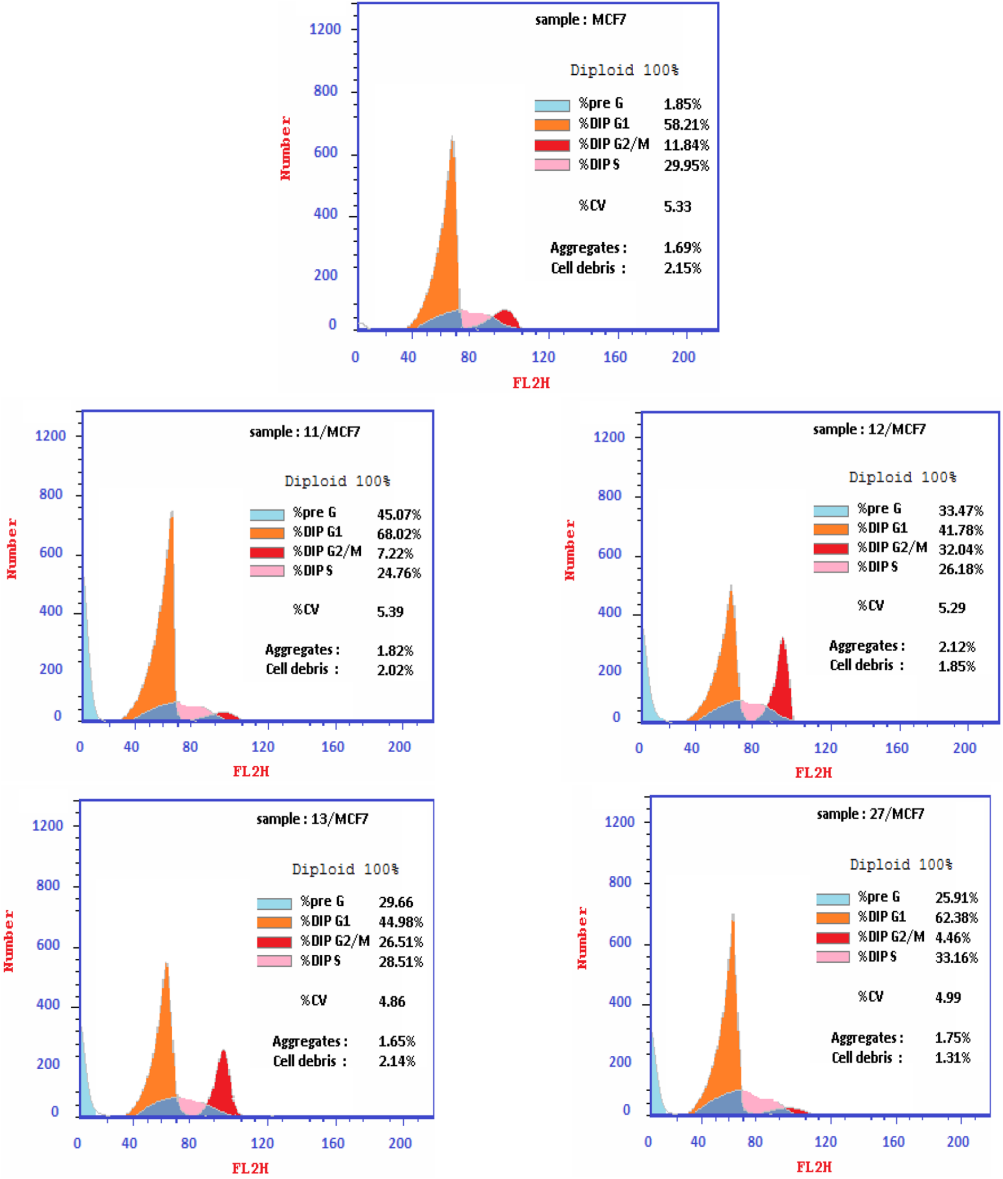
Flow cytometry analysis of cell cycle phase distribution in MCF-7 cells after treatment with vehicle control and the newly synthesised compounds; **11, 12, 13** and **27**.

For further investigation of the promising antiproliferative activity of compounds **11, 12, 13** and **27** on breast cancer MCF-7 cell line, cell cycle analysis was carried out using flow cytometric assay. MCF-7 cells were treated with **11, 12, 13** and **27** at their IC_50_ concentrations (3.79 ± 0.2, 6.05 ± 0.4, 8.38 ± 0.6 and 16.70 ± 1.4 µM), respectively. The flow cytometric assay results were compared to the negative control untreated MCF-7 cell line. For **11**, the percentage of cells in pre-G1 phase increased from 1.85% to 45.07%, while in G2/M phase decreased from 11.84 to 7.22%. Percentage of cells in S phase decreased from 29.95 to 24.76% and in G0/G1 phase increased from 58.21 to 68.02%. For **12** and **13,** the percentage of cells in pre-G1 phase increased from 1.85 to 33.47% and 29.66%, respectively, while in G2/M phase increased from 11.84% to 32.04% and 26.51%, respectively. Moreover, the percentage of cells in S phase slightly decreased from 29.95 to 26.18 and 28.51%, respectively and in G0/G1 phase decreased from 58.21 to 41.78 and 44.98%, respectively. For **27** the percentage of cells in pre-G1 phase increased from 1.85 to 25.91%, while in G2/M phase decreased from 11.84 to 4.46%. Moreover, the percentage of cells in S phase increased from 29.95 to 33.16% and in G0/G1 phase increased from 58.21 to 62.38% (Fig. 7 and 8). These results indicated that, compound **11** induced cell cycle arrest at G0/G1 phase, compounds **12** and **13** induced cell cycle arrest at G2/M phase, while compound **27** induced cell cycle arrest at G1/S phase.

#### Detection of apoptosis and necrosis by flow cytometry assay

**Figure 9:**
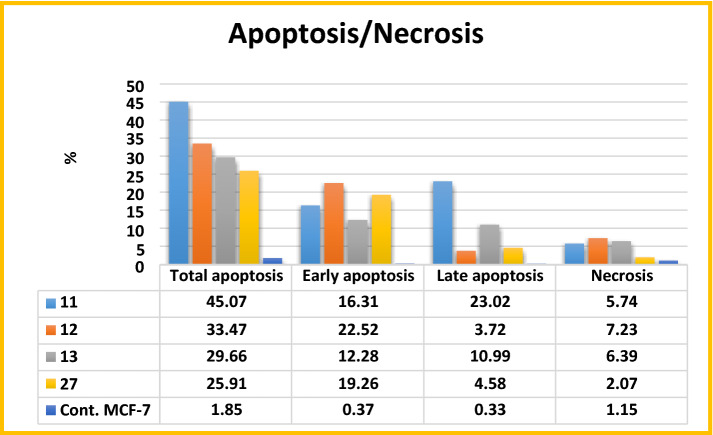
Apoptosis percentage in MCF-7 cells treated with vehicle control and the newly synthesised compounds; **11, 12, 13** and **27.**

**Figure 10:**
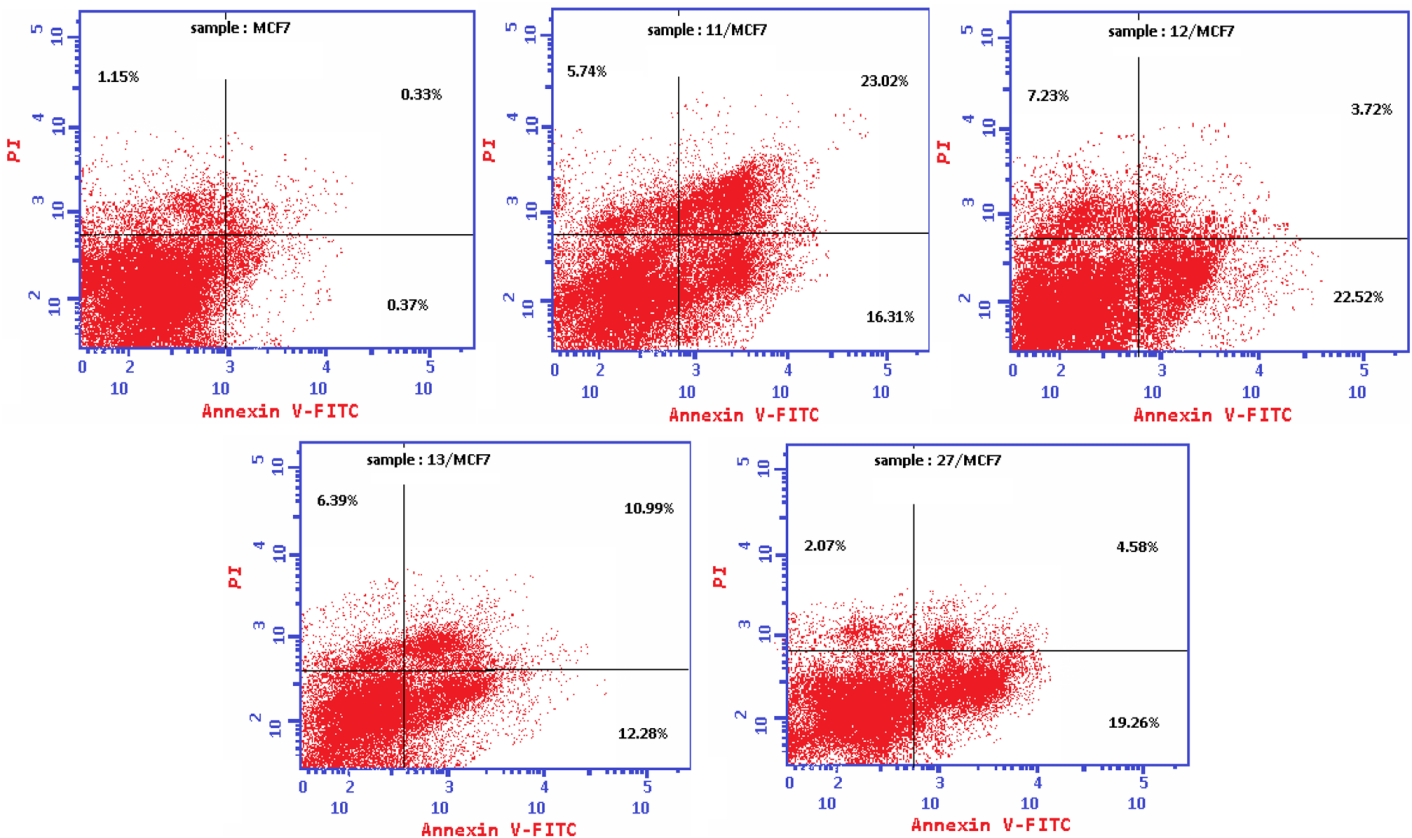
Annexin V-FITC/PI double staining to detect apoptosis in MCF-7 cells after treatment with vehicle control and the newly synthesised compounds; **11, 12, 13** and **27**.

Apoptosis, which is a programmed cell death, is considered as an important way to express cell death among anticancer agents. Most of the anticancer agents can induce apoptosis in cancerous cells. To examine whether the potent cytotoxic effect of **11, 12, 13** and **27** was attributed to apoptosis or necrosis, annexin V-FITC propidium iodide (PI) double staining flow cytometry assay was applied. MCF-7 cells were treated with the test compounds **11, 12, 13** and **27** at their IC_50_ concentrations (3.79 ± 0.2, 6.05 ± 0.4, 8.38 ± 0.6 and 16.70 ± 1.4 µM), respectively and untreated MCF-7 cell line was used as negative control. The cells were then stained with annexin V-FITC propidium iodide (PI) and the percentage of apoptotic cells was determined by flow cytometry. The results revealed that the tested compounds **11, 12, 13** and **27** induced an early apoptotic effect 16.31%, 22.52%, 12.28% and 19.26%, respectively and late apoptotic effect 23.02%, 3.72%, 10.99% and 4.58%, respectively in comparison to the untreated negative control MCF-7 cells which induced an early and late apoptotic effect 0.37% and 0.33%, respectively (Figs. 9 and 10). Compounds **11, 12, 13** and **27** induced necrotic effect 5.74%, 7.23%, 6.39% and 2.07%, respectively in comparison to the untreated negative control MCF-7 cells (1.15%). These results proved that compounds **11, 12, 13** and **27** can induce potent apoptotic and weak necrotic effect in MCF-7 breast cancer cell line.

### Molecular modeling study

Molecular modeling techniques^24,25^ were used to get an enriched insight about the binding modes, interactions and affinities of a molecule to a target protein receptor. They also can be used to investigate the surface properties of a biological system. In this investigation, molecular modeling was performed using MOE 2009.10 software^26^ for better understanding the behavior of the most potent PARP-2 inhibitors in comparison to olaparib.

#### Molecular docking into the catalytic domain of PARP-2 enzyme

**Figure 11:**
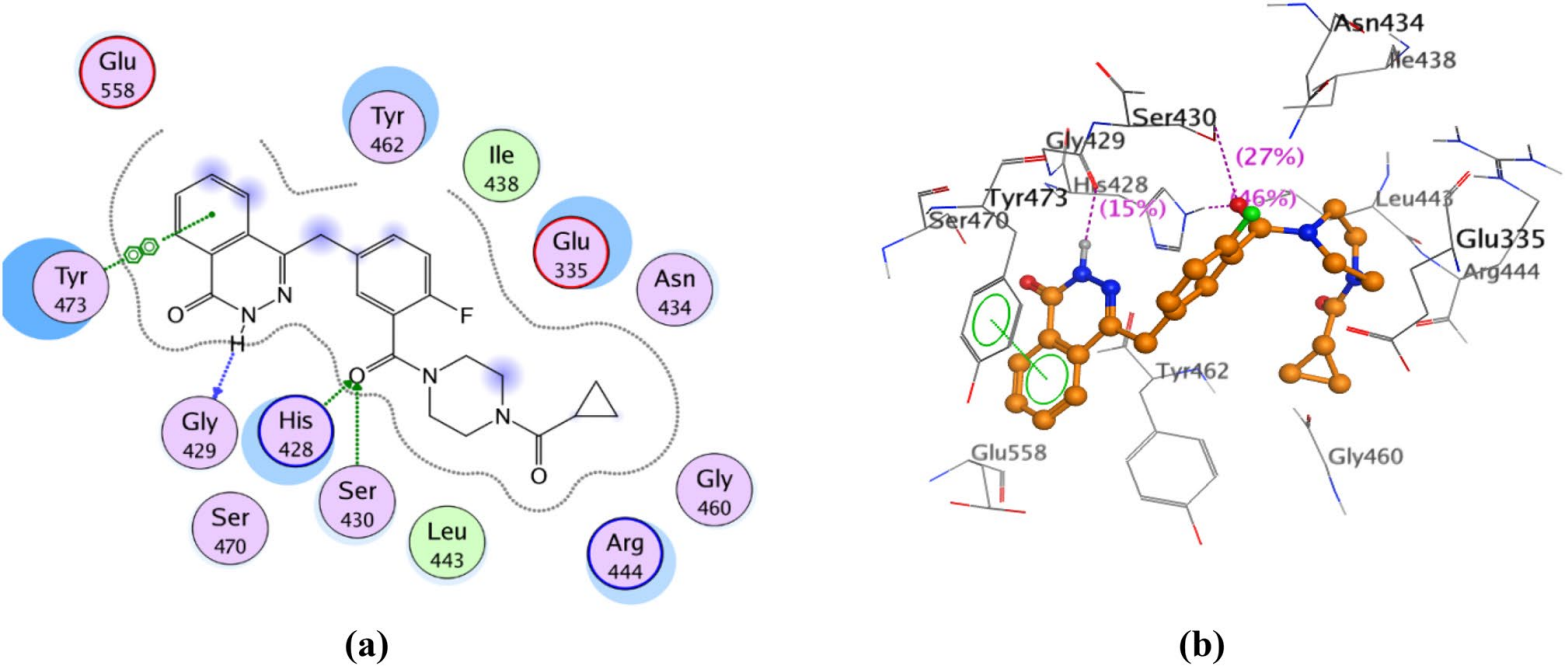
(**a**) 2D view of the interactions between olaparib and PARP-2 enzyme active site. (**b**) 3D view of the interactions between olaparib and PARP-2 enzyme active site.

**Figure 12:**
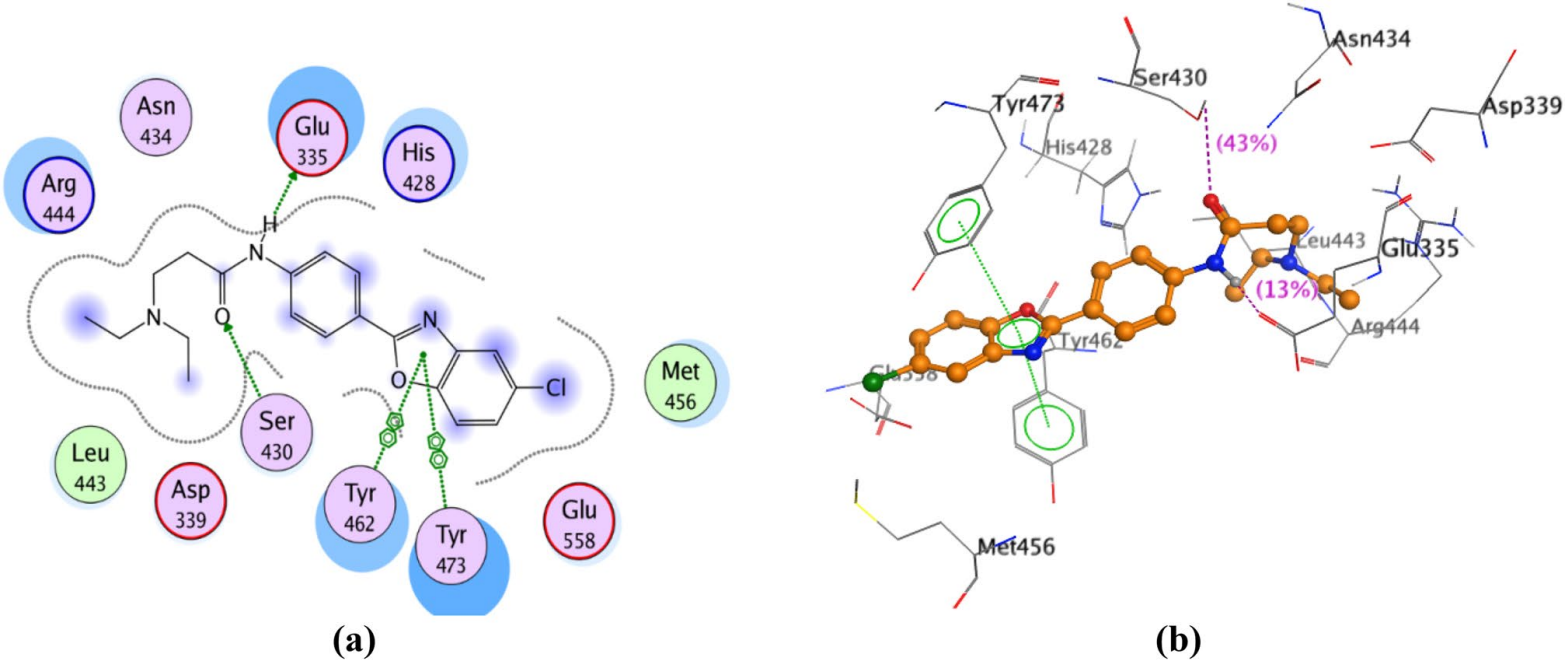
(**a**) 2D view of the interactions between **27** and PARP-2 enzyme active site. (**b**) 3D view of the interactions between **27** and PARP-2 enzyme active site.

**Figure 13:**
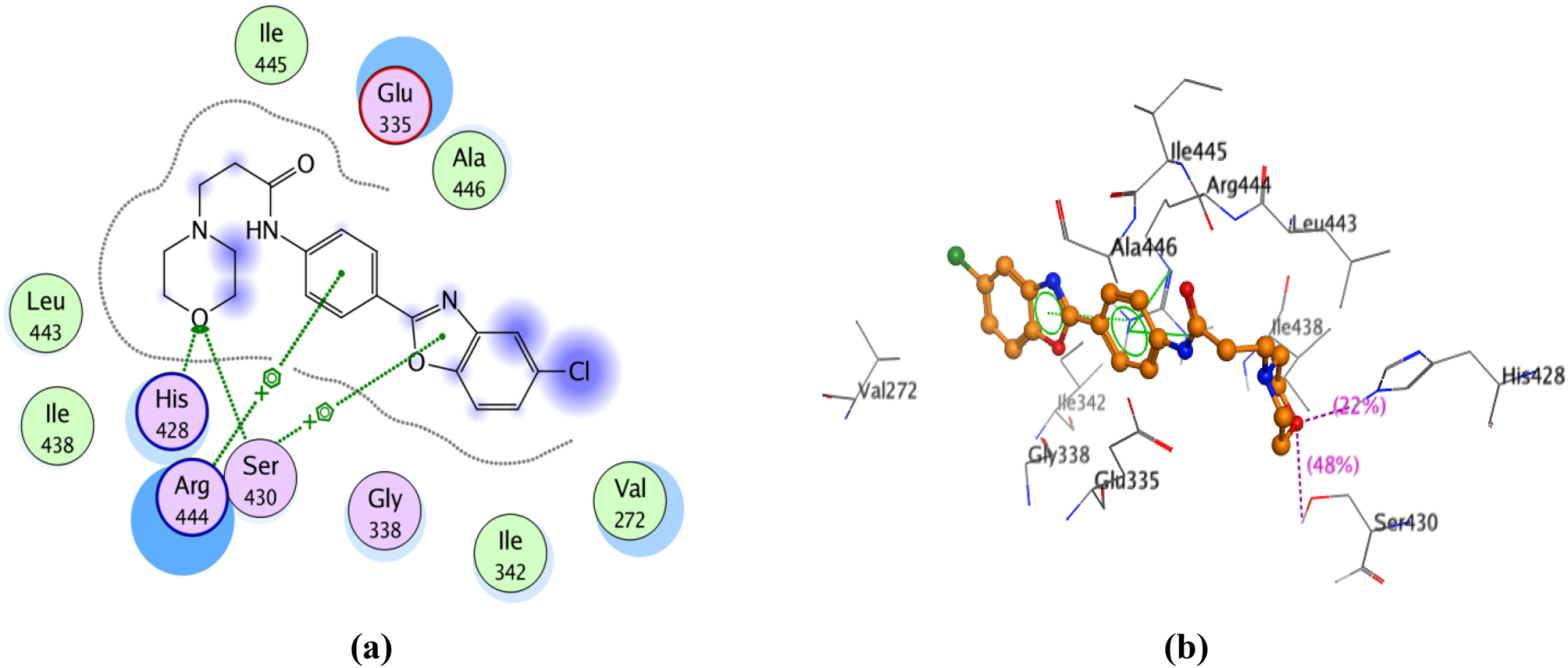
(**a**) 2D view of the interactions between **12** and PARP-2 enzyme active site. (**b**) 3D view of the interactions between **12** and PARP-2 enzyme active site.

Docking studies of the promising compounds **11–14, 21, 22, 25–27** and olaparib were carried out using the crystal structure of human ARTD2 (PARP-2)—catalytic domain in complex with olaparib (PDB Code: 4TVJ)^27^ to predict their binding affinity as PARP-2 inhibitors. Such crystal structure was chosen, based on its co-crystallization with olaparib; and we intend to use the same active site occupied by olaparib for the docking process. Besides, olaparib is the reference drug used as a standard for the enzyme inhibition assay. Table 3 represents the binding scores of the nine compounds and olaparib with PARP-2 active site. The docking results of olaparib (IC_50_ 0.02 ± 0.002 µM) showed that the carbonyl group, lying between the piperazinyl moiety and the fluorobenzyl moiety, was involved in two hydrogen bonding with Ser 430 and His 428 residues. The hydrogen atom of the hydrazide function present in the phthalazinone moiety formed hydrogen bonding with Gly 429 residue. In addition, arene-arene interaction was observed between the phenyl group in the phthalazinone moiety and Tyr 473 residue. Hydrophobic interactions with Tyr 473, Tyr 462, His 428, Glu 335, Arg 444, Glu 558, Ser 430, Leu 443, Asn 434 and Ile 438 residues were also identified (Fig. 11). The docking results of **27** (IC_50_ 0.057 ± 0.003 µM) showed that it can interact with a large number of the essential amino acids present in the active site of PARP-2 enzyme. Hydrogen bonding between the carbonyl group of the amide function and Ser 430 residue was observed, in addition to another hydrogen bonding between the hydrogen atom of the amide function and Glu 335 residue. Furthermore, the benzoxazolyl moiety formed two arene-arene interactions with Tyr 462 and Tyr 473 residues. Other hydrophobic interactions with Tyr 473, Tyr 462, His 428, Glu 335, Arg 444, Glu 558, Ser 430, Leu 443, Asn 434, Asp 339 and Met 456 residues were also observed (Fig. 12). The docking results of **12** (IC_50_ 0.07 ± 0.004 µM) showed its interaction with a large number of the essential amino acids present in the active site of PARP-2 enzyme. The oxygen of the morpholinyl moiety was involved in two hydrogen bonding with Ser 430 and His 428 residues. Furthermore, there were two arene-cation interactions: the first, formed between the oxazole ring and Ser 430 residue and the second, formed between the central phenyl group and Arg 444 residue. Hydrophobic interactions with His 428, Glu 335, Arg 444, Ser 430, Leu 443, Gly 338, Ile 342, Val 272 and Ala 446 residues were also observed (Fig. 13). The results explain collectively the good fitting of compounds **12** and **27** into PARP-2 enzyme active site.

**Table 3:** Binding scores of compounds **11–14, 21, 22, 25–27** and olaparib docked with PARP-2 enzyme.


Compd. No	X	n_1_	n_2_	Binding Score (Kcal/mol)
11	CH_2_	2	–	−22.824
12	O	2	–	−27.888
13	N–CH_3_	2	–	−22.214
14	N–CH_2_–CH_3_	2	–	−23.054
21	–	1	2	−16.441
22	–	1	3	−22.046
25	–	2	3	−24.995
26	–	1	–	−18.169
27	–	2	–	−25.862
Olaparib	–	–	–	−33.905

#### 3D Ligand-based alignment in PARP-2 pocket

**Figure 14:**
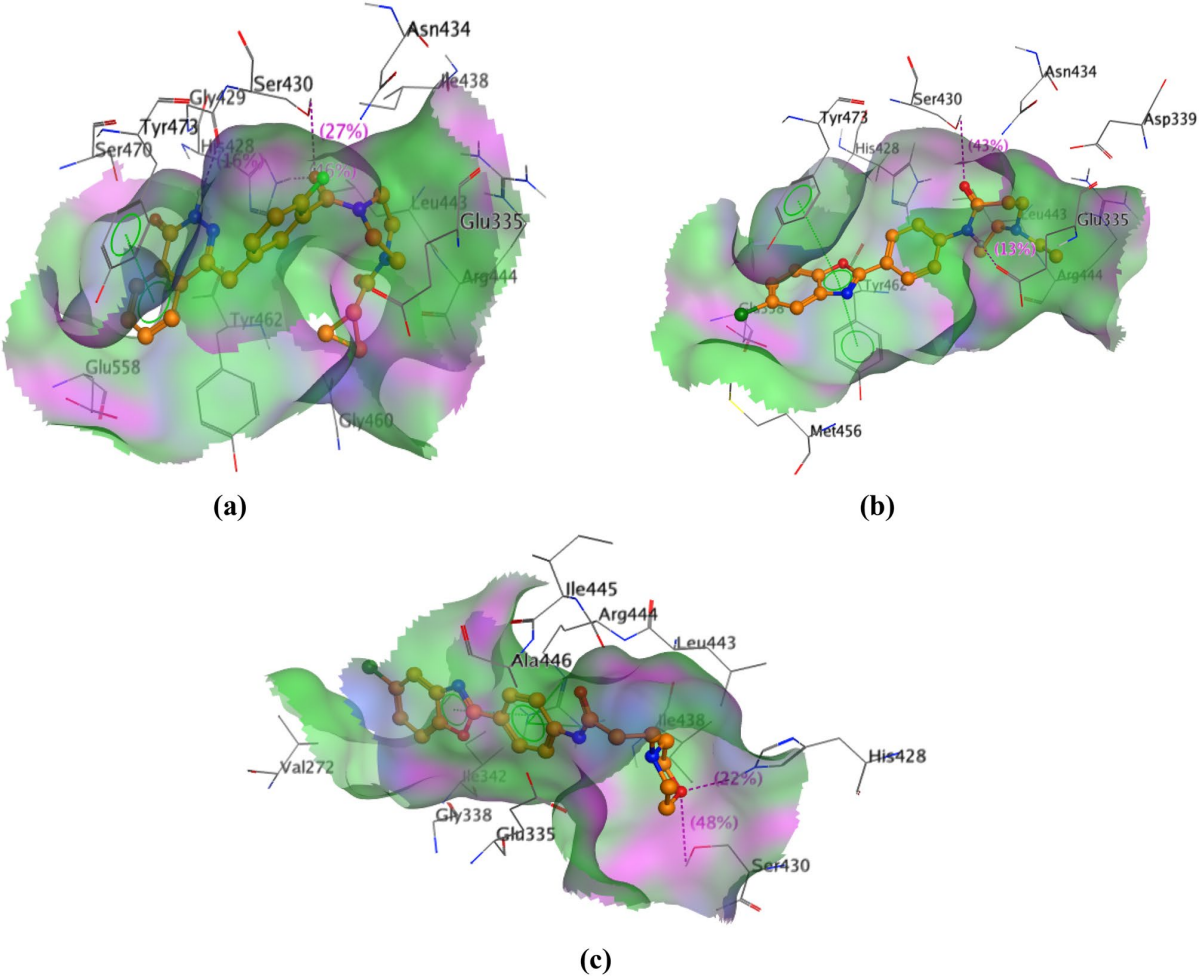
The ligand conformations of (**a**) olaparib, (**b**) **27** and (**c**) **12** inside PARP-2 binding pocket surface map.

Ligands alignment inside PARP-2 binding pocket was performed and the surface map was calculated. It was observed that **12** and **27** filled the space inside the PARP-2 pocket in a manner similar to olaparib (Fig. 14).

#### Flexible alignment

**Figure 15:**
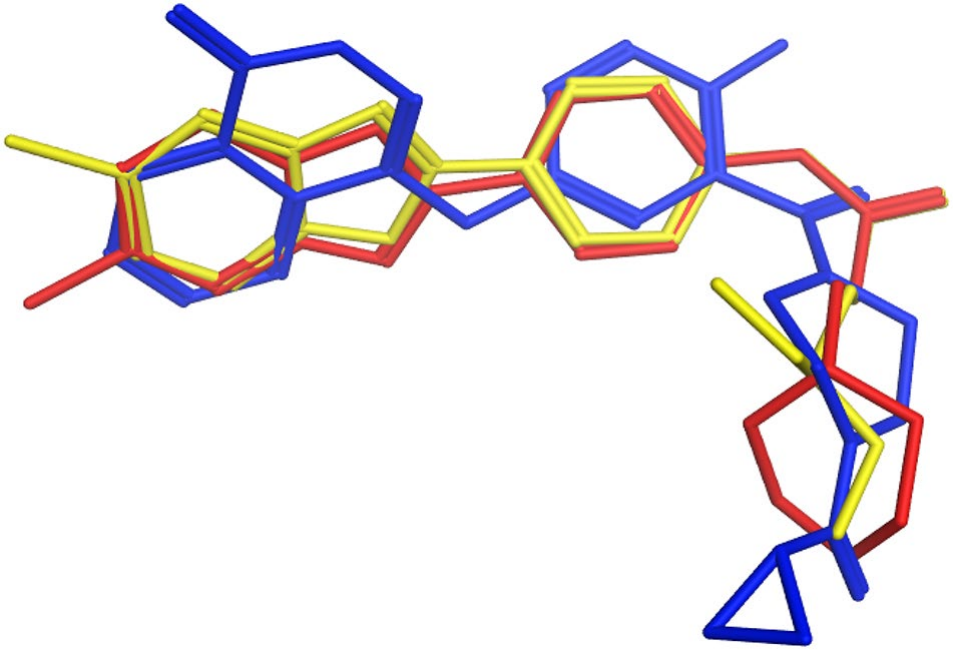
Flexible alignment of the most potent PARP-2 inhibitors **12** (red), **27** (yellow) and olaparib (blue).

Flexible alignment^28^ is a computational procedure which was performed using MMFF94 flexible alignment tool in MOE 2009.10 software to assess the extent of structural similarity between the most potent PARP-2 inhibitors **12, 27** and olaparib. Figure 15 showed that **12** and **27** were perfectly aligned especially at the benzoxazole moiety. They also have great similarity with olaparib as their benzoxazole moiety was aligned with the phthalazinone moiety of olaparib, in addition to the alignment of their phenylpropanamide moiety with benzyl attached to the 1-carbonyl piperazine in olaparib. These structure similarities supported the in vitro enzyme inhibition results of **12** and **27**.

#### ADMET studies

SwissADME website^29,30^ was used to predict the physicochemical properties, ADME parameters and druglikeness of the most potent compounds **11–14, 21, 22** and **25–27**. It was observed that all the tested compounds obey Lipinski’s rule of five without violations (Table 4). Lipinski’s rule of five is important in predicting the oral activity of drugs in humans. Moreover, the number of rotatable bonds is considered an important measure of molecular flexibility; for a compound to possess acceptable oral bioavailability, the number of rotatable bonds should be ≤ 10. Similarly, topological polar surface area (TPSA), which is the surface sum over all the polar atoms, mainly oxygen, nitrogen and their attached hydrogen atoms, has a great effect on drug absorption and oral bioavailability. For a drug to have good oral bioavailability, it should have TPSA value > 140^31^. Since all the tested compounds have TPSA values ranging from 58.37 to 67.60, they should theoretically have promising oral absorption. The prediction results showed that all the tested compounds seem to be possible drug molecules. AdmetSAR website^32,33^ was used to predict pharmacokinetic properties such as oral bioavailability and blood brain barrier (BBB) penetration probabilities. All the selected compounds had acceptable gastrointestinal absorption and hence oral bioavailability. In addition to optimum BBB penetration with acceptable water solubility higher than -0.4 (Table 5).

**Table 4:** Calculated parameters of Lipinski's rule of five, its violation and drug-likeness for compounds **11–14, 21, 22** and **25–27.**

Compd. No	Parameters of Lipinski's rule of five						
	TPSA^a^	Log P^b^	MW^c^	*n*HBA^d^	*n*HBD^e^	*n*RB^f^	*n*Vs^g^
11	58.37	4.03	383.88	4	1	6	0
12	67.60	3.56	385.85	5	1	6	0
13	61.61	3.87	398.89	5	1	6	0
14	61.61	4.12	412.92	5	1	7	0
21	67.16	3.57	405.88	4	2	8	0
22	67.16	3.85	419.91	4	2	9	0
25	67.16	4.21	433.94	4	2	10	0
26	58.37	3.56	357.84	4	1	7	0
27	58.37	3.93	371.87	4	1	8	0

^a^Topological polar surface area, ^b^Calculated lipophilicity, ^c^Molecular weight. ^d^Number of hydrogen bond acceptors, ^e^Number of hydrogen bond donors, ^f^Number of rotatable bonds. ^g^Number of violation to Lipinski's rule of five.

**Table 5:** AdmetSAR prediction of Log S, GI absorption, oral bioavailability and BBB penetration for compounds **11–14, 21, 22** and **25–27.**

Compd. No	Log S^a^	GI^b^ absorption	Oral bioavailability	BBB^c^ penetration
11	−4.175	0.9684	0.5286	0.9959
12	−4.146	0.9703	0.6714	0.9930
13	−3.819	0.9684	0.5571	0.9955
14	−4.086	0.9764	0.5286	0.9942
21	−3.582	0.9458	0.5429	0.9859
22	−3.713	0.9458	0.5429	0.9859
25	−3.851	0.9458	0.5571	0.9848
26	−4.147	0.9806	0.6000	0.9823
27	−4.04	0.9764	0.6286	0.9916

^a^ Solubility parameter.^b^ Gastrointestinal.^c^ Blood brain barrier.

AdmetSAR website^32,33^ was also used to predict carcinogenicity of the most potent compounds **11–14, 21, 22** and **25–27**. Mutagenicity was virtually tested using Ames test^34^. The results revealed that all the tested compounds lack any carcinogenic or mutagenic effect (Table 6).

**Table 6:** AdmetSAR toxicity prediction for compounds **11–14, 21, 22** and **25–27.**

Compd. No	Carcinogenicity	Ames Mutagenicity
11	Noncarcinogenic	Nonmutagenic
12	Noncarcinogenic	Nonmutagenic
13	Noncarcinogenic	Nonmutagenic
14	Noncarcinogenic	Nonmutagenic
21	Noncarcinogenic	Nonmutagenic
22	Noncarcinogenic	Nonmutagenic
25	Noncarcinogenic	Nonmutagenic
26	Noncarcinogenic	Nonmutagenic
27	Noncarcinogenic	Nonmutagenic

## Structure activity relationship

Regarding the in vitro cytotoxicity screening results against two breast cancer cell lines (MDA-MB-231 and MCF-7), replacement of the free amino group in the starting compound **3 (**IC_50_ value of 23.98 and 13.54 µM) with azido group in **28 (**IC_50_ value greater than 100 µM) abolished the anticancer activity. The triazole derivatives **29** and **30** did not show any promising anticancer activity. The chloroacetamide and chloropropanamide derivatives **4 (**IC_50_ value of 43.21 and 46.68 µM) and **5 (**IC_50_ value of 55.69 and 72.84 µM) showed decreased cytotoxic effect, concluding that the type of substitution terminated with chlorine atom is not suitable for the activity. Substitution of the terminal chlorine atom in **4** and **5** with a variety of different cyclic secondary amines namely; piperidine, morpholine, methylpiperazine, ethylpiperazine and benzylpiperazine resulted in a group of compounds with basic tertiary amine moiety, that vary in the cytotoxic activity **6–15**. Regarding the acetamide derivatives **6–10**, the methylpiperazin**e** derivative **8 (**IC_50_ value of 30.91 and 38.21 µM) proved to be the most potent in this series followed by the ethylpiperazine derivative **9,** while the piperidine derivative **6** did not show marked increase in the activity. On the other hand, the morpholine derivative **7** and benzylpiperazine derivative **10** showed decreased cytotoxic activity. Regarding the propanamide derivatives **11–15**, the five derivatives were found to be more potent than the chloropropanamide derivative **5**. The piperidine derivative **11 (**IC_50_ value of 5.63 and 3.79 µM) was found to be the most potent in this series being slightly more potent than the morpholine derivative **12 (**IC_50_ value of 6.14 and 6.05 µM), followed by the methylpiperazine derivative **13** which was found to be more potent than the ethylpiperazine derivative **14**, while the benzylpiperazine derivative was found to be the least potent in this series. From the previous results, we conclude that bigger substitution is not tolerated. Most important is the distance effect: propanamide derivatives with **a** distance of three atoms between the tertiary amine and the amide NH performed much better than their acetamide homologs.

Substitution of the terminal chlorine atom in **4** and **5** with secondary aliphatic diethylamine resulted in **26** and **27** with marked increase in the anticancer potency. Again, the propanamide derivative **27 (**IC_50_ value of 11.32 and 16.70 µM) performed better than the acetamide **26 (**IC_50_ value of 18.57 and 35.03 µM), to conform the importance of three-atom distance. Compared to the cyclic secondary amines piperidine **11 (**IC_50_ value of 5.63 and 3.79 µM) and morpholine **12 (**IC_50_ value of 6.14 and 6.05 µM) derivatives, compound **27** showed decreased anticancer potency.

Substitution of the terminal chlorine atom in the chloroacetamide and chloropropanamide derivatives **4** and **5** with phenylpiperazine and 1-(2-methoxyphenyl)piperazine resulted in compounds **16–19** with different cytotoxic activities. Regarding the acetamide derivatives **16** and **17**, they showed decrease in the anticancer potency especially the phenylpiperazine **16**. As for the propanamide derivatives **18** and **19**, the phenylpiperazine derivative **18** showed superior activity to the 1-(2-methoxyphenyl)piperazine **19** derivative. The results confirmed again that three-atom-distance between tertiary amine and amide NH had better results than the 2 atoms distance.

Substitution of the terminal chlorine atom in **4** and **5** with a series of primary arylalkylamines namely, benzylamine, 2-phenylethylamine and 3-phenylpropylamine resulted in a group of compounds with basic secondary amine moiety having different cytotoxic activities **20–25**. As regards the acetamide derivatives **20–22**, the 2-phenylethylamine derivative **21 (**IC_50_ value of 12.18 and 16.87 µM) proved to be the most potent in this series followed by the 3-phenylpropylamine derivative **22 (**IC_50_ value of 17.37 and 27.44 µM). Meanwhile, the benzylamine derivative **20** showed decreased anticancer potency. Regarding the propanamide derivatives **23–25**, the 3-phenylpropylamine derivative **25 (**IC_50_ value of 15.56 and 18.46 µM) proved to be the most potent in this series followed by the 2-phenylethylamine derivative **24**, with anticancer potency superior to the chloropropanamide derivative **5**, while the benzylamine derivative **23** showed decreased anticancer potency.

## Conclusion

Novel benzoxazole derivatives were designed, synthesized, and screened for their anti-breast cancer activity against MDA-MB-231 and MCF-7 cell lines using MTT assay. Compounds **11–14, 21, 22, 25–27** exhibited the highest cytotoxic activity against the tested cell lines and were further evaluated for in vitro PARP-2 enzyme inhibition. Compounds **12** and **27** proved to be the most active PARP-2 inhibitors with IC_50_ values of 0.07 and 0.057 µM, respectively; followed by compounds **11**, **13**, **22** and **26**. The partial consistency of MTT assay and enzyme inhibition results emphasize the likelihood of incorporation of other biological targets. Furthermore, MCF-7 cell lines treated with compounds **12** and **27** exhibited cell cycle arrest at G2/M phase and G1/S phase, respectively, and it was proved that these compounds possess significant apoptosis-promoting activity. Docking results of compounds **12** and **27** into PARP-2 pocket demonstrated comparable binding interactions to olaparib. Consequently, it could be concluded that compounds **12** and **27** are promising anti-breast cancer agents with significant apoptotic activity and appropriate predicted pharmacokinetic parameters and oral bioavailability.

## Experimental work

Starting materials, regents and solvents were purchased from agents of Sigma-Aldrich Co., U.S.A., SD Fine chemicals Pvt. Ltd. India, Fisher Scientific CO., UK and Piochem Co., Egypt. The reactions were monitored by TLC plates (silica gel, 60F245 E, Merck) using (hexane/ethyl acetate) as eluting system and the spots were visualized using Ultraviolet light, UV lamp (366–245 nm). Melting points (°C) were measured using Stuart melting point apparatus (SMP30) and are uncorrected. The IR spectra (KBr disc) were performed in central laboratory unit, Faculty of pharmacy, Mansoura University on Mattson 5000 FT IR spectrophotometer (υ in cm^-1^). ^1^H NMR and ^13^C NMR spectra were recorded on Bruker Avance III HD FT (400 MHz) at Faculty of pharmacy, Mansoura University; all chemical shifts are expressed in ppm with reference to tetramethylsilane (TMS). Mass spectral analyses were carried out on Direct Inlet part to mass analyzer in Thermo Scientific GCMS model ISQ at the Regional Center for Mycology and Biotechnology (RCMB), Al-Azhar University, Nasr City, Cairo. In vitro cytotoxicity assay was performed in the Department of Pharmacognosy, Faculty of Pharmacy, Mansoura University. Poly [ADP-ribose] polymerase 2 (PARP-2) in vitro enzyme inhibitory assay and cell cycle analysis were performed at the confirmatory diagnostic unit, VACSERA, Egypt.

### Chemistry

#### Procedure for synthesis of 4-(5-chlorobenzoxazol-2-yl)aniline(3):

A mixture of 2-amino-4-chlorophenol **(1)** (2 g, 14 mmol), 4-aminobenzoic acid **(2)** (1.92 g, 14 mmol) and polyphosphoric acid PPA (17 g) was heated in an oil bath at 170 °C for 4 h. The resulting solution was permitted to cool, diluted with ice-water and neutralized to pH 7 with saturated sodium carbonate solution. The separated solid product was collected by filtration, washed several times with generous amount of water, dried and recrystallized from aqueous ethanol to yield compound **3**^20^.

#### General procedure for synthesis of 2-chloro-N-(4-(5-chlorobenzoxazol-2-yl)phenyl)acetamide (4) and 3-chloro-N-(4-(5-chlorobenzoxazol-2-yl)phenyl)propanamide(5):

To an ice-cooled solution of 4-(5-chlorobenzoxazol-2-yl)aniline **3**, (0.49 g, 2 mmol) and triethylamine (0.33 ml, 2.4 mmol) in chloroform (30 ml), 2-chloroacetyl chloride or 3-chloropropanoyl chloride (2.4 mmol) was added dropwise in a fuming hood. The reaction mixture was stirred at room temperature for 12 h. After completion of the reaction, the solvent was evaporated under pressure and the obtained residue was washed with ice-cooled diluted ammonia solution, dried, washed several times with ice-water and recrystallized from ethanol to yield compounds **4** and **5**.

#### 2-chloro-N-(4-(5-chlorobenzoxazol-2-yl)phenyl)acetamide(4):

Pink solid, yield (76%), m.p. 218–220 ºC. ^1^H NMR (400 MHz, DMSO-d_6_) δ 10.74 (s, 1H, NHCO), 8.18 (d, *J* = 8.4 Hz, 2H, Ar–H), 7.93—7.79 (m, 4H, Ar–H), 7.46 (d, *J* = 8.6 Hz, 1H, Ar–H), 4.34 (s, 2H, –COCH_2_). ^13^ C NMR: δ 165.70, 164.07, 149.46, 143.42, 142.57, 129.46, 129.07, 125.71, 121.42, 119.96, 119.77, 112.65, 44.10. MS m/z (%); 324.15 (M^+^ + 4, 2.06), 321.84 (M^+^ + 2, 8.02), 320.03 (M^+^, 17.87), 63.21 (100.00).

#### 3-chloro-N-(4-(5-chlorobenzoxazol-2-yl)phenyl)propanamide(5):

Violet solid, yield (73%), m.p. 210–212 ºC. ^1^H NMR (400 MHz, DMSO-d_6_) δ 10.49 (s, 1H,NHCO), 8.17 (d, *J* = 8.6 Hz, 2H, Ar–H), 7.92—7.84 (m, 3H, Ar–H), 7.82 (d, *J* = 8.6 Hz, 1H, Ar–H), 7.46 (dd, *J* = 1.7, 8.6 Hz, 1H, Ar–H), 3.92 (t, *J* = 6.1 Hz, 2H, COCH_2_CH_2_), 2.91 (t, *J* = 6.1 Hz, 2H, COCH_2_CH_2_) ^13^ C NMR: δ 169.12, 164.17, 149.44, 143.45, 143.03, 129.43, 129.02, 125.62, 120.91, 119.71, 119.68, 112.60, 41.11, 39.84. MS m/z (%); 338.20 (M^+^ + 4, 1.57), 336.31 (M^+^ + 2, 5.29), 334.16 (M^+^, 9.24), 244.05 (100.00).

#### General procedure for synthesis of compounds(6–15)

A mixture of 2-chloro-N-(4-(5-chlorobenzoxazol-2-yl)phenyl)acetamide **(4)** or 3-chloro-N-(4-(5-chlorobenzoxazol-2-yl)phenyl)propanamide **(5)** (1.5 mmol), triethylamine (0.25 ml , 1.8 mmol) and the appropriate secondary amine (piperidine, morpholine or piperazine derivative) (1.8 mmol) was stirred under reflux in acetone (30 ml) at 55 °C for 10–16 h. After completion of the reaction, the solvent was evaporated under pressure and the obtained residue was washed several times with ice-water, dried and recrystallized from ethanol to yield compounds **6–15**.

#### *N-(4-(5-*chlorobenzoxazol*-2-yl)phenyl)-2-(piperidin-1-yl)acetamide*(6):

Light brown solid, yield (78%), m.p. 180–182 °C. ^1^H NMR (400 MHz, CDCl_3_) δ 10.01 (s, 1H, NHCO), 8.21 (d, *J* = 8.4 Hz, 2H, Ar–H), 7.84 (d, *J* = 8.4 Hz, 2H, Ar–H), 7.73 (d, *J* = 1.4 Hz, 1H, Ar–H), 7.50 (d, *J* = 8.6 Hz, 1H, ArH), 7.32 (dd, *J* = 1.4, 8.6 Hz, 1H, Ar–H), 3.38 (s, 2H, COCH_2_), 2.81 (s, 4H, Piperidine-2CH_2_), 1.80 (s, 4H, Piperidine-2CH_2_), 1.58 (s, 2H, Piperidine-CH_2_ ). ^13^ C NMR: δ 168.10, 164.09, 149.29, 143.33, 141.01, 129.98, 128.84, 125.16, 122.14, 119.74, 119.42, 111.22, 62.26, 54.75, 25.78, 23.23. MS m/z (%); 371.15 (M^+^ + 2 ,27.52), 369.24 (M^+^ ,63.47), 98.26 (100.00).

#### *N-(4-(5-*chlorobenzoxazol*-2-yl)phenyl)-2-morpholinoacetamide*(7):

Grey solid, yield (60%) , m.p. 178–180 °C. ^1^H NMR (400 MHz, CDCl_3_) δ 9.48 (s, 1H, NHCO), 8.21 (d, *J* = 8.7 Hz, 2H, Ar–H), 7.79 (d* J* = 8.7 Hz, 2H, Ar–H), 7.72 (d, *J* = 2.0 Hz, 1H, Ar–H), 7.49 (d, *J* = 8.6 Hz, 1H, Ar–H), 7.32 (dd, *J* = 2.0, 8.6 Hz, 1H, Ar–H), 3.85 (t, *J* = 4.3 Hz, 4H, Morpholine-2CH_2_), 3.29 (s, 2H, COCH_2_), 2.75 (s, 4H, Morpholine-2CH_2_). ^13^ C NMR: δ 167.93, 163.97, 149.28, 143.29, 140.70, 130.00, 128.87, 125.21, 122.33, 119.75, 119.38, 111.23, 66.87, 62.32, 53.75. MS m/z (%); 373.12 (M^+^ + 2, 23.29), 371.17 (M^+^, 57.25), 100.30 (100.00).

#### *N-(4-(5-*chlorobenzoxazol*-2-yl)phenyl)-2-(4-methylpiperazin-1-yl)acetamide*(8):

Light pink solid, yield (55%), m.p. 179–181 °C. ^1^H NMR (400 MHz, CDCl_3_) δ 9.37 (s, 1H, NHCO), 8.23 (d, *J* = 8.6 Hz, 2H, Ar–H), 7.78 (d, *J* = 8.6 Hz, 2H, Ar–H), 7.74 (d, *J* = 1.8 Hz, 1H, Ar–H), 7.51 (d, *J* = 8.6 Hz, 1H, Ar–H), 7.33 (dd, *J* = 1.8, 8.6 Hz, 1H, Ar–H), 3.22 (s, 2H, COCH_2_), 2.80—2.60 (m, 8H, Piperazine-4CH_2_), 2.42 (s, 3H,-CH_3_). ^13^ C NMR: δ 168.70, 164.06, 149.30, 143.33, 140.86, 130.02, 128.90, 125.20, 122.19, 119.76, 119.31, 111.23, 61.87, 55.21, 53.37, 45.93. MS m/z (%); 386.00 (M^+^ + 2, 36.81), 384.34 (M^+^, 100.00).

#### *N-(4-(5-*chlorobenzoxazol*-2-yl)phenyl)-2-(4-ethylpiperazin-1-yl)acetamide*(9):

Light brown solid, yield (58%), m.p. 153–155 °C. ^1^H NMR (400 MHz, CDCl_3_) δ 9.40 (s, 1H, NHCO), 8.23 (d, *J* = 8.6 Hz, 2H, Ar–H), 7.79 (d, *J* = 8.6 Hz, 2H, Ar–H), 7.74 (d, *J* = 1.8 Hz, 1H, Ar–H), 7.51 (d, *J* = 8.6 Hz, 1H, Ar–H), 7.33 (dd, *J* = 1.8, 8.6 Hz, 1H, Ar–H), 3.22 (s, 2H, COCH_2_ ), 2.80—2.49 (m, 10H, C**H**_**2**_CH_3_, Piperazine-4CH_2_,), 1.16 (t, *J* = 7.2 Hz, 3H,CH_3_). ^13^ C NMR: δ 168.70, 164.06, 149.30,143.33, 140.88, 130.01, 128.89, 125.19, 122.18, 119.76, 119.32, 111.23, 61.91, 53.32, 52.87, 52.27, 11.88. MS m/z (%); 400.33 (M^+^ + 2, 19.64), 398.30 (M^+^, 100.00).

#### *2-(4-benzylpiperazin-1-yl)-N-(4-(5-chlorobenzoxazol-2-yl)phenyl)acetamide*(10):

Brown solid, yield (70%), m.p. 195–197 °C. ^1^H NMR (400 MHz, CDCl_3_) δ 9.41 (s, 1H, NHCO), 8.21 (d, *J* = 8.3 Hz, 2H, Ar–H), 7.79 (d, *J* = 8.3 Hz, 2H, Ar–H), 7.73 (s, 1H, Ar–H), 7.50 (d, *J* = 8.6 Hz, 1H, Ar–H), 7.48—7.30 (m, 6H, Ar–H), 3.77 (s, 2H, Phenyl-CH_2_ ), 3.27 (s, 2H, COCH_2_), 3.07—2.58 (m, 8H, Piperazine-4CH_2_). ^13^ C NMR: δ 168.57, 164.05, 149.29, 143.31, 140.84, 130.02, 129.47, 128.88, 128.51, 127.70, 125.21, 122.21, 119.75, 119.37, 111.24, 62.66, 61.81, 53.05, 52.92. MS m/z (%); 462.05 (M^+^ + 2, 26.35), 460.20 (M^+^, 26.73), 459.60 (100.00).

#### *N-(4-(5-*chlorobenzoxazol*-2-yl)*phenyl*)-3-(piperidin-1-yl)propanamide*(11):

Light brown solid, yield (57%), m.p. 161–163 °C.^1^H NMR (400 MHz, CDCl_3_) δ 11.54 (s, 1H, NHCO), 8.19 (d, *J* = 8.6 Hz, 2H, Ar–H), 7.76 (d, *J* = 8.6 Hz, 2H, Ar–H), 7.73 (d, *J* = 1.7 Hz, 1H, Ar–H), 7.50 (d, *J* = 8.6 Hz, 1H, Ar–H), 7.31 (dd, *J* = 1.7, 8.6 Hz, 1H, Ar–H), 2.85 (t, *J* = 5.6 Hz, 2H, COCH_2_CH_2_), 2.81—2.57 (m, 6H, COCH_2_CH_2_, Piperidine-2CH_2_ ), 1.86—1.76 (m, 4H, Piperidine-2CH_2_), 1.63 (s, 2H, Piperidine-CH_2_). ^13^ C NMR: δ 171.15, 164.29, 149.28, 143.41, 142.35, 129.91, 128.87, 125.01, 121.44, 119.68, 119.41, 111.17, 54.16, 53.64, 32.55, 26.16, 24.08. MS m/z (%); 384.93 (M^+^ + 2, 7.86), 383.39 (M^+^, 9.49), 55.17 (100.00).

#### *N-(4-(5-*chlorobenzoxazol*-2-yl)phenyl)-3-morpholinopropanamide*(12):

Dark brown solid, yield (56%), m.p. 204–206 °C.^1^H NMR (400 MHz, CDCl_3_) δ 11.18 (s, 1H, NHCO), 8.19 (d, *J* = 8.6 Hz, 2H, Ar–H), 7.74—7.69 (m, 3H, Ar–H), 7.49 (d, *J* = 8.6 Hz, 1H, Ar–H), 7.31 (dd, *J* = 2.0, 8.6 Hz, 1H, Ar–H), 3.88 (t, *J* = 4.1 Hz, 4H, Morpholine-2CH_2_), 2.80 (t, *J* = 5.8 Hz, 2H, COCH_2_CH_2_ ), 2.68 (s, 4H, Morpholine-2CH_2_), 2.61 (t, J = 5.8 Hz, 2H, COCH_2_CH_2_). ^13^ C NMR: δ 170.62, 164.12, 149.27, 143.36, 141.99, 129.93, 128.89, 125.08, 121.71, 119.70, 119.38, 111.20, 67.07, 54.05, 52.80, 32.28. MS m/z (%); 387.00 (M^+^ + 2, 23.03), 385.10 (M^+^, 88.25), 342.01 (100.00).

#### *N-(4-(5-chlorobenzoxazol-2-yl)phenyl)-3-(4-methylpiperazin-1-yl)propanamide*(13):

Brown solid, yield (63%), m.p. 191–193 °C.^1^H NMR (400 MHz, CDCl_3_) δ 11.25 (s, 1H, NHCO), 8.11 (d, *J* = 8.6 Hz, 2H, Ar–H), 7.68—7.62 (m, 3H, Ar–H), 7.41 (d, *J* = 8.6 Hz, 1H, Ar–H), 7.23 (dd, *J* = 1.9, 8.6 Hz, 1H, Ar–H), 2.76—2.46 (m, 12H, COCH_2_CH_2_, Piperazine-4CH_2_), 2.32 (s, 3H, CH_3_). ^13^ C NMR: δ 170.93, 164.22, 149.28, 143.37, 142.16, 129.94, 128.89, 125.06, 121.58, 119.70, 119.42, 111.18, 55.36, 53.50, 52.25, 46.04, 32.52. MS m/z (%); 400.13 (M^+^ + 2, 42.79), 398.33 (M^+^, 100.00).

#### *N-(4-(5-chlorobenzoxazol-2-yl)phenyl)-3-(4-ethylpiperazin-1-yl)propanamide*(14):

Dark orange solid, yield (81%), m.p. 167–169 °C. ^1^H NMR (400 MHz, CDCl_3_) δ 11.45 (s, 1H, NHCO, exchangeable), 8.19 (d, *J* = 8.5 Hz, 2H, Ar–H), 7.75—7.70 (m, 3H, Ar–H), 7.49 (d, *J* = 8.6 Hz, 1H, Ar–H), 7.31 (dd, *J* = 1.6, 8.6 Hz, 1H, Ar–H), 2.85—2.48 (m, 14H, C**H**_**2**_CH_3_, COCH_2_CH_2_, Piperazine-4CH_2_), 1.16 (t, *J* = 7.2 Hz, 3H, CH_3_). ^13^ C NMR: δ 170.96, 164.22, 149.28, 143.38, 142.20, 129.92, 128.87, 125.04, 121.54, 119.69, 119.40, 111.17, 53.51, 53.09, 52.32, 52.29, 32.52, 12.05. MS m/z (%); 414.24 (M^+^ + 2, 42.38), 412.40 (M^+^, 50.79), 411.74 (100.00).

#### *3-(4-benzylpiperazin-1-yl)-N-(4-(5-chlorobenzoxazol-2-yl)phenyl)propanamide*(15):

Light brown solid, yield (59%), m.p. 182–184 °C. (^1^H NMR (400 MHz, CDCl_3_) δ 10.99 (s, 1H, NHCO), 8.19 (d, *J* = 8.6 Hz, 2H, Ar–H), 7.77 (d, *J* = 8.6 Hz, 2H, Ar–H), 7.74 (d, *J* = 1.8 Hz, 1H, Ar–H), 7.50 (d, *J* = 8.6 Hz, 1H, Ar–H), 7.39—7.30 (m, 6H, Ar–H), 3.65 (s, 2H, Phenyl-CH_2_), 3.04—2.70 (m, 12H , COCH_2_CH_2_, Piperazine-4CH_2_). ^13^ C NMR: δ 170.83, 164.24, 149.30, 143.42, 142.19, 137.43, 129.94, 129.23, 128.88, 128.39, 127.39, 125.06, 121.57, 119.72, 119.42, 111.18, 62.87, 53.47, 53.06, 52.30, 32.53. MS m/z (%); 476.42 (M^+^ + 2, 1.52), 474.35 (M^+^, 7.21), 298.06 (100.00).

#### *General procedure for synthesis of compounds*(16–19):

A mixture of 2-chloro-N-(4-(5-chlorobenzoxazol-2-yl)phenyl)acetamide **(4)** or 3-chloro-N-(4-(5-chlorobenzoxazol-2-yl)phenyl)propanamide **(5)** (1.5 mmol), anhydrous potassium carbonate (0.2 g, 1.5 mmol) and phenyl piperazine or its derivative (1.8 mmol) was stirred under reflux in acetone / acetonitrile mixture (1:1) at 75 °C for 10–16 h. After completion of the reaction, the solvent was evaporated under pressure and the obtained residue was washed several times with ice-water, dried and recrystallized from ethanol to yield compounds **16–19**.

#### *N-(4-(5-chlorobenzoxazol-2-yl)phenyl)-2-(4-phenylpiperazin-1-yl)acetamide*(16):

Light orange solid, yield (54%), m.p. 231–233 °C. ^1^H NMR (400 MHz, CDCl_3_) δ 9.43 (s, 1H, NHCO), 8.24 (d, *J* = 8.6 Hz, 2H, Ar–H), 7.79 (d, *J* = 8.6 Hz, 2H, Ar–H), 7.74 (d, *J* = 1.7 Hz, 1H, Ar–H), 7.51 (d, *J* = 8.6 Hz, 1H, Ar–H), 7.35—7.29 (m, 3H, Ar–H), 7.01—6.91 (m, 3H, Ar–H), 3.32 (t, *J* = 4.6 Hz, 4H, Piperazine-2CH_2_ ), 3.28 (s, 2H, COCH_2_ ), 2.85 (t, J = 4.6 Hz, 4H, Piperazine-2CH_2_). ^13^ C NMR: δ 168.49, 164.02, 150.90, 149.30, 143.32, 140.81, 130.01, 129.26, 128.91, 125.20, 122.26, 120.34, 119.76, 119.34, 116.33, 111.24, 62.00, 53.57, 49.52. MS m/z (%); 448.20 (M^+^ + 2, 0.7), 446.12 (M^+^, 2.04), 70.22 (100.00).

#### *N-(4-(5-*chlorobenzoxazol*-2-yl)phenyl)-2-(4-(2-methoxyphenyl)piperazin-1-yl)acetamide*(17):

Light brown solid, yield (67%), m.p. 218–220 °C. ^1^H NMR (400 MHz, CDCl_3_) δ 9.49 (s, 1H, NHCO, exchangeable), 8.24 (d, *J* = 8.6 Hz, 2H, Ar–H), 7.81 (d, *J* = 8.6 Hz, 2H, Ar–H), 7.74 (d, *J* = 1.7 Hz, 1H, Ar–H), 7.51 (d, *J* = 8.6 Hz, 1H, Ar–H), 7.33 (dd, *J* = 1.7, 8.6 Hz, 1H, Ar–H), 7.10—6.89 (m, 4H, Ar–H), 3.91 (s, 3H, OCH_3_), 3.29 (s, 2H, COCH_2_), 3.21 (s, 4H, Piperazine-2CH_2_), 2.89 (t, *J* = 4.2 Hz, 4H, Piperazine-2CH_2_). ^13^ C NMR: δ 168.74, 164.07, 152.27, 149.31, 143.34, 140.90, 140.74, 130.01, 128.91, 125.19, 123.41, 122.20, 121.04, 119.77, 119.34, 118.25, 111.33, 111.23, 62.08, 55.46, 53.82, 50.87. MS m/z (%); 478.35 (M^+^ + 2, 2.28), 476.36 (M^+^, 2.28), 62.16 (100.00).

#### *N-(4-(5-chlorobenzoxazol-2-yl)phenyl)-3-(4-phenylpiperazin-1-yl)propanamide*(18):

Grey solid, yield (52%), m.p. 239–241 °C. ^1^H NMR (400 MHz, CDCl_3_) δ 11.27 (s, 1H, NHCO), 8.18 (d, *J* = 8.6 Hz, 2H, Ar–H), 7.75—7.69 (m, 3H, Ar–H), 7.50 (d, *J* = 8.5 Hz, 1H, Ar–H), 7.39—7.30 (m, 3H, Ar–H), 7.07—6.93 (m, 3H, Ar–H), 3.39 (s, 4H, Piperazine-2CH_2_), 2.93—2.84 (m, 6H, COCH2CH2, Piperazine-2CH_2_), 2.68 (t, *J* = 5.5 Hz, 2H, COCH_2_CH_2_). ^13^ C NMR: δ 170.70, 164.16, 150.73, 149.28, 143.39, 142.03, 129.93, 129.35, 128.90, 125.07, 121.71, 120.53, 119.72, 119.41, 116.29, 111.19, 53.63, 52.46, 49.46, 32.61. MS m/z (%); 462.52 (M^+^ + 2, 4.32), 460.66 (M^+^, 30.05), 55.24 (100.00).

#### *N-(4-(5-chlorobenzoxazol-2-yl)phenyl)-3-(4-(2-methoxyphenyl)piperazin-1-yl)propanamide*(19):

Light brown solid, yield (56%), m.p. 211–213 °C. ^1^H NMR (400 MHz, CDCl_3_) δ 11.51 (s, 1H, NHCO, exchangeable), 8.19 (d, *J* = 8.6 Hz, 2H, Ar–H), 7.77—7.72 (m, 3H, Ar–H), 7.50 (d, *J* = 8.6 Hz, 1H, Ar–H), 7.31 (dd, *J* = 1.4, 8.6 Hz, 1H, Ar–H), 7.12—6.91 (m, 4H, Ar–H), 3.92 (s, 3H, OCH_3_), 3.27 (s, 4H, Piperazine-2CH_2_), 2.98—2.82 (m, 6H, COCH_2_CH_2_, Piperazine-2CH_2_ ), 2.64 (t, J = 5.7 Hz, 2H, COCH_2_CH_2_ ). ^13^ C NMR: δ 170.94, 164.21, 152.26, 149.28, 143.39, 142.19, 140.56, 129.91, 128.89, 125.04, 123.56, 121.59, 121.09, 119.70, 119.42, 118.26, 111.35, 111.19, 55.47, 53.64, 52.59, 50.92, 32.55. MS m/z (%); 492.33 (M^+^ + 2, 1.31), 490.25 (M^+^, 7.95), 55.20 (100.00).

#### *General procedure for synthesis of compounds*(20–25):

A mixture of 2-chloro-N-(4-(5-chlorobenzoxazol-2-yl)phenyl)acetamide **(4)** or 3-chloro-N-(4-(5-chlorobenzoxazol-2-yl)phenyl)propanamide **(5)** (1.5 mmol), anhydrous potassium carbonate (0.2 g, 1.5 mmol) and the appropriate arylalkylamine (1.8 mmol) was stirred under reflux in dioxane at 90 °C for 10–16 h. After completion of the reaction, the solvent was evaporated under pressure and the obtained residue was washed several times with ice-water and dried. The dried residue was recrystallized from ethanol to yield compounds **20, 21, 23** and **24** or purified using preparative TLC with elution system of (hexane/ethyl acetate) (8:3) to yield compounds **22** and **25**.

#### *2-(benzylamino)-N-(4-(5-chlorobenzoxazol-2-yl)phenyl)acetamide*(20):

Dark brown solid, yield (61%), m.p. 158–160 °C. ^1^H NMR (400 MHz, CDCl_3_) δ 9.56 (s, 1H, CONH), 8.22 (d, *J* = 8.6 Hz, 2H, Ar–H), 7.77 (d, *J* = 8.6 Hz, 2H, Ar–H), 7.74 (d, *J* = 1.8 Hz, 1H, Ar–H), 7.51 (d, *J* = 8.6 Hz, 1H, Ar–H), 7.45—7.30 (m, 6H, Ar–H), 3.90 (s, 2H, Phenyl-CH_2_ ), 3.50 (s, 2H, COCH_2_ ). ^13^ C NMR: δ 170.00, 164.12, 149.30, 143.36, 140.88, 138.86, 129.98, 128.86, 128.14, 127.72, 125.15, 122.10, 119.75, 119.28, 111.22, 54.18, 52.49. MS m/z (%); 393.11 (M^+^ + 2, 5.84), 391.09 (M^+^, 21.46), 91.22 (100.00).

#### *N-(4-(5-chlorobenzoxazol-2-yl)*phenyl*)-2-(phenethylamino)acetamide*(21):

Light brown solid, yield (82%), m.p. 129–131 °C. ^1^H NMR (400 MHz, CDCl_3_) δ 9.36 (s, 1H, CONH, exchangeable), 8.17 (d, *J* = 8.6 Hz, 2H, Ar–H), 7.74 (d, *J* = 1.8 Hz, 1H, Ar–H), 7.55—7.49 (m, 3H, Ar–H), 7.41—7.30 (m, 5H, Ar–H), 7.27 (s, 1H, Ar–H), 3.43 (s, 2H, COCH_2_), 3.02 (t, *J* = 6.3 Hz, 2H, Phenyl-CH_2_-CH_2_), 2.86 (t, *J* = 6.3 Hz, 2H, Phenyl-CH_2_-CH_2_). ^13^ C NMR: δ 170.18, 164.19, 149.30, 143.36, 140.84, 139.55, 129.98, 128.93, 128.79, 128.72, 126.57, 125.13, 121.92, 119.73, 119.24, 111.20, 52.67, 51.17, 36.36. MS m/z (%); 407.60 (M^+^ + 2, 2.61), 405.19 (M^+^, 11.05), 314.05 (100.00).

#### *N-(4-(5-chlorobenzoxazol-2-yl)*phenyl*)-2-((3-phenylpropyl)amino)acetamide*(22):

Dark grey solid, yield (55%), m.p. 93–95 °C. ^1^H NMR (400 MHz, CDCl_3_) δ 9.62 (s, 1H, CONH), 8.22 (d, *J* = 8.7 Hz, 2H, Ar–H), 7.78 (d, *J* = 8.7 Hz, 2H, Ar–H), 7.74 (d, *J* = 2 Hz, 1H, Ar–H), 7.50 (d, *J* = 8.6 Hz, 1H, Ar–H), 7.35—7.29 (m, 3H, Ar–H), 7.26—7.19 (m, 3H, Ar–H), 3.43 (s, 2H, COCH_2_), 2.79—2.71 (m, 4H, Phenyl-CH_2_-CH_2_-CH_2_), 1.96—1.87 (qui, 2H, Phenyl-CH_2_-CH_2_-CH_2_). ^13^ C NMR: δ 170.30, 164.13, 149.30, 143.36, 141.44, 140.92, 129.98, 128.88, 128.55, 128.32, 126.13, 125.15, 122.08, 119.75, 119.29, 111.22, 53.03, 49.88, 33.47, 31.72. MS m/z (%); 421.46 (M^+^ + 2, 5.95), 419.35 (M^+^, 36.75), 244.22 (100.00).

#### *3-(benzylamino)-N-(4-(5-chlorobenzoxazol-2-yl)phenyl)propanamide*(23):

Grey solid, yield (76%), m.p. 109–111 °C. ^1^H NMR (400 MHz, CDCl_3_) δ 11.06 (s, 1H, CONH, exchangeable), 8.19 (d, *J* = 8.7 Hz, 2H, Ar–H), 7.73 (d, *J* = 1.8 Hz, 1H, Ar–H), 7.71 (d, *J* = 8.7 Hz, 2H, Ar–H), 7.50 (d, *J* = 8.6 Hz, 1H, Ar–H), 7.45—7.30 (m, 6H, Ar–H), 3.93 (s, 2H, Phenyl-CH_2_), 3.09 (t, J = 5.6 Hz, 2H, COCH_2_CH_2_), 2.59 (t, J = 5.6 Hz, 2H, COCH_2_CH_2_). ^13^ C NMR: δ 171.23, 164.29, 149.29, 143.40, 142.01, 138.81, 129.94, 128.86, 128.81, 128.30, 127.74, 125.05, 121.55, 119.70, 119.58, 111.18, 53.43, 44.76, 36.04. MS m/z (%); 407.66 (M^+^ + 2, 10.26), 405.03 (M^+^,18.35), 258.72 (100.00).

#### *N-(4-(5-chlorobenzoxazol-2-yl)*phenyl*)-3-(phenethylamino)propanamide*(24):

Light pink solid, yield (79%), m.p. 73–75 °C.^1^H NMR (400 MHz, CDCl_3_) δ 11.00 (s, 1H, CONH), 8.12 (d, *J* = 8.7 Hz, 2H, Ar–H), 7.74 (d, *J* = 1.9 Hz, 1H, Ar–H), 7.50 (d, *J* = 8.6 Hz, 1H, Ar–H), 7.43 (d, J = 8.7 Hz, 2H, Ar–H), 7.38—7.29 (m, 5H, Ar–H), 7.28—7.25 (m, 1H, Ar–H), 3.07 (t, *J* = 6.5 Hz, 2H, Phenyl-CH_2_-CH_2_), 3.03 (t, *J* = 5.7 Hz, 2H, COCH_2_CH_2_), 2.93 (t, *J* = 6.5 Hz, 2H, Phenyl-CH_2_-CH_2_), 2.52 (t, *J* = 5.7 Hz, 2H, COCH_2_CH_2_ ). ^13^ C NMR: δ 171.31, 164.33, 149.28, 143.40, 142.01, 139.35, 129.91, 128.86, 128.68, 126.65, 125.02, 121.37, 119.66, 119.53, 111.17, 50.08, 45.24, 36.22, 35.96. MS m/z (%); 421.40 (M^+^ + 2, 19.69), 419.01 (M^+^, 29.86), 148.94 (100.00).

#### *N-(4-(5-chlorobenzoxazol-2-yl)phenyl)-3-((3-phenylpropyl)*amino*)propanamide*(25):

Light violet solid, yield (53%), m.p. 87–89 °C. ^1^H NMR (400 MHz, CDCl_3_) δ 11.21 (s, 1H, CONH), 8.19 (d, *J* = 8.6 Hz, 2H, Ar–H), 7.74—7.70 (m, 3H, Ar–H), 7.50 (d, *J* = 8.6 Hz, 1H, Ar–H), 7.37—7.30 (m, 3H, Ar–H), 7.27—7.21 (m, 3H, Ar–H), 3.03 (t, *J* = 5.6 Hz, 2H, COCH_2_CH_2_), 2.83—2.73 (m, 4H, Phenyl-CH_2_-CH_2_-CH_2_), 2.55 (t, *J* = 5.6 Hz, 2H, COCH_2_CH_2_), 2.01—1.92 (qui, 2H, Phenyl-CH_2_-**CH**_**2**_-CH_2_). ^13^ C NMR: δ 171.40, 164.28, 149.29, 143.39, 142.09, 141.43, 129.92, 128.83, 128.58, 128.30, 126.16, 125.04, 121.52, 119.69, 119.54, 111.18, 48.67, 45.19, 35.96, 33.68, 31.64. MS m/z (%); 435.20 (M^+^ + 2, 1.21), 433.69 (M^+^, 8.00), 298.12 (100.00).

#### *General procedure for synthesis of compounds*(26, 27):

A mixture of 2-chloro-N-(4-(5-chlorobenzoxazol-2-yl)phenyl)acetamide **(4)** or 3-chloro-N-(4-(5-chlorobenzoxazol-2-yl)phenyl)propanamide **(5)** (1.5 mmol), anhydrous potassium carbonate (0.2 g, 1.5 mmol,) and diethylamine (1.8 mmol) was stirred under reflux in acetone / acetonitrile mixture (1:1) at 75 °C for 10–16 h. After completion of the reaction, the solvent was evaporated under pressure and the obtained residue was washed several times with ice-water, dried and recrystallized from ethanol to yield compounds **26, 27**.

#### *N-(4-(5-chlorobenzoxazol-2-yl)phenyl)-2-(diethylamino)acetamide*(26):

Orange solid, yield (50%), m.p. 133–135 °C. ^1^H NMR (400 MHz, DMSO-d_6_) δ 10.13 (s, 1H, NHCO, exchangeable), 8.17 (d, *J* = 8.6 Hz, 2H, Ar–H), 7.94 (d, *J* = 8.6 Hz, 2H, Ar–H), 7.90 (d, *J* = 1.8 Hz, 1H, Ar–H), 7.82 (d, *J* = 8.6 Hz, 1H, Ar–H), 7.47 (dd, *J* = 1.8, 8.6 Hz, 1H, Ar–H), 3.31 (s, 2H, COCH_2_), 2.69 (s, 4H, 2-**CH**_**2**_CH_3_), 1.07 (t, J = 7.0 Hz, 6H, 2-CH_2_**CH**_**3**_). ^13^ C NMR: δ 170.79, 164.18, 149.44, 143.44, 142.52, 129.44, 128.92, 125.66, 121.04, 120.00, 119.72, 112.63, 57.53, 48.31, 12.14. MS m/z (%); 359.15 (M^+^ + 2, 15.34), 357.17 (M^+^, 40.76), 86.19 (100.00).

#### *N-(4-(5-chlorobenzoxazol-2-yl)phenyl)-3-(diethylamino)propanamide*(27):

Light brown solid, yield (55%), m.p. 84–86 °C. ^1^H NMR (400 MHz, DMSO-d_6_) δ 10.53 (s, 1H,NH, exchangeable), 8.14 (d, *J* = 8.3 Hz, 2H, Ar–H), 7.91—7.77 (m, 4H, Ar–H), 7.44 (d, *J* = 8.5 Hz, 1H, Ar–H), 2.76 (t, *J* = 6.7 Hz, 2H, COCH_2_CH_2_), 2.57—2.44 (m, 8H, COCH_2_CH_2_, 2-**CH**_**2**_CH_3_), 0.98 (t, *J* = 7.0 Hz, 6H, 2-CH_2_**CH**_**3**_). ^13^ C NMR: δ 171.65, 164.24, 149.42, 143.47, 143.37, 129.41, 129.00, 125.57, 120.55, 119.68, 119.52, 112.59, 48.67, 46.54, 34.78, 12.28. MS m/z (%); 373.18 (M^+^ + 2, 33.36), 371.25 (M^+^, 100.00).

#### *Synthesis of 2-(4-*azidophenyl*)-5-chlorobenzoxazole*(28):

4-(5-chlorobenzoxazol-2-yl)aniline **3**, (0.73 g, 3 mmol) was dissolved in ethanol (30 ml) then a solution of p-toluene sulfonic acid (*p*-TsOH) (5.1 g, 27 mmol) in 27 ml H_2_O was added and stirred for 1 min. After that, NaNO_2_ (1.9 g, 27 mmol) was added gradually within 5 min and the resulting solution was stirred at room temperature for 30 min till the disappearance of compound **3** (monitored by TLC). NaN_3_ (0.3 g, 4.8 mmol) was added to the resulting solution at room temperature. The separated solid was filtered, washed with H_2_O, dried, extracted with ethyl acetate and then recrystallized from ethanol to yield compound **28**.

#### *2-(4-azidophenyl)-5-chlorobenzoxazole*(28):

Dark orange solid, yield (75%), m.p. 167–169 °C. IR υ_max_/ cm^-1^: 2128 (N_3_). ^1^H NMR (400 MHz, CDCl_3_) δ 8.25 (d, *J* = 8.4 Hz, 2H, Ar–H), 7.76 (s, 1H, Ar–H), 7.52 (d, *J* = 8.6 Hz, 1H, Ar–H), 7.35 (d, *J* = 8.6 Hz, 1H, Ar–H), 7.20 (d, *J* = 8.4 Hz, 2H, Ar–H). MS m/z (%); 272.22 (M^+^ + 2, 5.74), 270.04 (M^+^, 12.07), 242.05 (100.00).

#### *General procedure for *synthesis* of ethyl 1-(4-(5-chlorobenzoxazol-2-yl)phenyl)-5-methyl-1H-1,2,3-triazole-4-carboxylate (29) and 1-(1-(4-(5-chlorobenzoxazol-2-yl)phenyl)-5-methyl-1H-1,2,3-triazol-4-yl)ethan-1-one*(30):

2-(4-azidophenyl)-5-chlorobenzoxazole **28** (0.54 g, 2 mmol), triethylamine (0.6 ml, 4 mmol) and ethyl acetoacetate or acetyl acetone (4 mmol) were dissolved in DMF (30 ml) and stirred at room temperature for 48–72 h. the separated solid was filtered, washed with H_2_O, dried, extracted with ethyl acetate and then recrystallized from ethanol to yield compounds **29** or **30**.

#### *Ethyl 1-(4-(5-chlorobenzoxazol-2-yl)phenyl)-5-methyl-1H-1,2,3-triazole-4-carboxylate*(29):

Gold solid, yield (71%), m.p. 251–253 °C. IR υ_max_/ cm^-1^: 1718 (C = O). ^1^H NMR (400 MHz, CDCl_3_) δ 8.48 (d, *J* = 8.1 Hz, 2H, Ar–H), 7.82 (s, 1H, Ar–H), 7.70 (d, *J* = 8.1 Hz, 2H, Ar–H), 7.58 (d, *J* = 8.6 Hz, 1H, Ar–H), 7.41 (d, *J* = 8.6 Hz, 1H, Ar–H), 4.51 (q, *J* = 7.0 Hz, 2H, **CH**_**2**_CH_3_), 2.71 (s, 3H, CH_3_), 1.49 (t, *J* = 7.0 Hz, 3H, CH_2_**CH**_**3**_). ^13^ C NMR: δ 162.64, 161.63, 149.50, 143.03, 138.85, 137.95, 137.17, 130.53, 129.05, 128.29, 126.18, 125.73, 120.34, 111.59, 61.31, 14.42, 10.23. MS m/z (%); 384.17 (M^+^ + 2, 44.24), 382.09 (M^+^, 100.00).

#### *1-(1-(4-(5-chlorobenzoxazol-2-yl)*phenyl*)-5-methyl-1H-1,2,3-triazol-4-yl)ethan-1-one*(30):

Silver solid, yield (65%), m.p. 212–214 °C. IR υ_max_/ cm^-1^: 1687 (C = O). ^1^H NMR (400 MHz, CDCl_3_) δ 8.39 (d, *J* = 8.6 Hz, 2H, Ar–H), 7.72 (d, *J* = 2.0 Hz, 1H, Ar–H), 7.60 (d, *J* = 8.6 Hz, 2H, Ar–H), 7.49 (d, *J* = 8.6 Hz, 1H, Ar–H), 7.32 (dd, *J* = 2.0, 8.6 Hz, 1H, Ar–H), 2.71 (s, 3H, CH_3_), 2.61 (s, 3H, COCH_3_) ^13^ C NMR: δ 194.36, 162.63, 149.50, 143.95, 143.06, 137.82, 137.39, 130.52, 129.05, 128.30, 126.16, 125.64, 120.35, 111.58, 28.00, 10.37. MS m/z (%); 354.11 (M^+^ + 2, 6.24), 352.05 (M^+^, 19.17), 43.14 (100.00).

### Biological evaluation

#### In vitro cytotoxicity screening

Two human breast cancer cell lines, namely, MDA-MB-231 and MCF-7 were obtained from American Type Culture Collection (ATCC) via a holding company for biological products and vaccines (VACSERA), Cairo, Egypt. Sorafenib was used as a standard anticancer drug for comparison. The reagents used were RPMI-1640 medium, MTT, DMSO (sigma co., St. Louis, USA), and Fetal bovine serum (GIBCO, UK). The cell lines were used to determine the inhibitory effect of the synthesized compounds **3–30** using MTT assay. MTT is a colorimetric assay based on the conversion of the yellow tetrazolium bromide (MTT) to a purple formazan derivative by mitochondrial succinate dehydrogenase in viable cells. Cell lines were cultured in RPMI-1640 medium with 10% foetal bovine serum. Antibiotics added were 100 units/ml penicillin and 100 µg/ml streptomycin maintained at 37 °C in a 5% CO_2_ incubator. The cell lines were seeded at a density of approximately 1.0 × 10 ^4^ cells/well in a 96-well plate at 37 °C for 48 h under 5% CO_2_. Different concentrations of compounds were added and incubated for 24 h. After 24 h of drug treatment, 20 µl of MTT staining solution at 5 mg/ml was added to each well and incubated for 4 h at 37 °C. After 4 h, 100 µl of dimethyl sulfoxide (DMSO) were added into each well to dissolve the purple formazan crystals formed. The absorbance was recorded at 570 nm using a plate reader (EXL 800, USA). The relative cell viability in percentage was calculated as (A570 of treated samples/A570 of untreated sample) X 100 ^22,23^.

#### Poly (ADP-ribose) polymerase 2 (PARP-2) inhibition assay

The PARP-2 inhibitory activity of the test compounds was assayed using PARP-2 Colorimetric Activity Assay Kit (BPS Bioscience, catalog 80,581, San Diego, CA, USA) following the manufacturer’s protocol. Briefly, histone protein mixture was coated on a 96-well microtiter plate and incubated at 4 °C overnight, washed with phosphate-buffered saline with tween-20 (PBST) three times then, the wells were blocked by adding blocking buffer 3 and incubated at room temperature for 60–90 min. After that, the plate was washed with PBST buffer three times. The master mixture which contains the PARP buffer, PARP assay mixture, activated DNA and distilled water was added to each well then, the inhibitor solution (test compound) was added. The reaction was initiated by adding PARP-2 enzyme to the wells and the plate was incubated for 1 h at room temperature. After 1 h, the reaction mixture was discarded, and the plate was washed with PBST buffer three times. Finally, the plate was treated with Streptavidin–Horseradish Peroxidase (HRP) and incubated for 30 min at room temperature and then washed with PBST buffer. The colorimetric HRP substrate was added to each well and incubated at room temperature until the positive control well developed blue color. Sulphuric acid was added to each well after the blue color development. The absorbance was read at 450 nm using UV/Vis spectrophotometer microplate reader.

#### Cell cycle analysis

MCF-7 cells were seeded in 24-well plate at a density of 2 × 10^5^ cells/well, treated with the test compounds **11, 12, 13** and **27** at different concentrations and incubated at 37 °C for 24 h in 5% CO_2_ atmosphere. The cells were washed with ice-cold phosphate buffer saline (PBS) twice and fixed in 70% ice-cold ethanol overnight and then suspended in (PBS) containing PI (DNA staining solution) at concentration of 50 µg/mL, 0.1 mg/mL RNAase A (Sigma, USA) and 0.05% Triton X-100. Cells were analysed by flow cytometry after 30 min at 37 n the dark using FACSCalibur (Becton Dickinson FACS, San Jose, CA) flow cytometer. The cell cycle distributions were measured using Cell-Quest software^34^.

#### Detection of apoptosis by flow cytometry

Cell death by apoptosis was detected by flow cytometric method using Annexin-V/FITC propidium iodide Apoptosis Detection Kit Catalog #: K101-25; Biovision, USA). MCF-7 cells were seeded at a density of 2 × 10 ^5^ cells/well in 24-well plate, treated with the test compounds **11, 12, 13** and **27** at different concentrations and incubated at 37 °C for 24 h in 5% CO_2_ atmosphere. Cells were collected and suspended in 500 µl binding buffer and then 5 µl of Annexin V-FITC and 5 µl of PI were added and mixed. The mixture was incubated for 5 min in the dark at room temperature. Cell apoptosis analyses were performed using FACSCalibur (Becton Dickinson FACS, San Jose, CA) flow cytometer^35^.

### Molecular modeling study

#### Molecular docking into the catalytic domain of PARP-2 enzyme

Molecular modeling process was done using molecular operating environment (MOE) software version 2009.10 Chemical Computing Group Inc. Compounds were built, energy minimized to get the most stable conformers. The active site of ARTD2 (PARP-2)—catalytic domain in complex with olaparib (PDB Code: 4TVJ) which was obtained from the research collaboratory for structural bioinformatics Protein Data Bank (RCSB) PDB. The protein was prepared for docking process and the active site of interest which contains the essential amino acids bound to olaparib was isolated. The most stable conformers of the compounds were docked into the active side. The docking process was performed using triangle matcher technique for placement step, London dG for rescoring and forcefield method for refinement. The 2D and 3D pictures of the best fitting poses were isolated.

#### 3D Ligand -Based Alignment in PARP-2 pocket

Ligands alignment inside PARP-2 binding pocket was isolated in 3D forms and surface maps were calculated using the surfaces and maps tool in MOE program according to activeLP format. The pink color refers to regions with hydrogen bonding affinity, the green color refers to the hydrophobic regions and the blue color represents mild polar regions.

#### Flexible alignment

Flexible alignment of the desired compounds was performed using the MMFF94 flexible alignment tool in MOE program. 100 iterations of each compound were generated, the energy cut-off was adjusted to 15 kcal/mol and the root-mean-squared deviation (RMSD) tolerance to 0.5^36^.

## Supplementary Information

Supplementary Information.

## Data Availability

The datasets generated during and/or analyzed during the current study are available from the corresponding author on request.
