# Supplementary material for: Synthesis, biological evaluation, and molecular modeling studies of new benzoxazole derivatives as PARP-2 inhibitors targeting breast cancer
Source: Sci Rep. 2022 Sep 28;12:16246. doi: 10.1038/s41598-022-20260-1 (PMC9519869; doi:10.1038/s41598-022-20260-1)

Synthesis, biological evaluation, and molecular modeling studies of new benzoxazole derivatives as PARP-2 inhibitors targeting breast cancer

Nadeen M. El-Ghobashy, Selwan M. El-Sayed,* Ihsan A. Shehata, Mahmoud B. El-Ashmawy

Department of Medicinal Chemistry Pharmacy, Faculty of Pharmacy, Mansoura University, Mansoura 35516, Egypt

Content	Page
**Suppl. S1.** NMR and IR spectra of the new synthesized compounds	**2**
**Suppl. S2.** *In vitro* PARP-2 enzyme inhibition assay	**31**
**Table S1.** PARP-2 enzyme inhibition results (IC_{50} µM) of compounds 11-14, 21, 22, 25-27 and olaparib	**31**
**Table S2.** Raw data of *in vitro* PARP-2 enzyme inhibition assay	**32**
**Fig. S1.** Calculations of % inhibition of compounds11-14, 21, 22, 25-27 and olaparib	**35**
**Suppl. S3.** Cell cycle analysis for compounds 11, 12, 13 and 27	**36**
**Table S3.** Cell cycle phase distribution in in MCF-7 cell line treated with vehicle control and the newly synthesised compounds; 11, 12, 13 and 27	**36**
**Fig. S2.** Cell cycle phase distribution in in MCF-7 cell line treated with vehicle control and the newly synthesised compounds; 11, 12, 13 and 27	**37**
**Suppl. S4.** Detection of apoptosis and necrosis for compounds 11, 12, 13 and 27	**37**
**Table S4.** Apoptosis percentage in MCF-7 cells treated with vehicle control and the newly synthesised compounds; 11, 12, 13 and 27	**37**
**Fig. S3.** Apoptosis percentage in MCF-7 cells treated with vehicle control and the newly synthesised compounds; 11, 12, 13 and 27	**37**

Suppl. S1. NMR and IR spectra of the new synthesized compounds

^{1}H NMR and ^{13}C NMR spectra of compound **4**

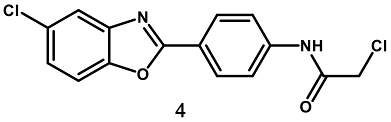

4

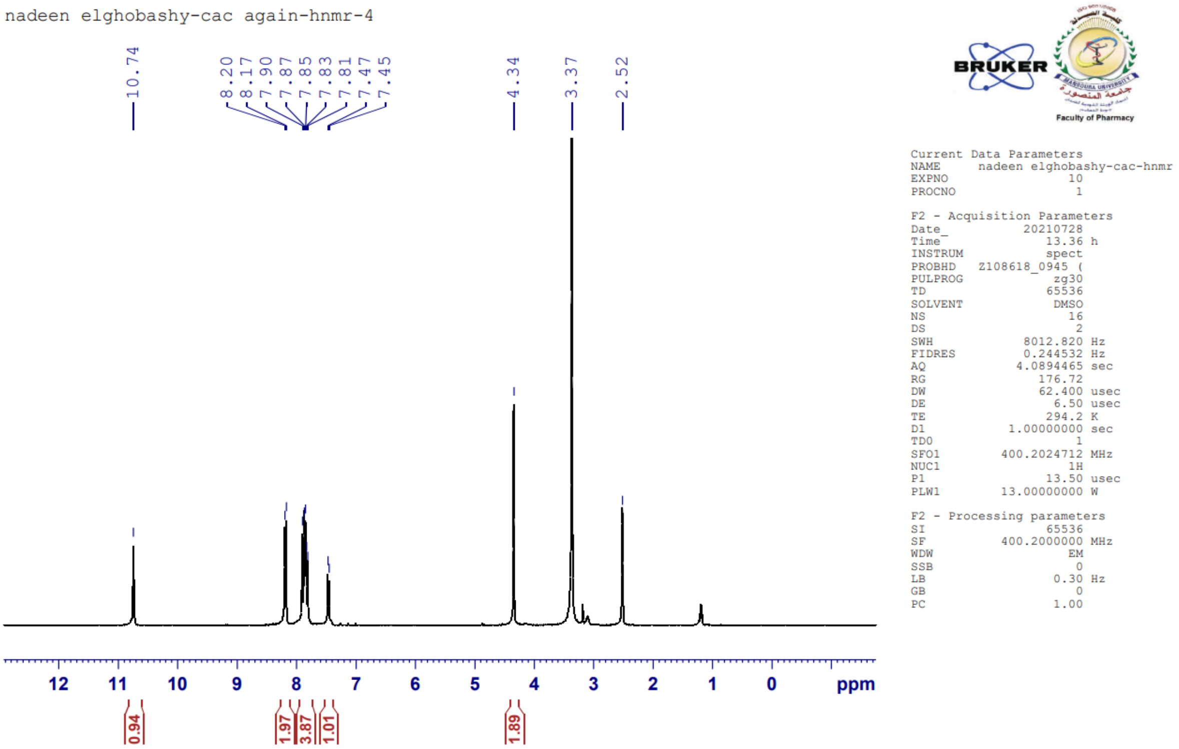

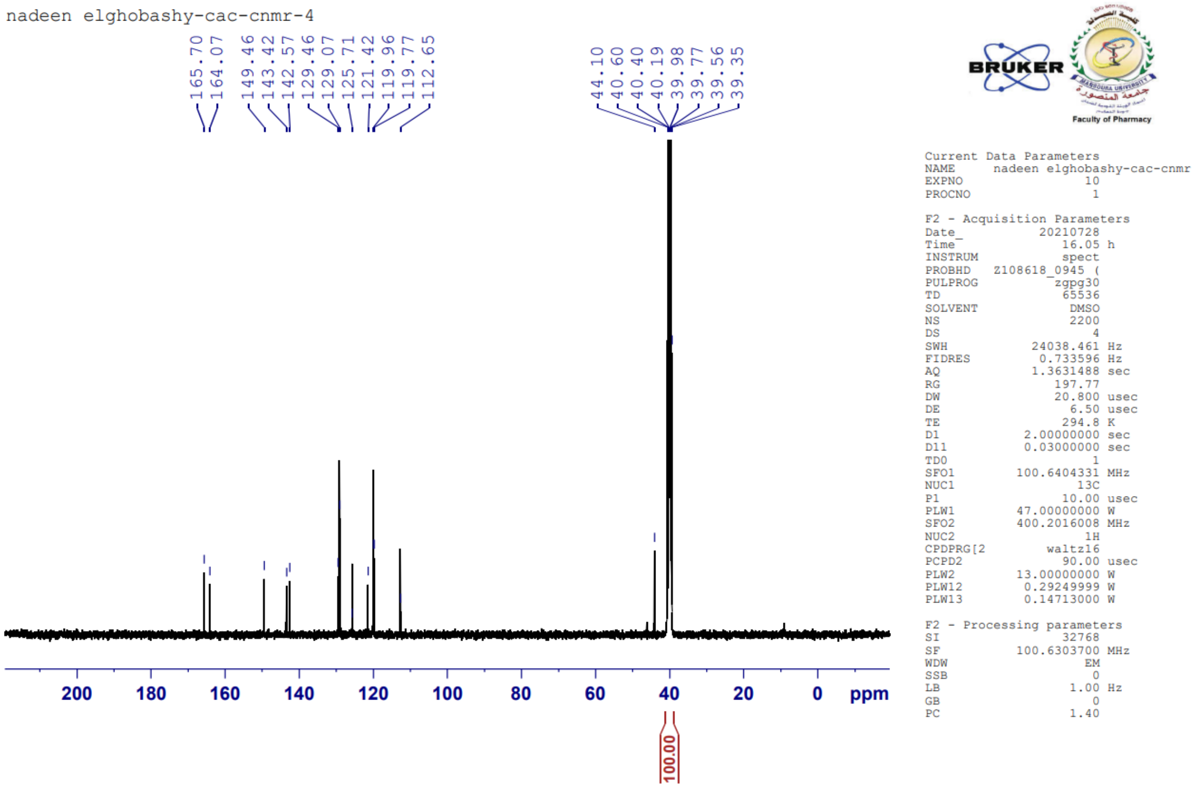

^{1}H NMR and ^{13}C NMR spectra of compound **5**

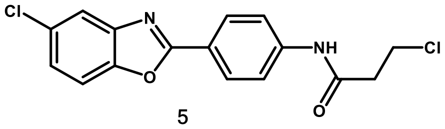

5

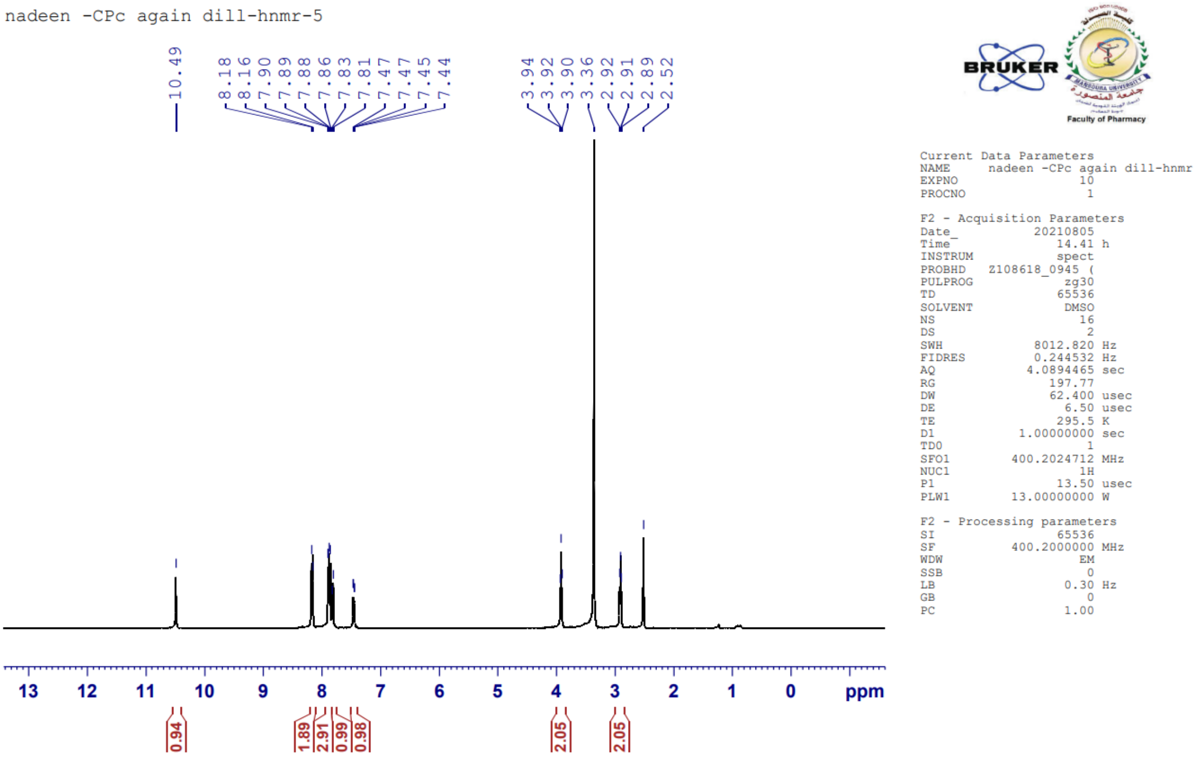

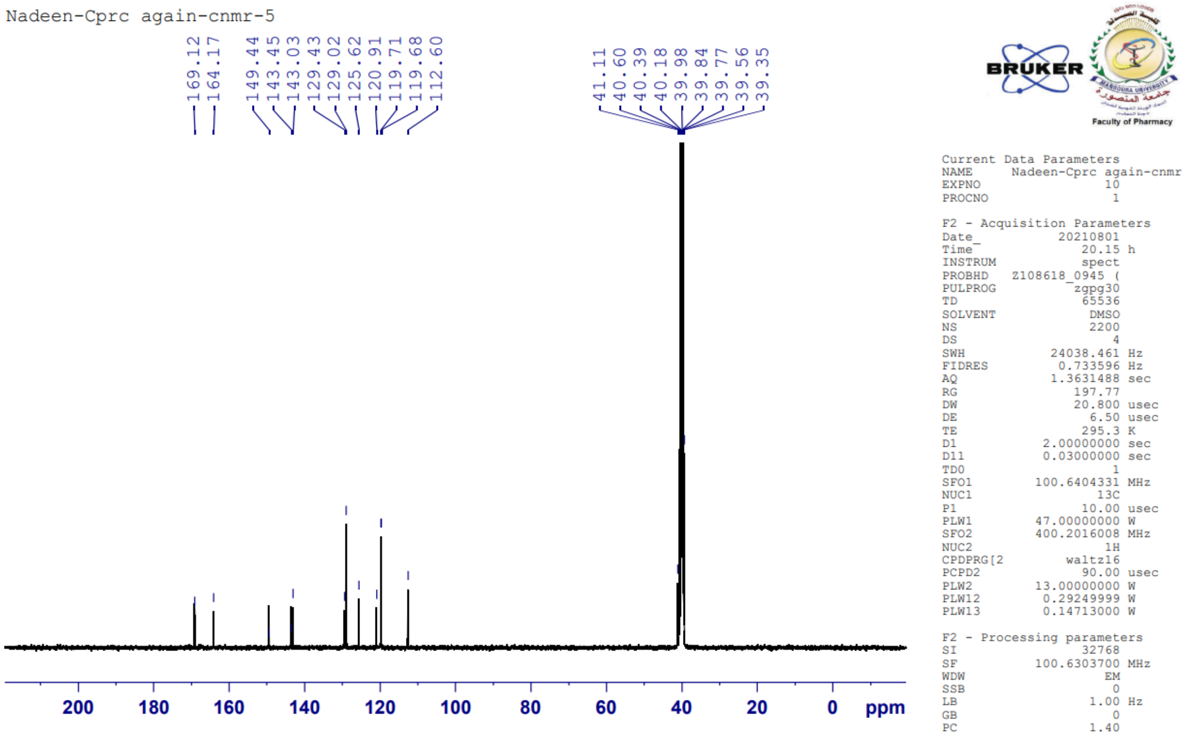

^{1}H NMR and ^{13}C NMR spectra of compound **6**

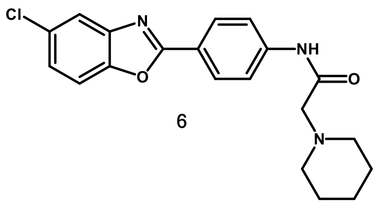

6

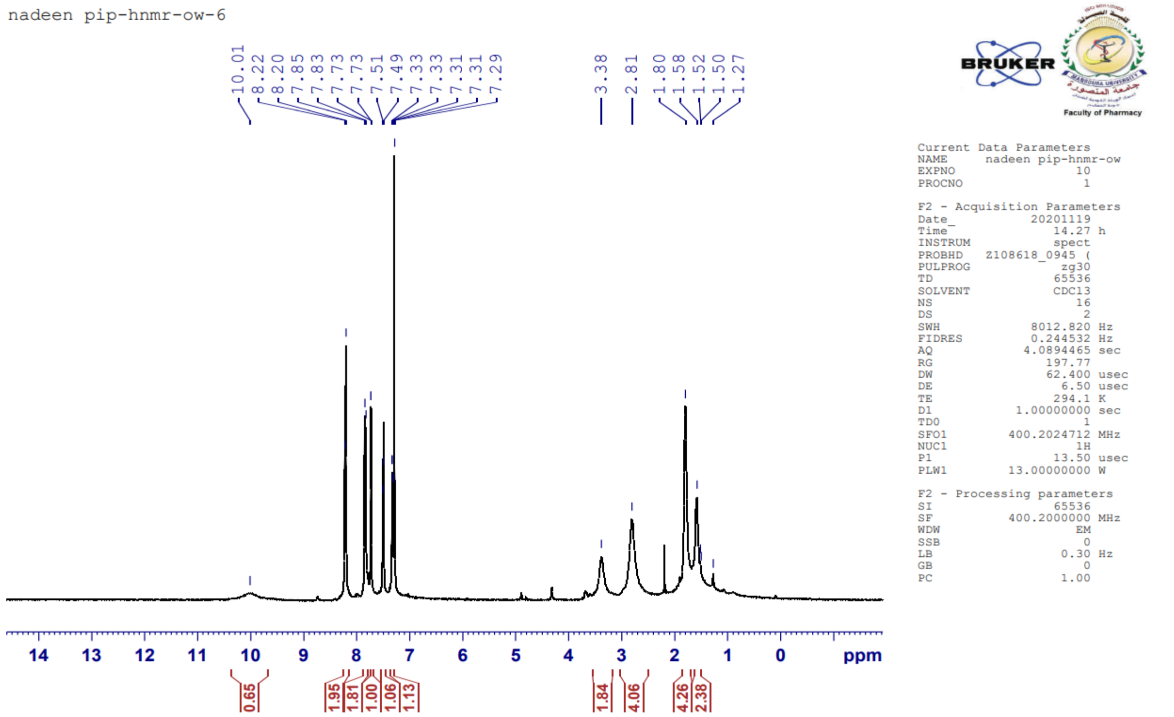

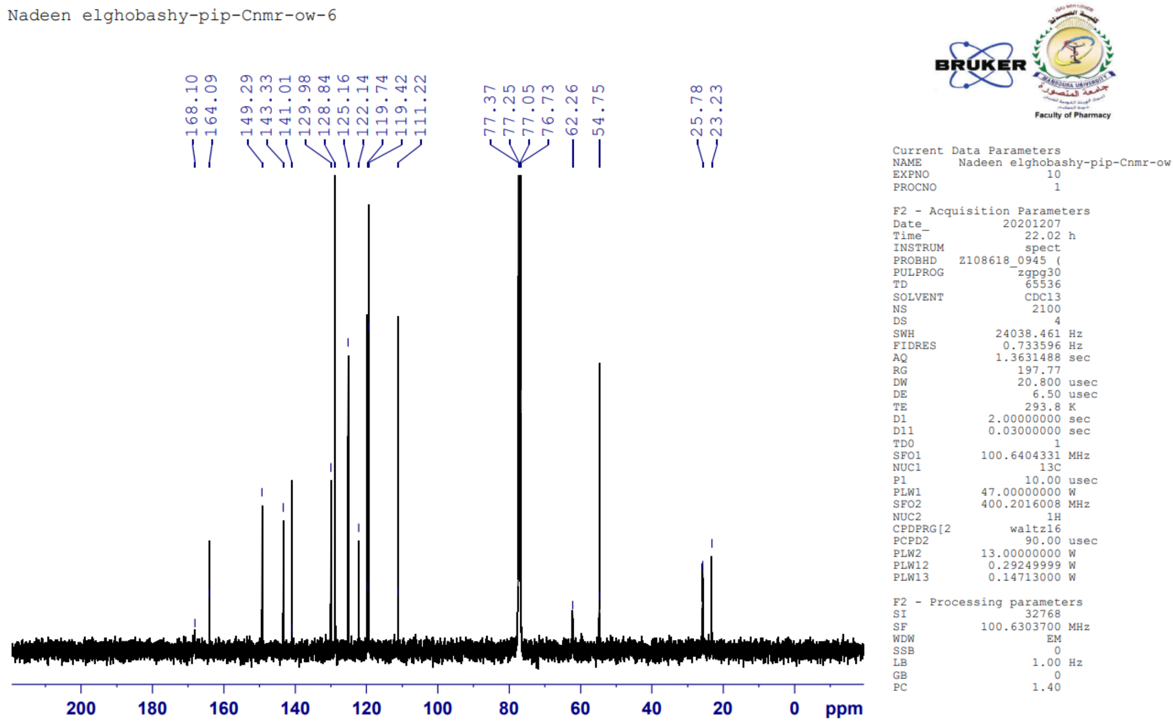

^{1}H NMR and ^{13}C NMR spectra of compound **7**

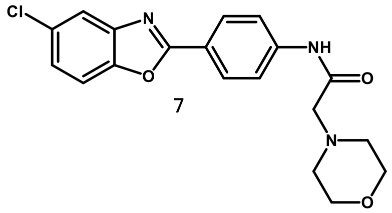

7

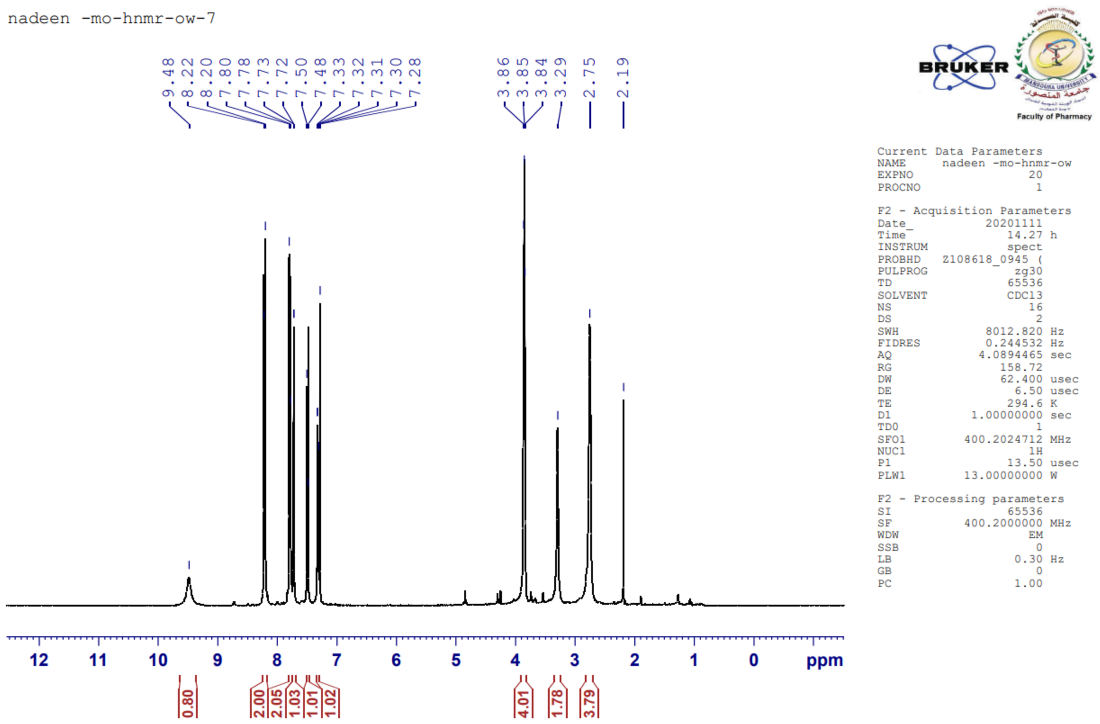

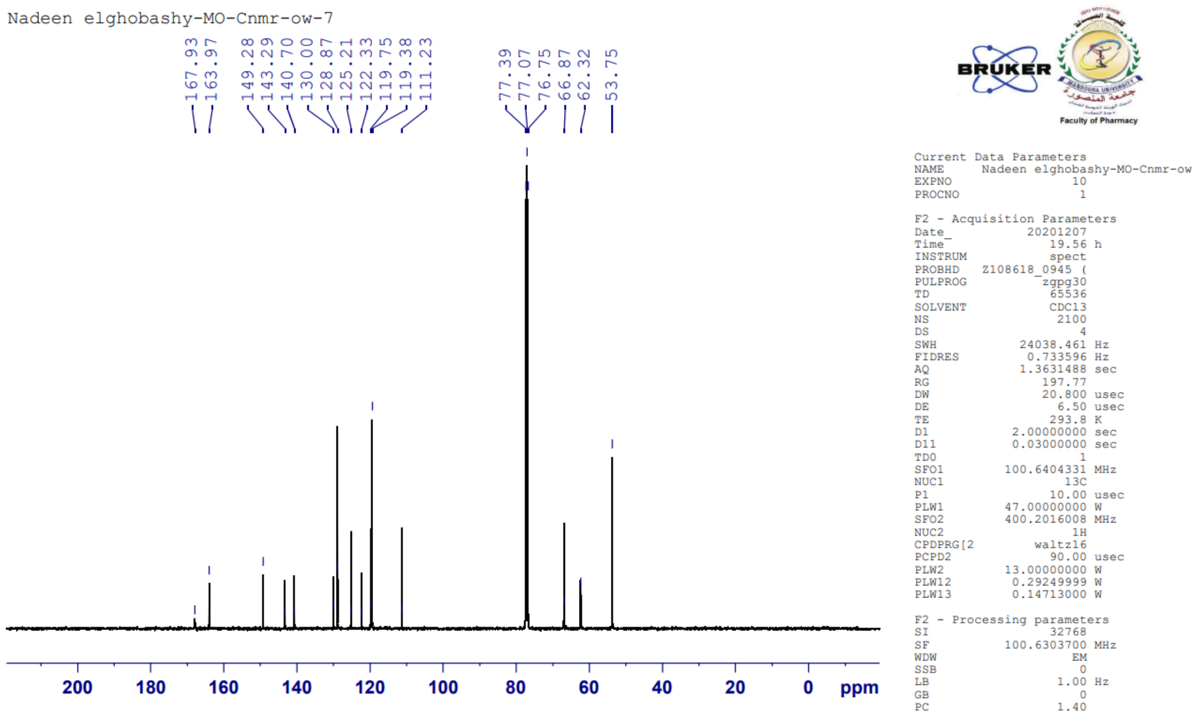

^{1}H NMR and ^{13}C NMR spectra of compound **8**

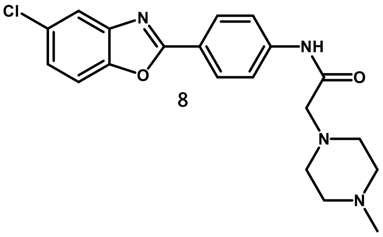

8

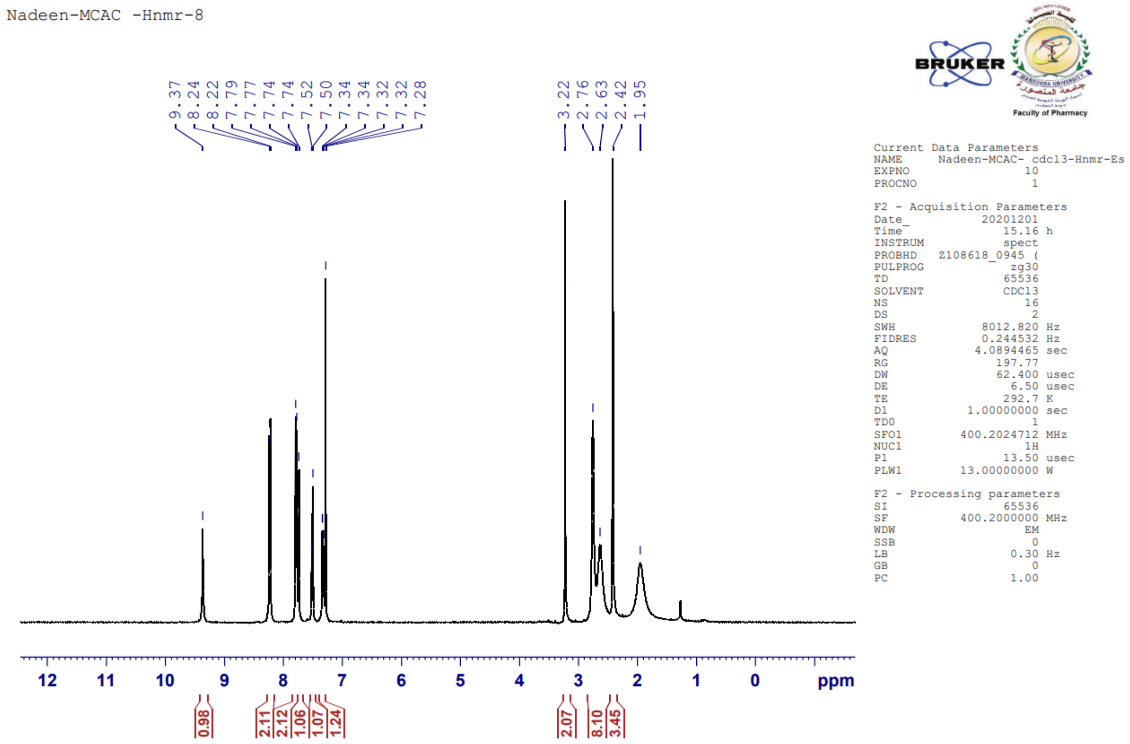

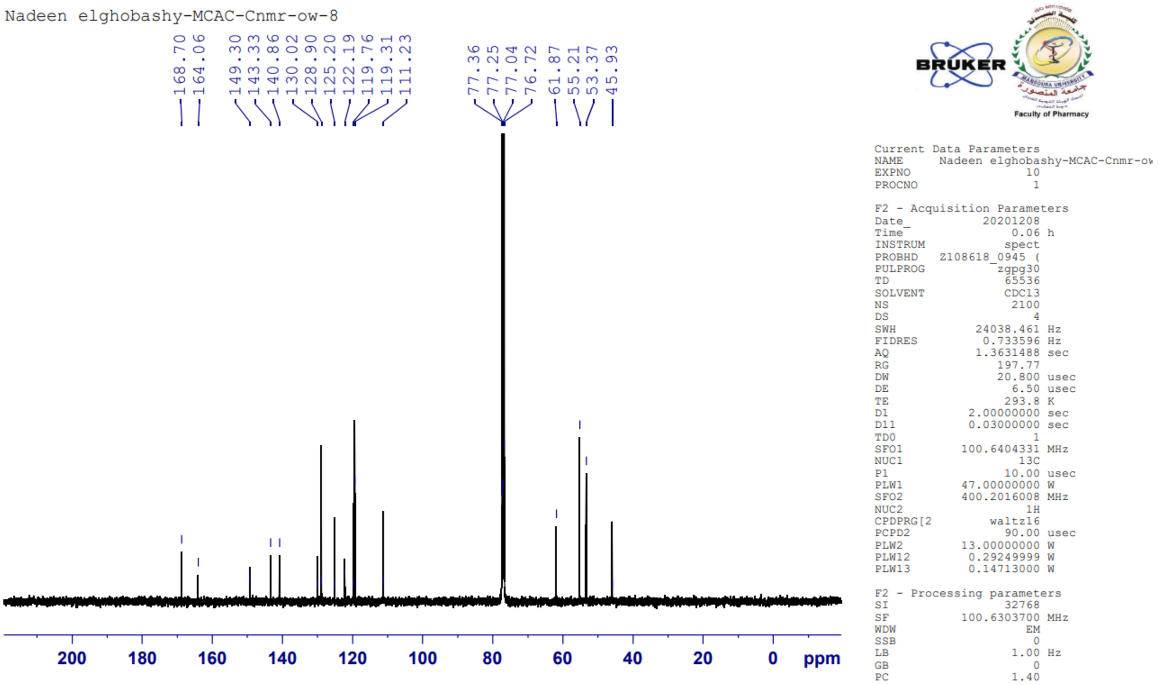

^{1}H NMR and ^{13}C NMR spectra of compound **9**

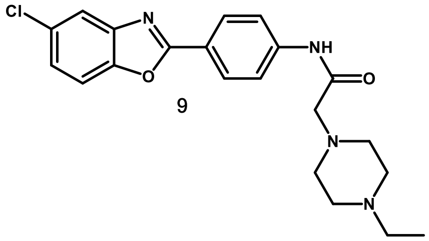

9

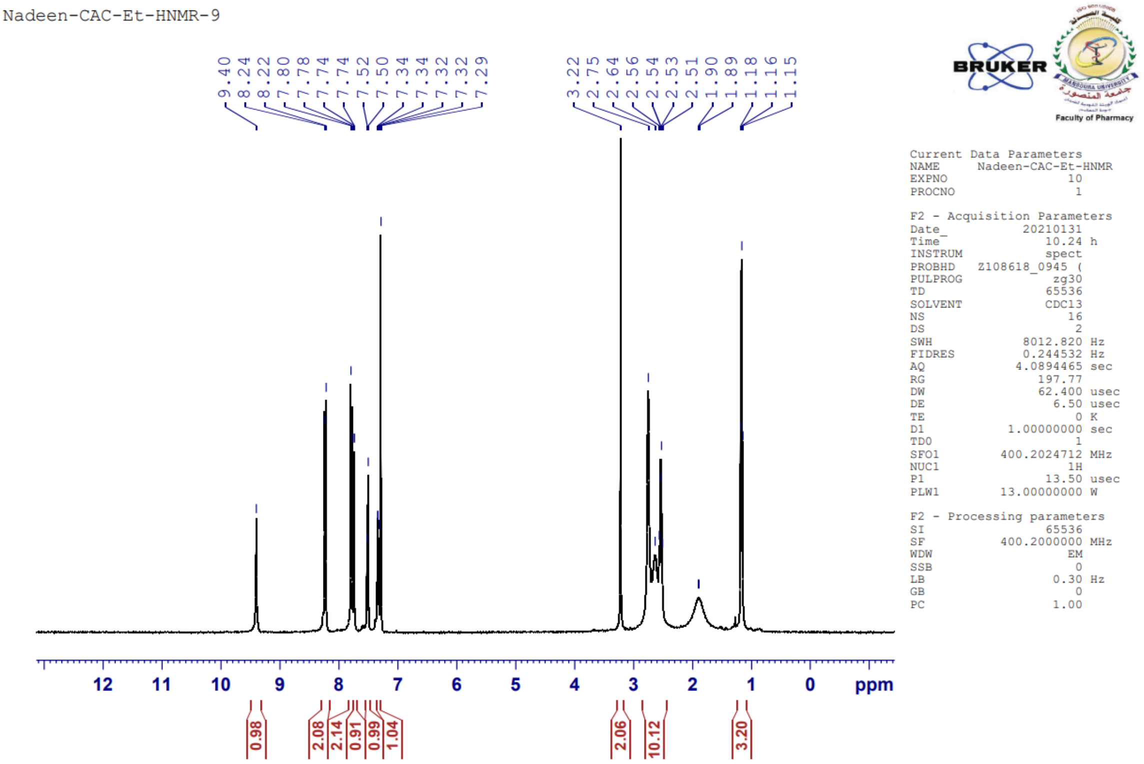

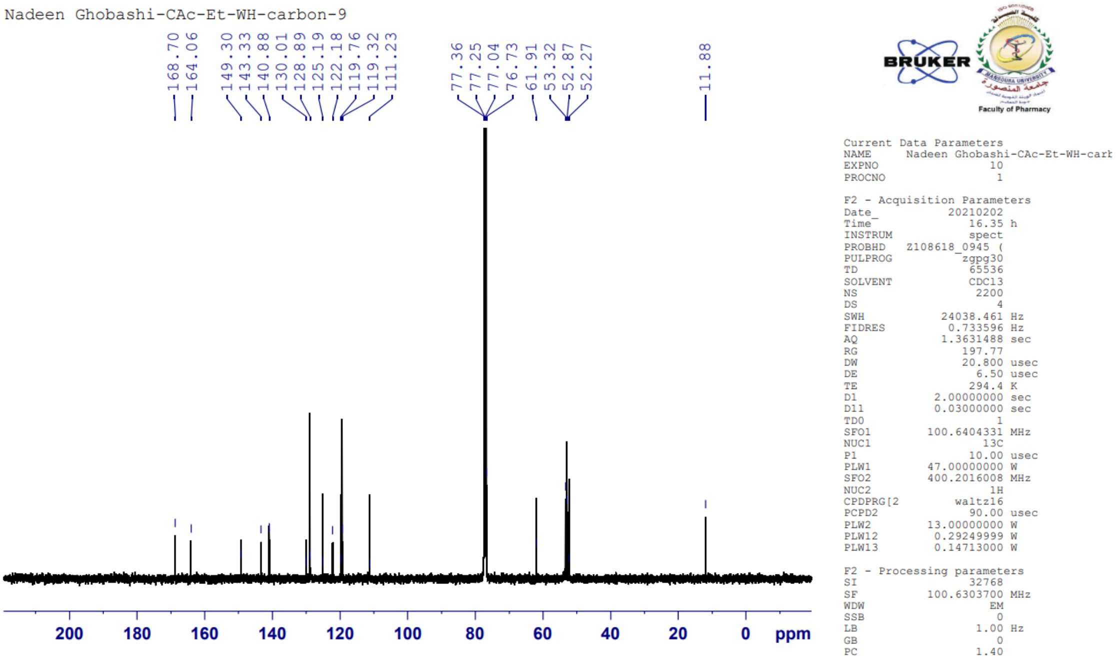

^{1}H NMR and ^{13}C NMR spectra of compound **10**

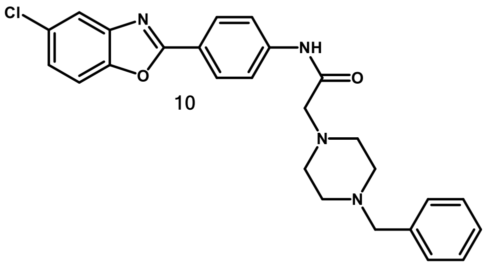

10

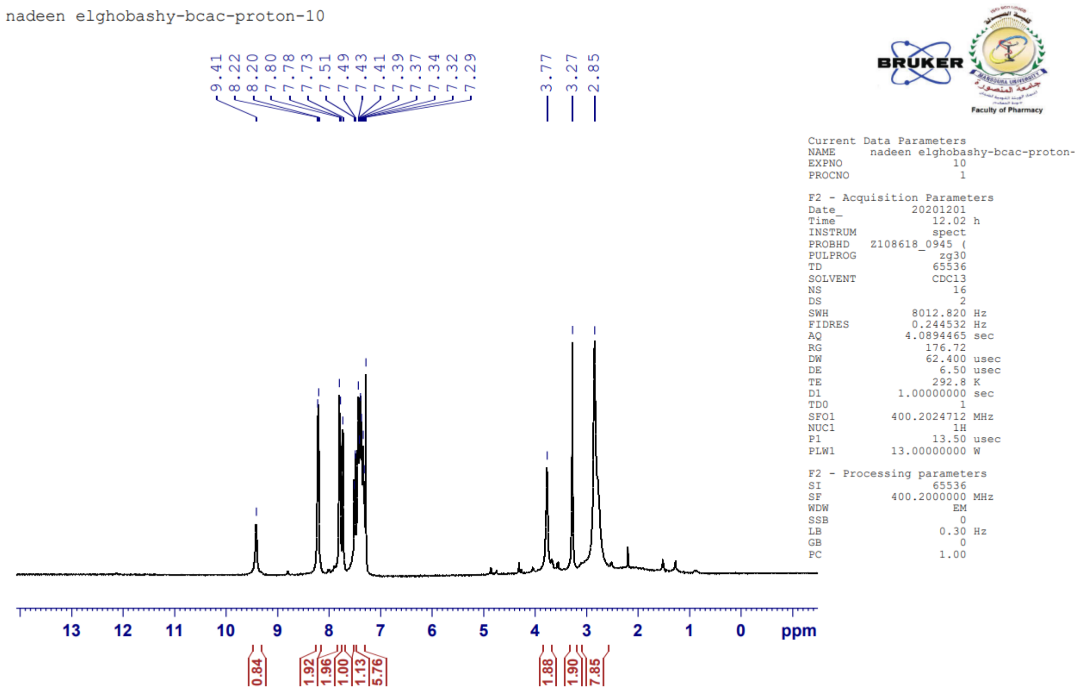

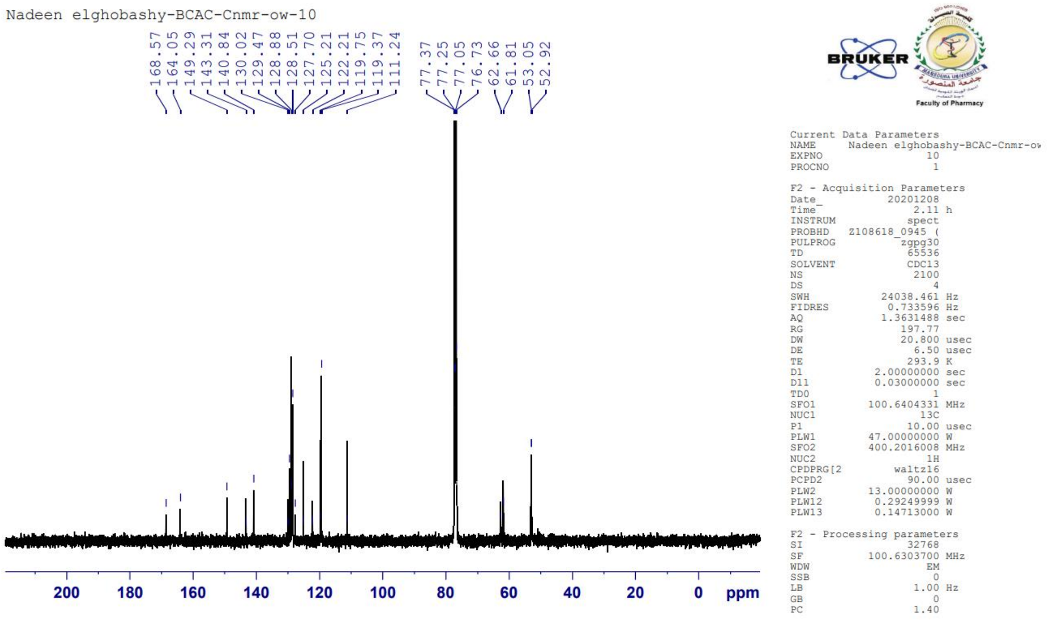

^{1}H NMR and ^{13}C NMR spectra of compound **11**

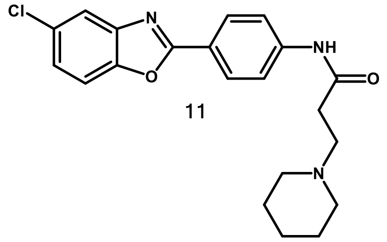

11

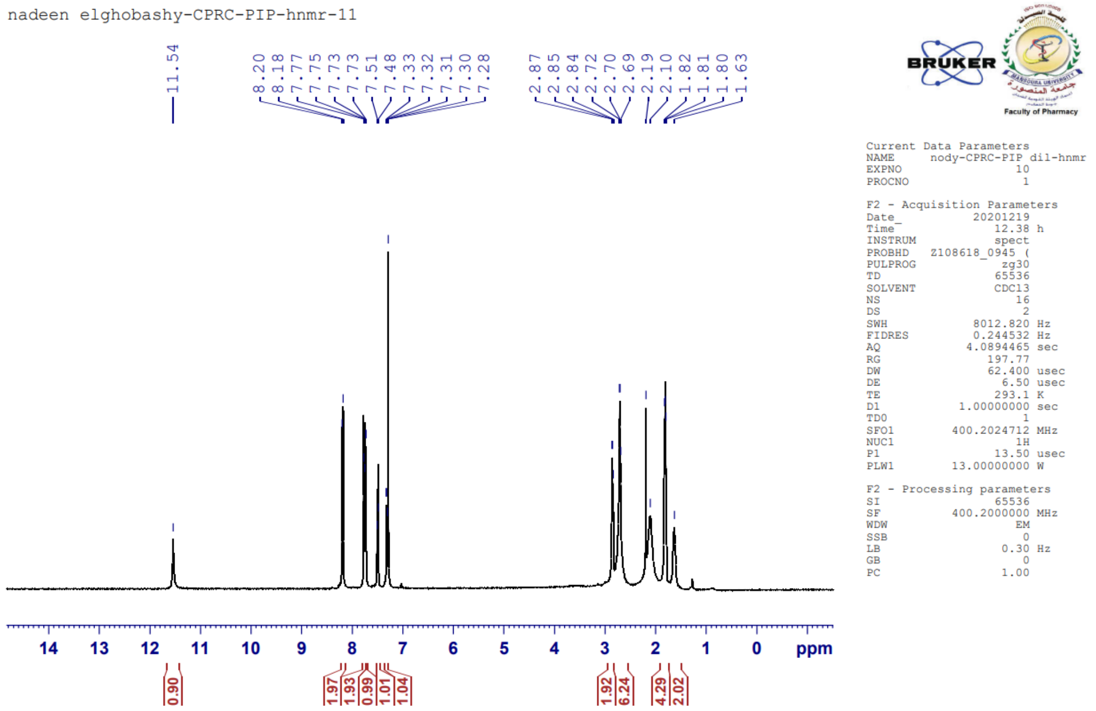

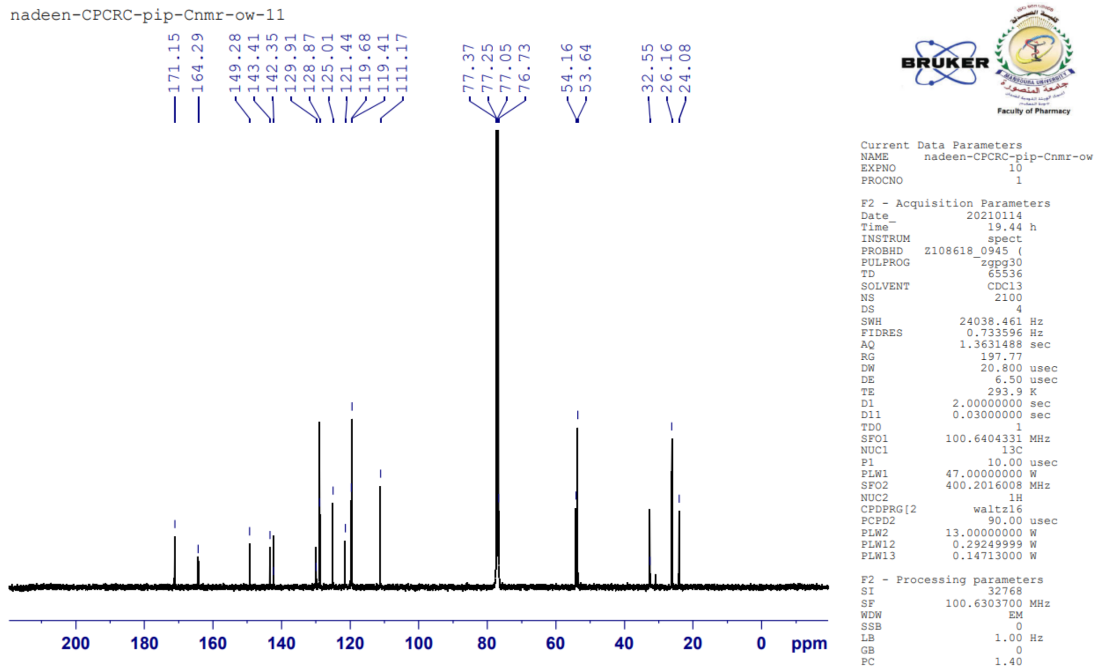

^{1}H NMR and ^{13}C NMR spectra of compound **12**

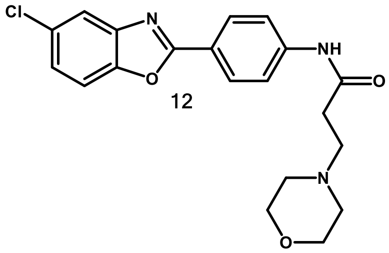

12

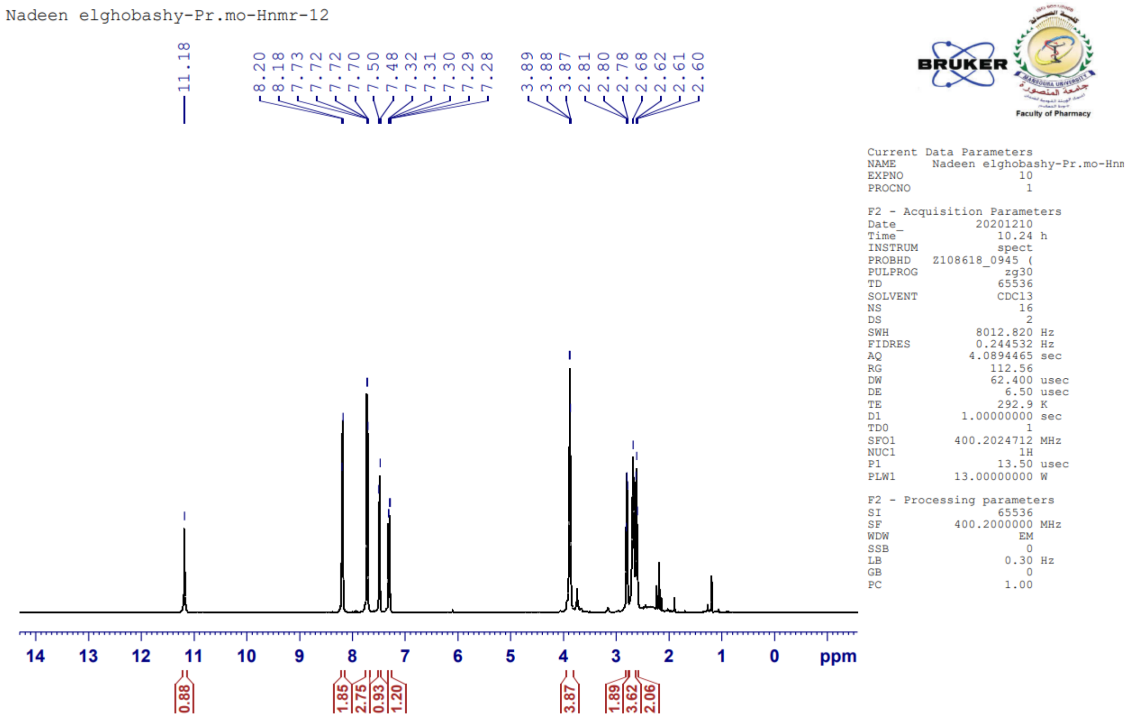

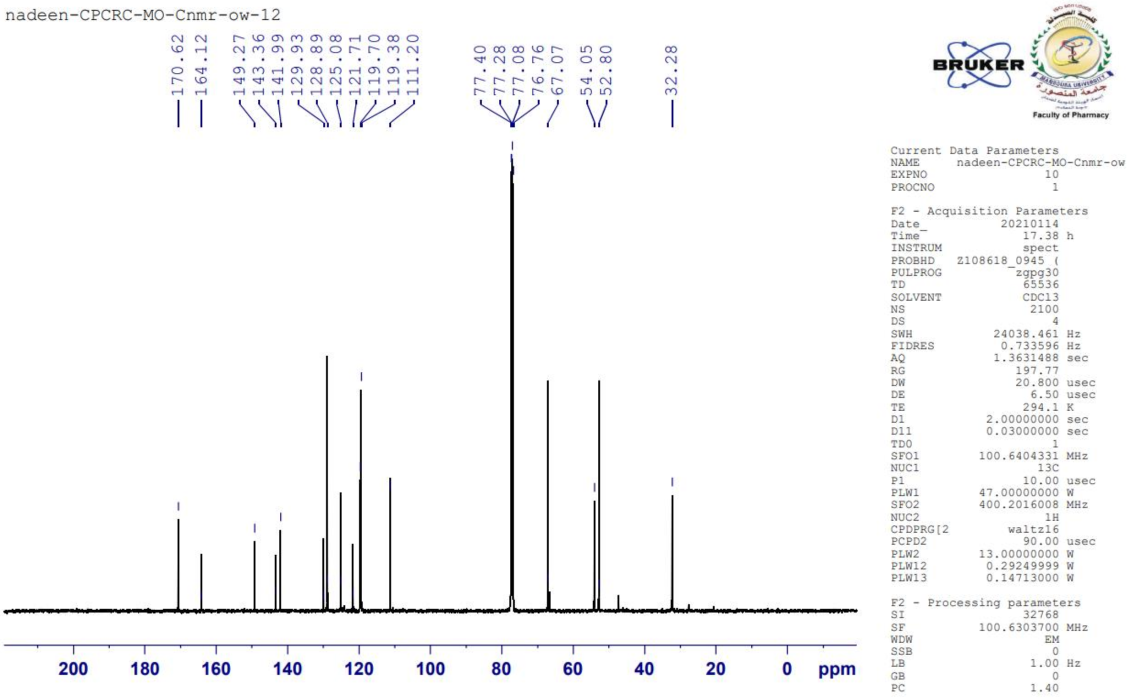

^{1}H NMR and ^{13}C NMR spectra of compound **13**

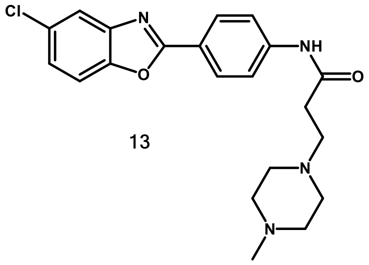

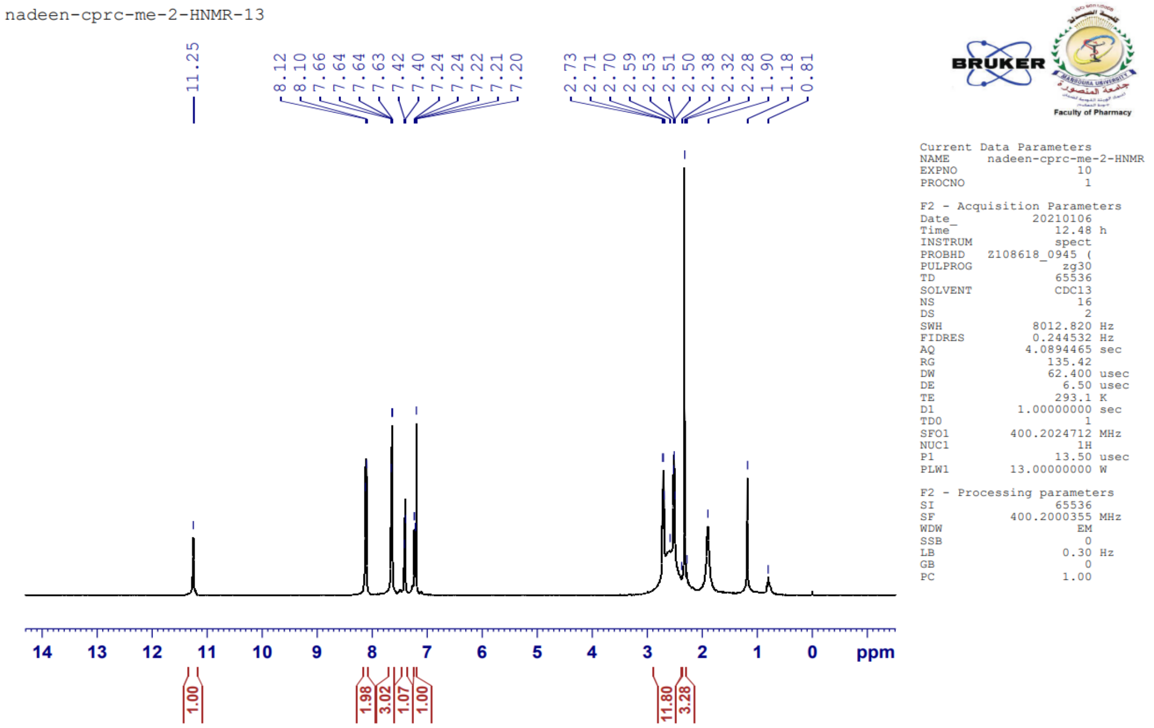

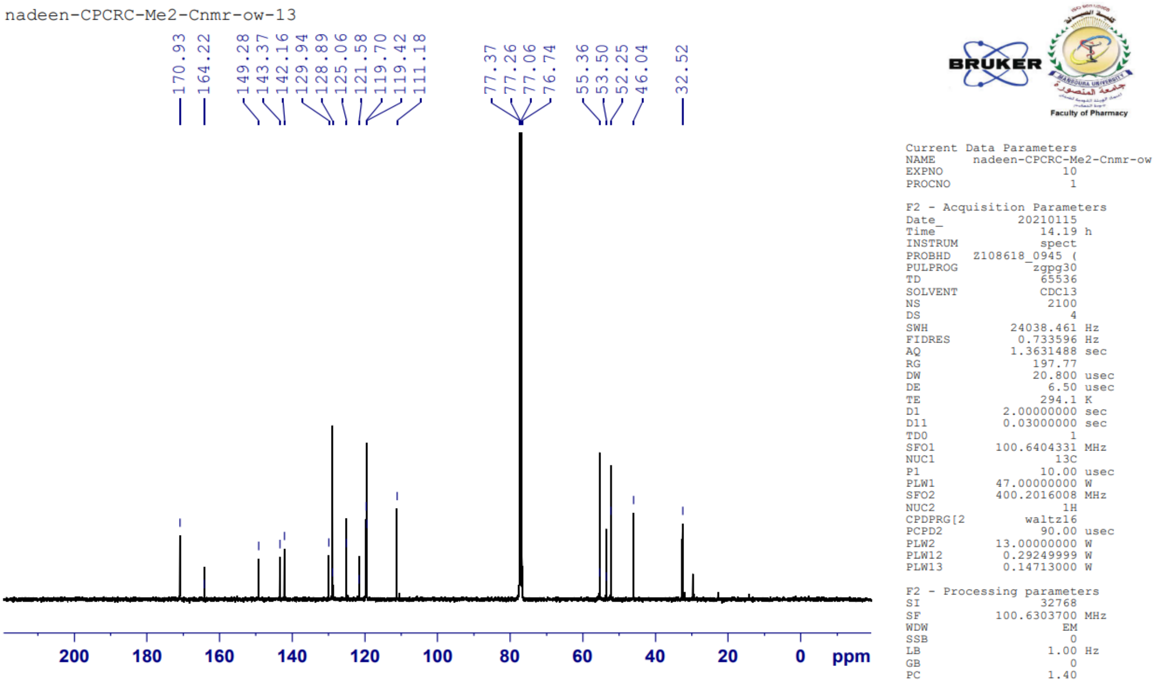

^{1}H NMR and ^{13}C NMR spectra of compound **14**

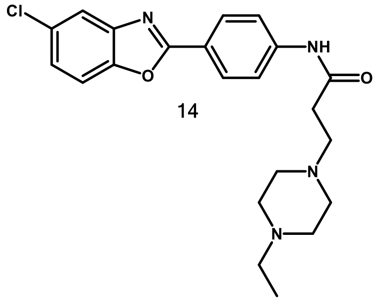

14

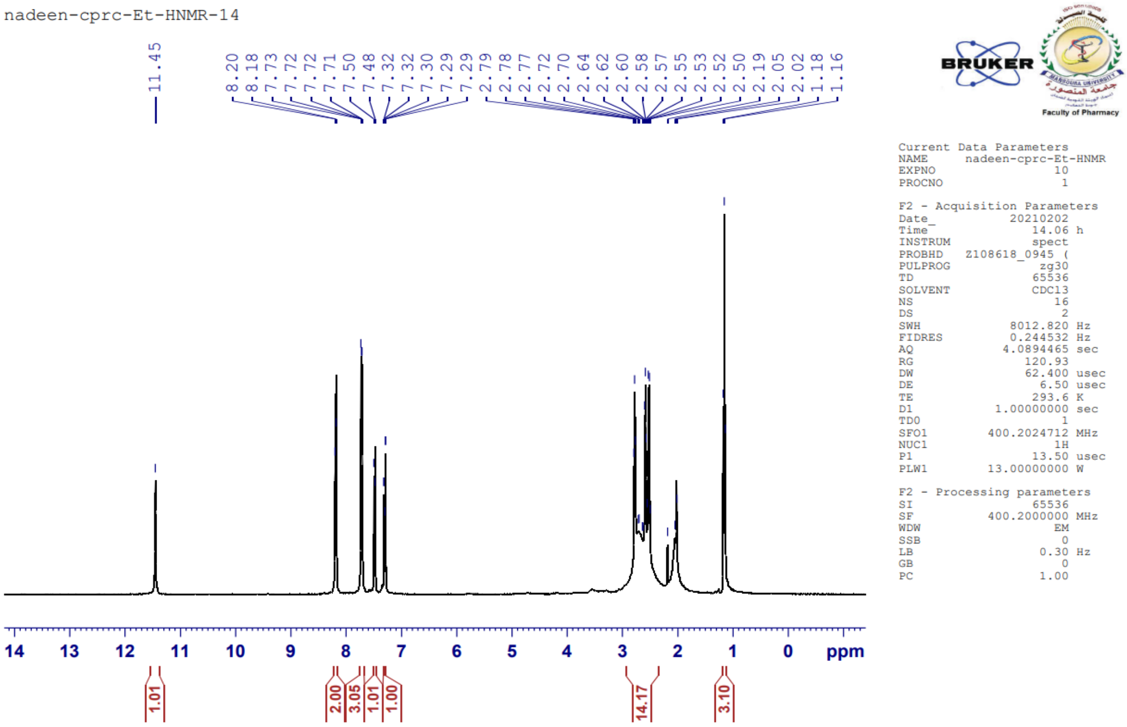

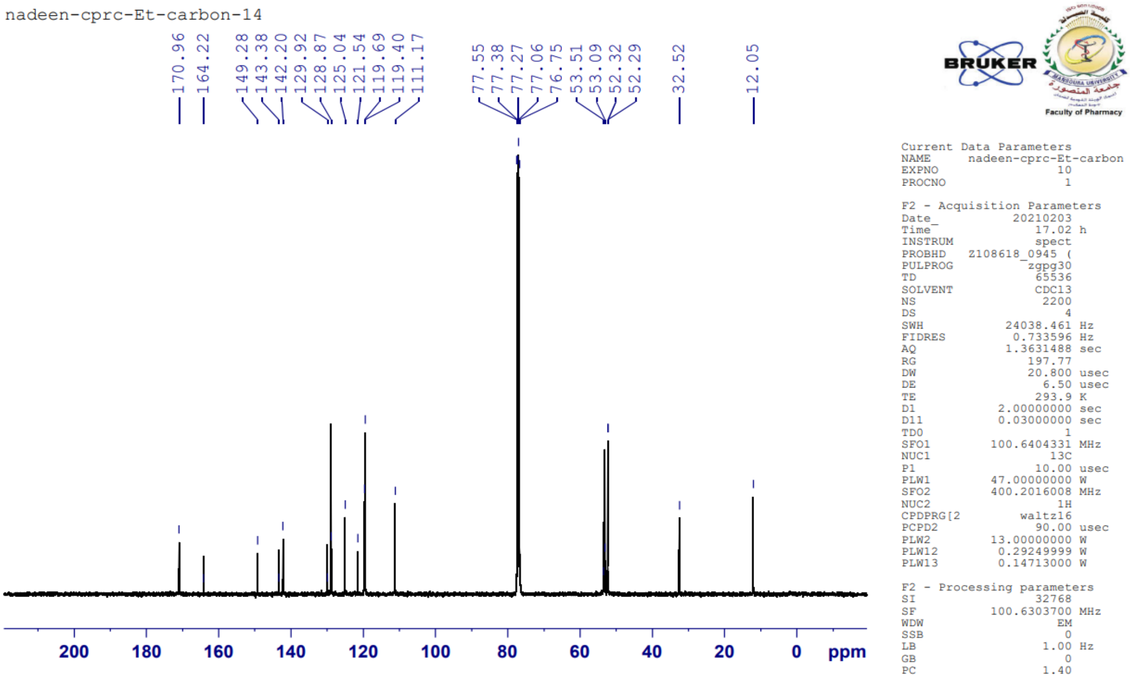

^{1}H NMR and ^{13}C NMR spectra of compound **15**

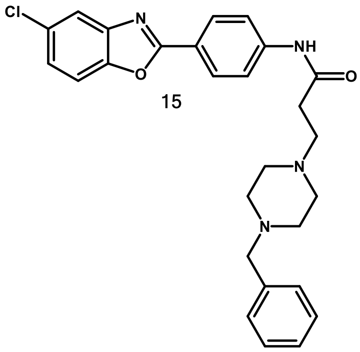

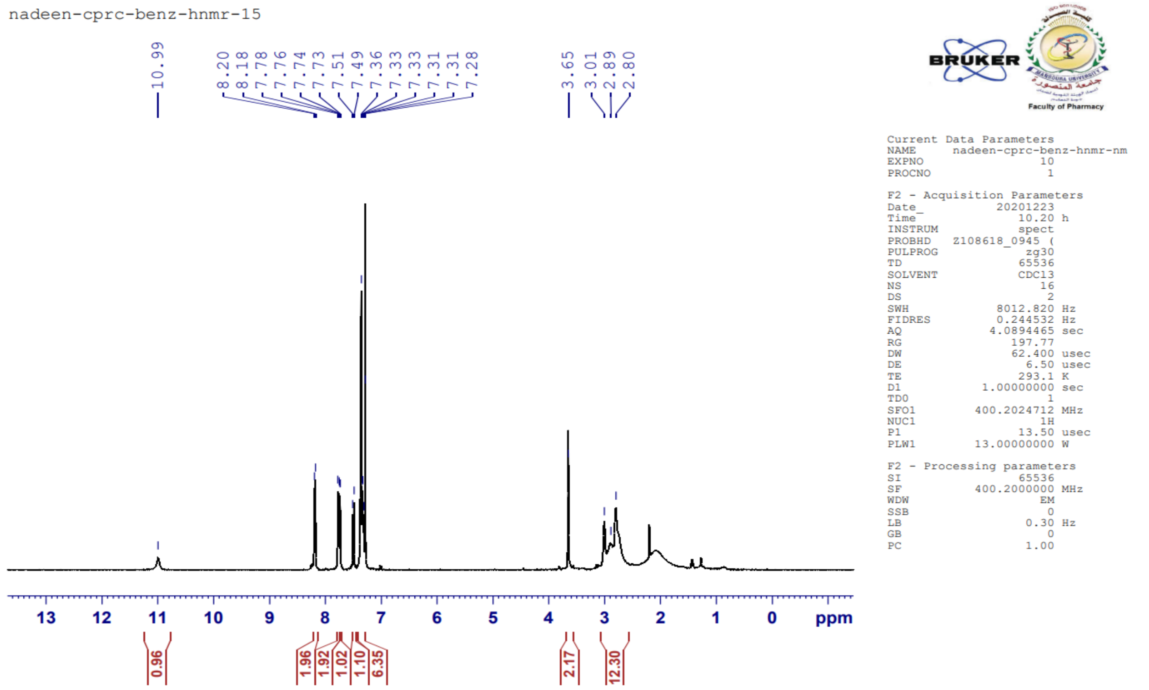

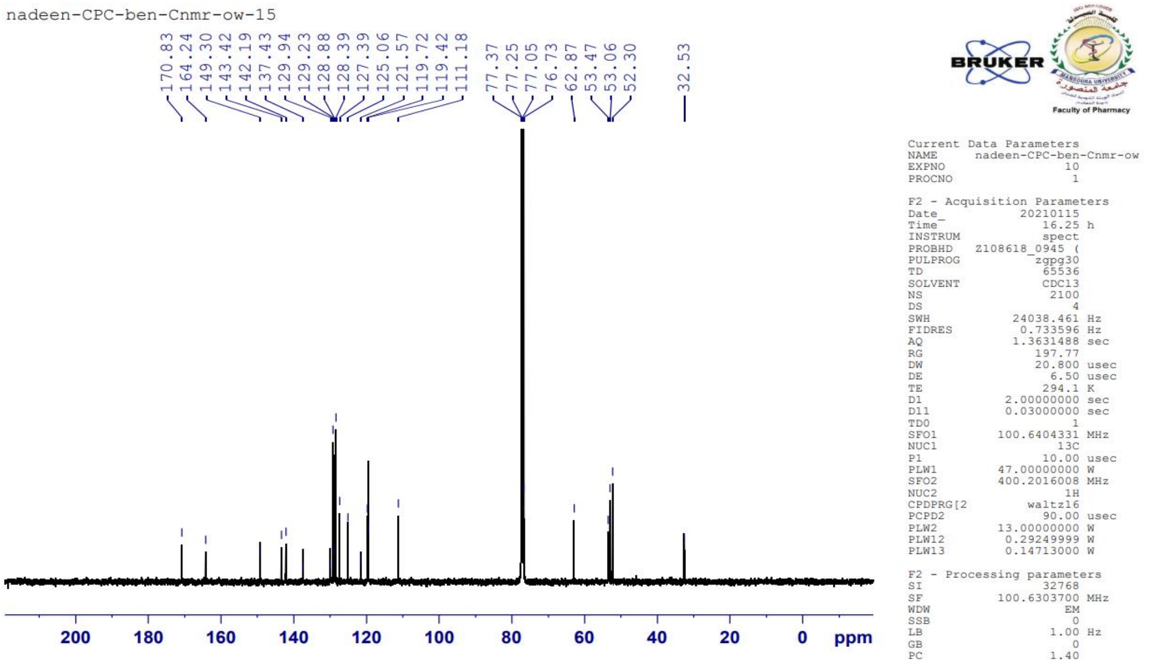

^{1}H NMR and ^{13}C NMR spectra of compound **16**

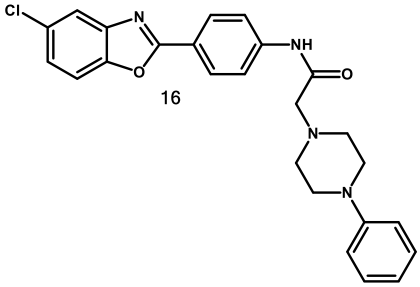

16

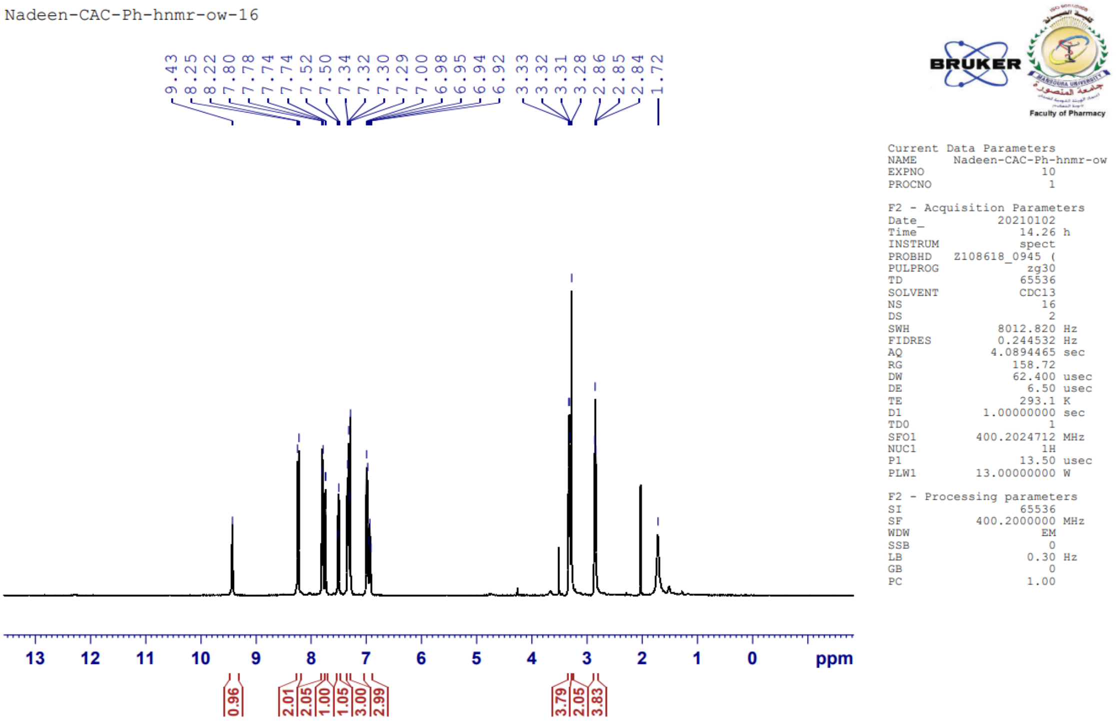

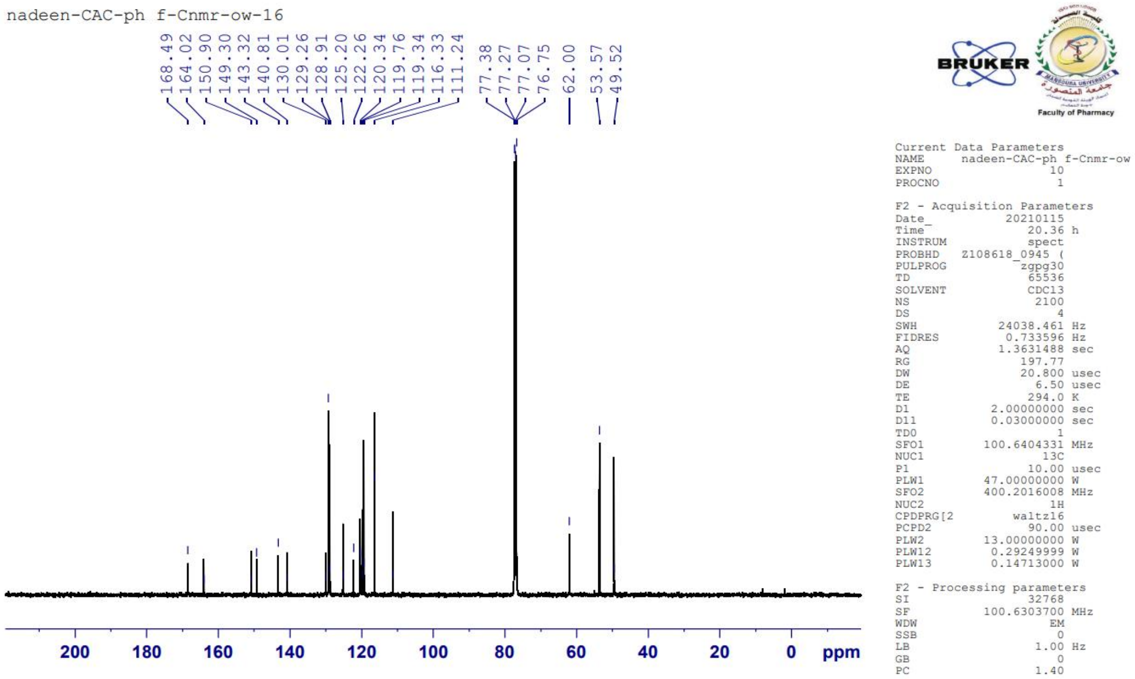

^{1}H NMR and ^{13}C NMR spectra of compound **17**

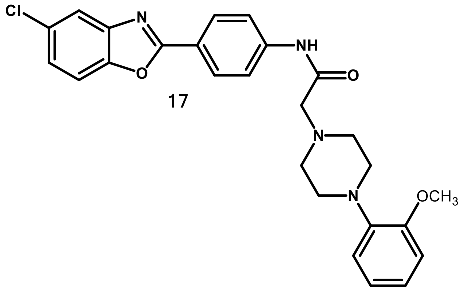

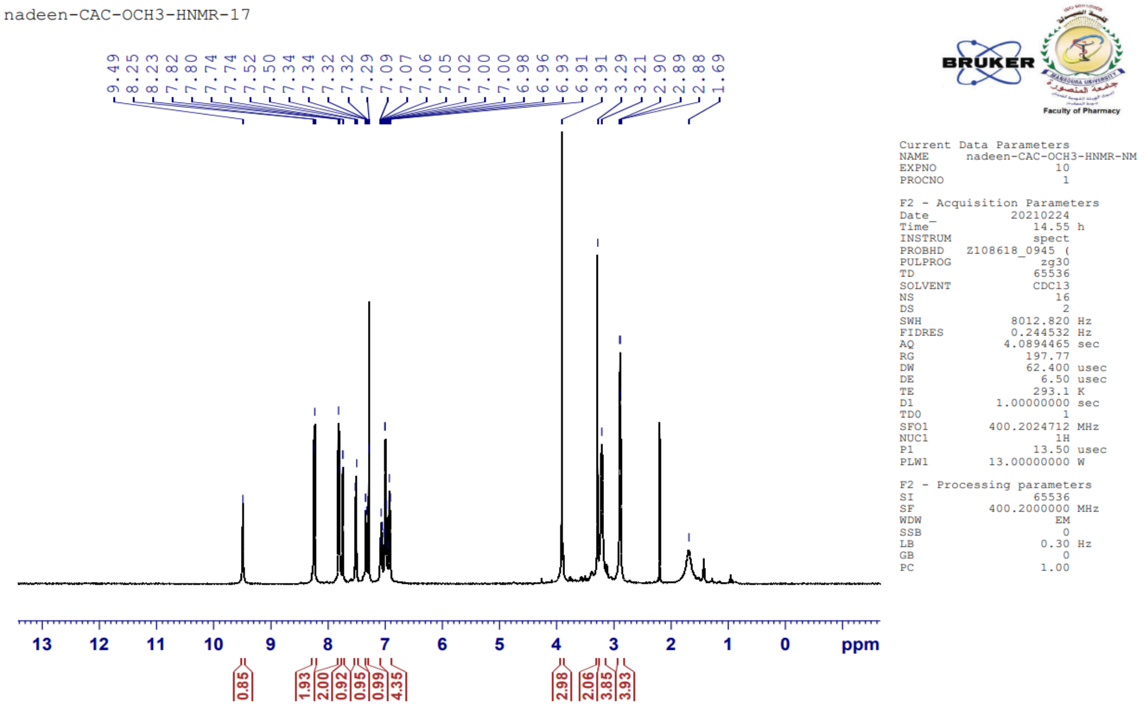

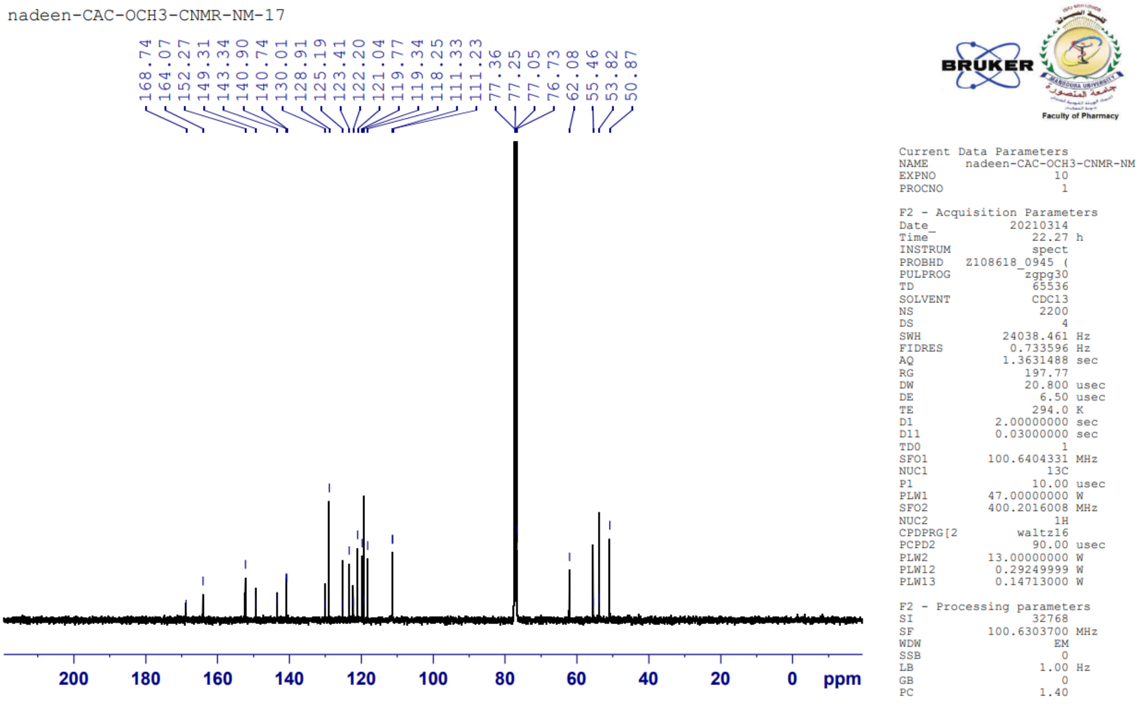

^{1}H NMR and ^{13}C NMR spectra of compound **18**

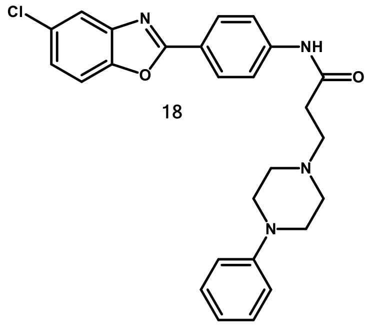

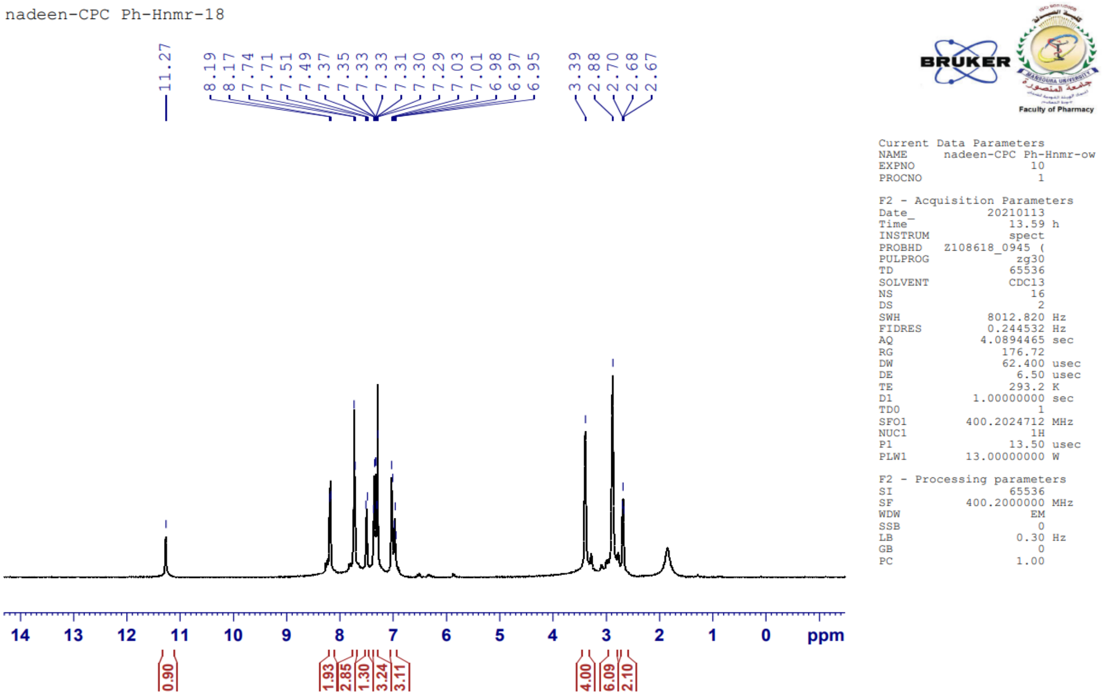

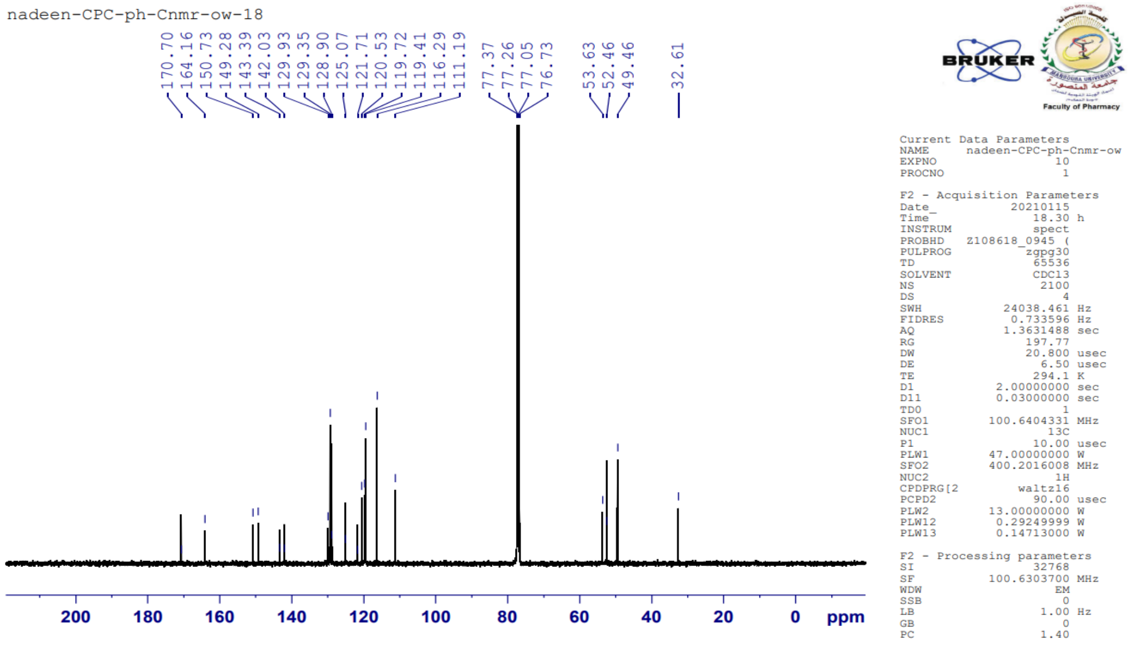

^{1}H NMR and ^{13}C NMR spectra of compound **19**

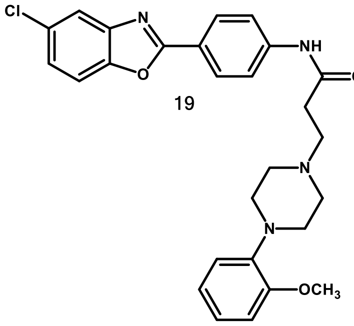

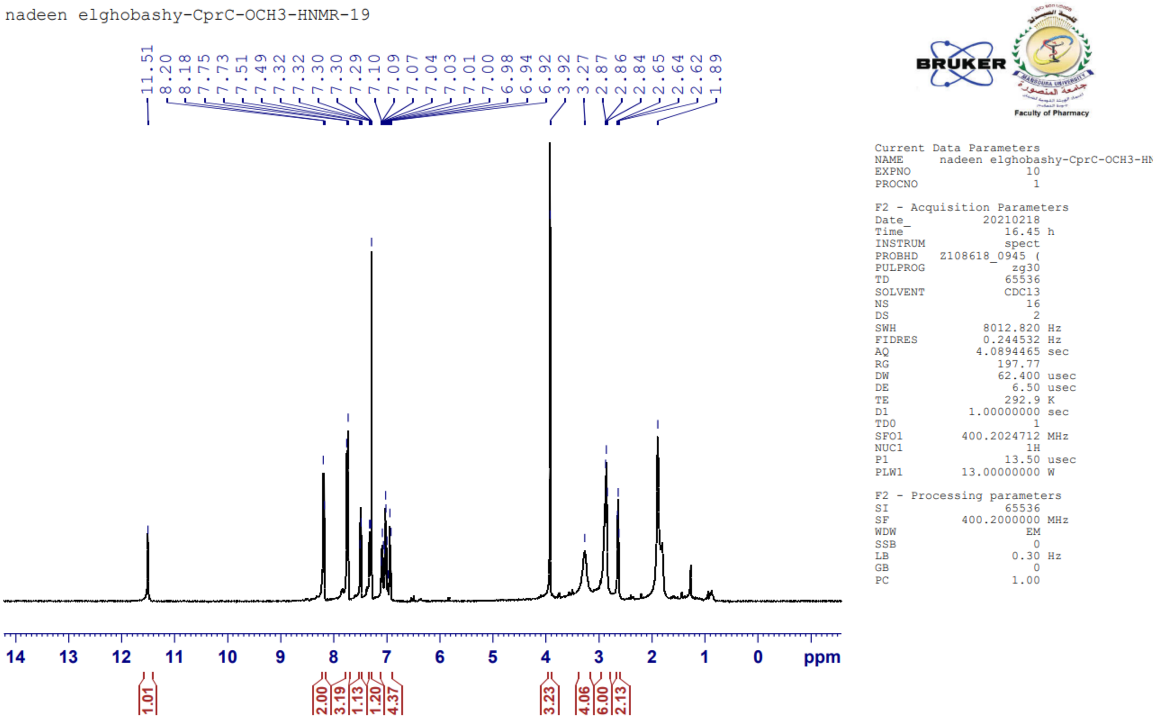

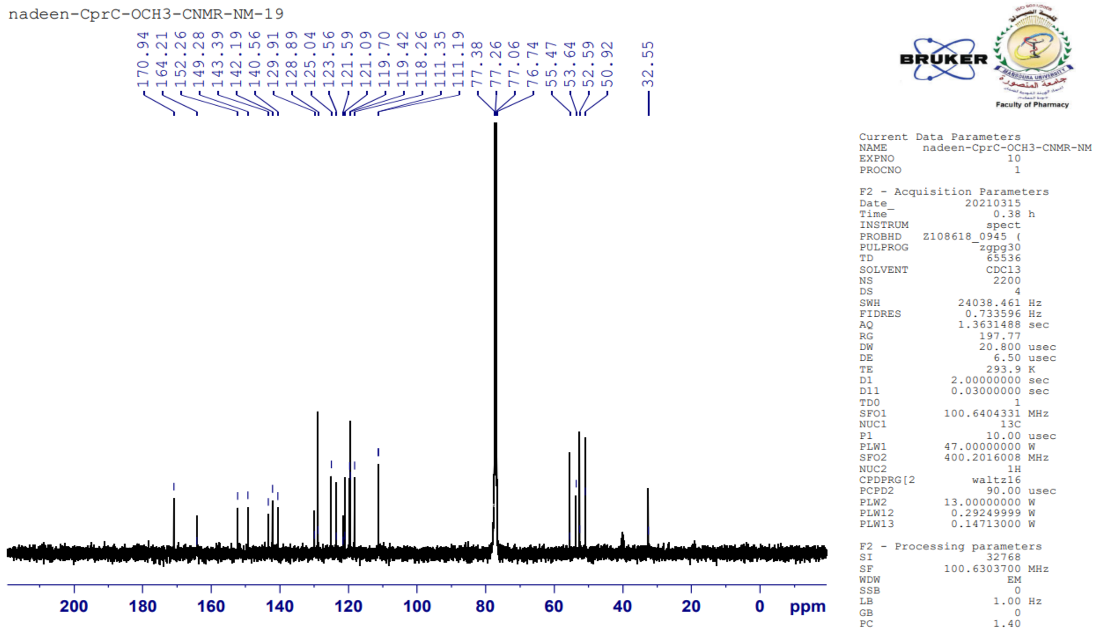

^{1}H NMR and ^{13}C NMR spectra of compound **20**

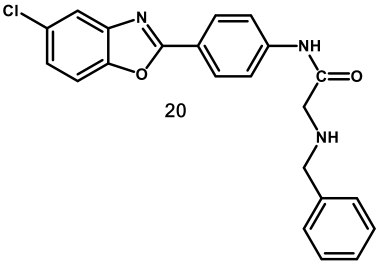

20

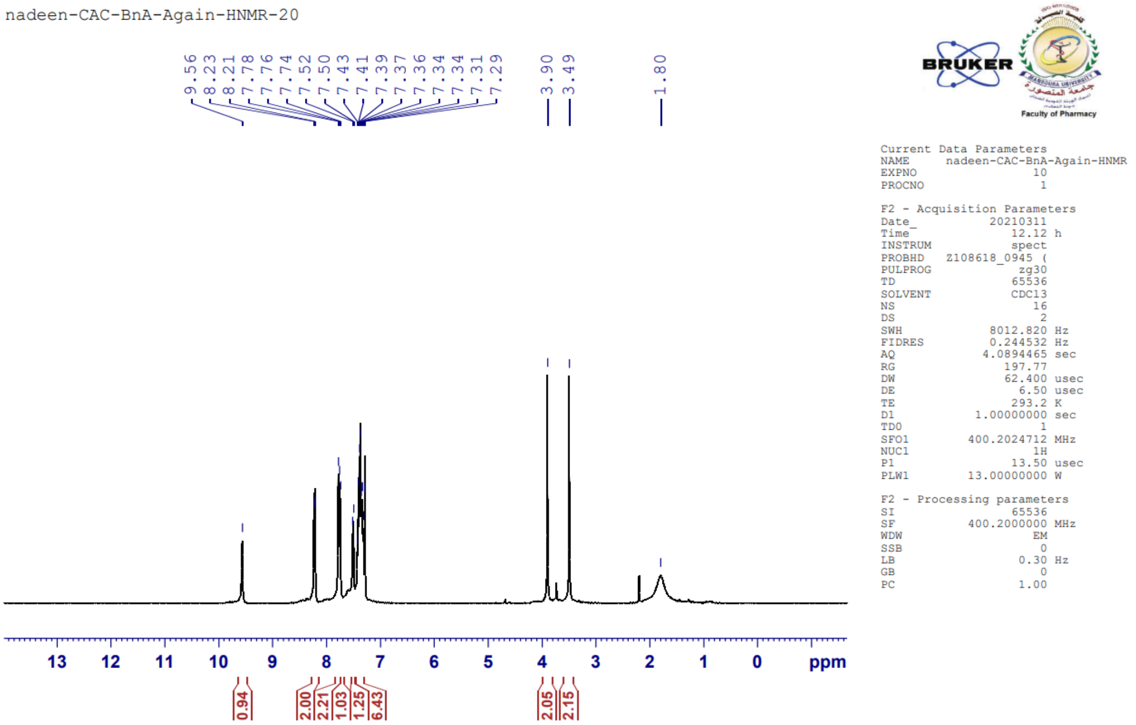

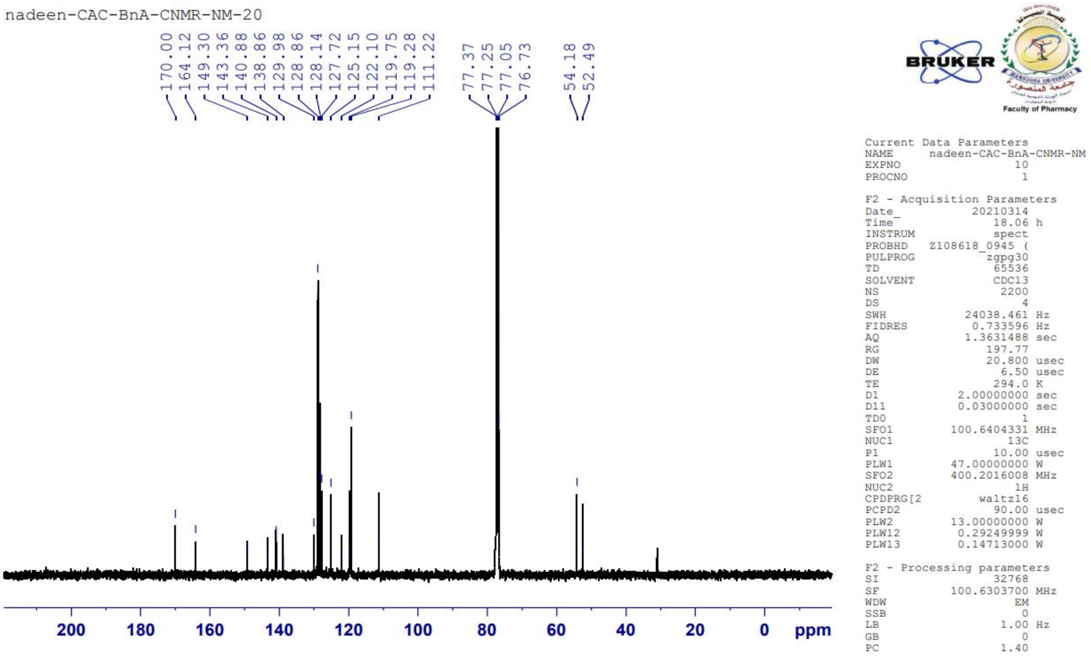

^{1}H NMR and ^{13}C NMR spectra of compound **21**

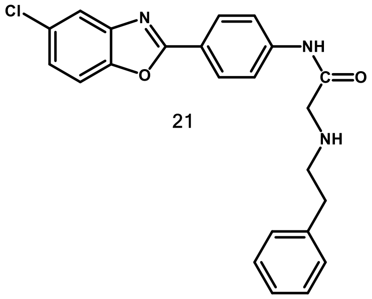

21

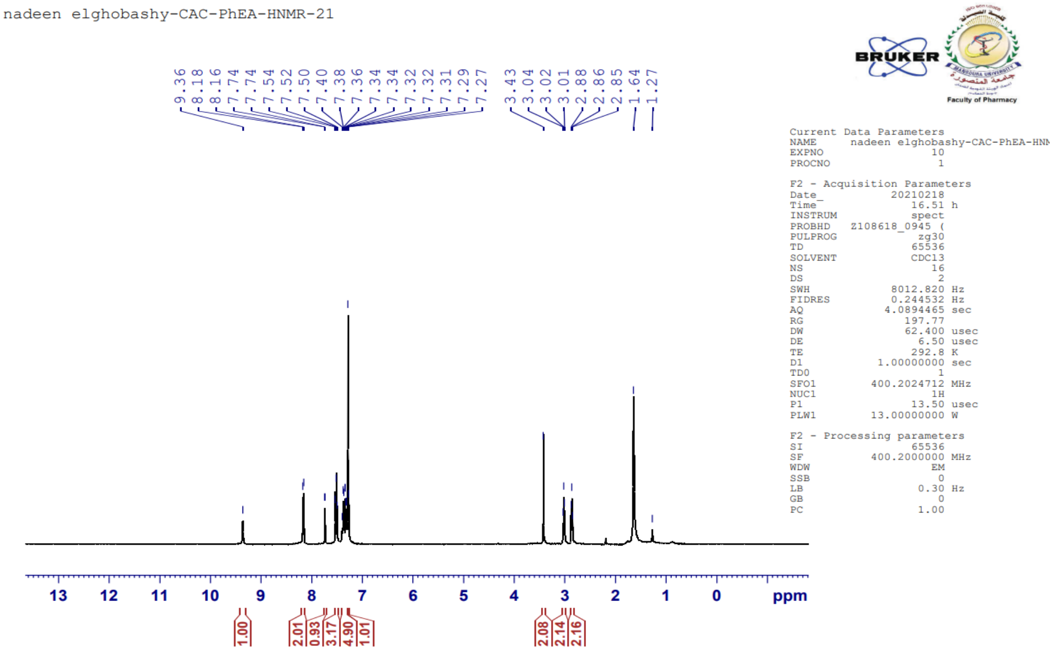

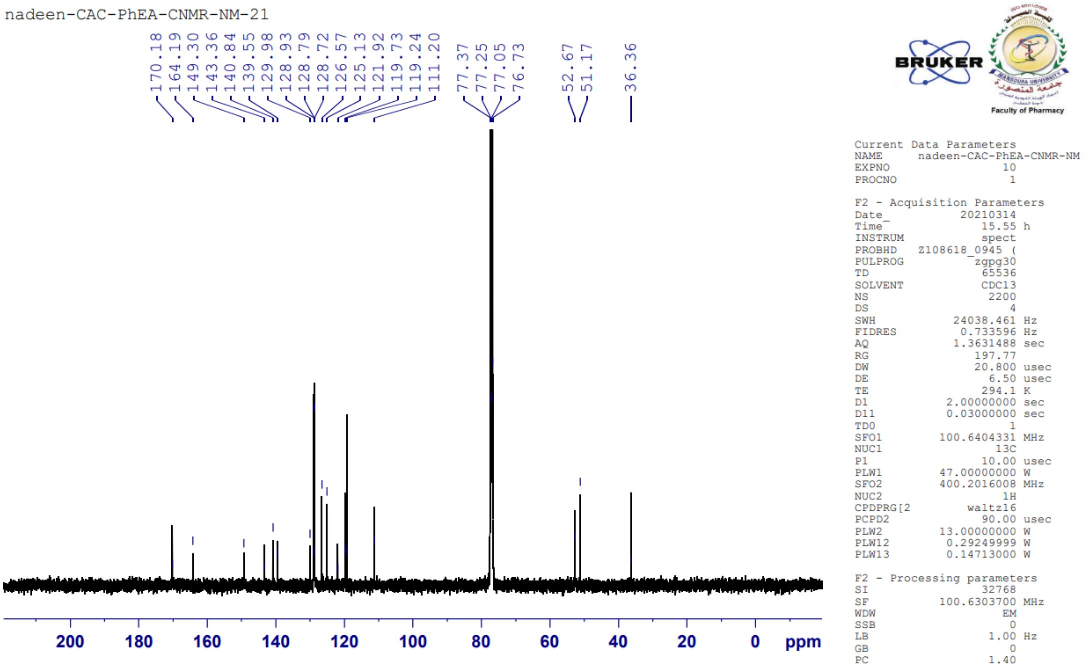

^{1}H NMR and ^{13}C NMR spectra of compound **22**

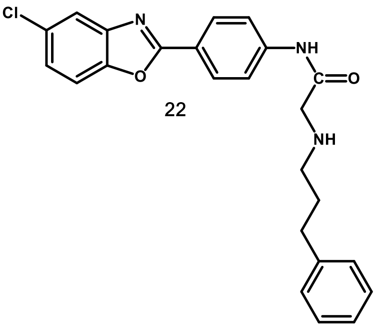

22

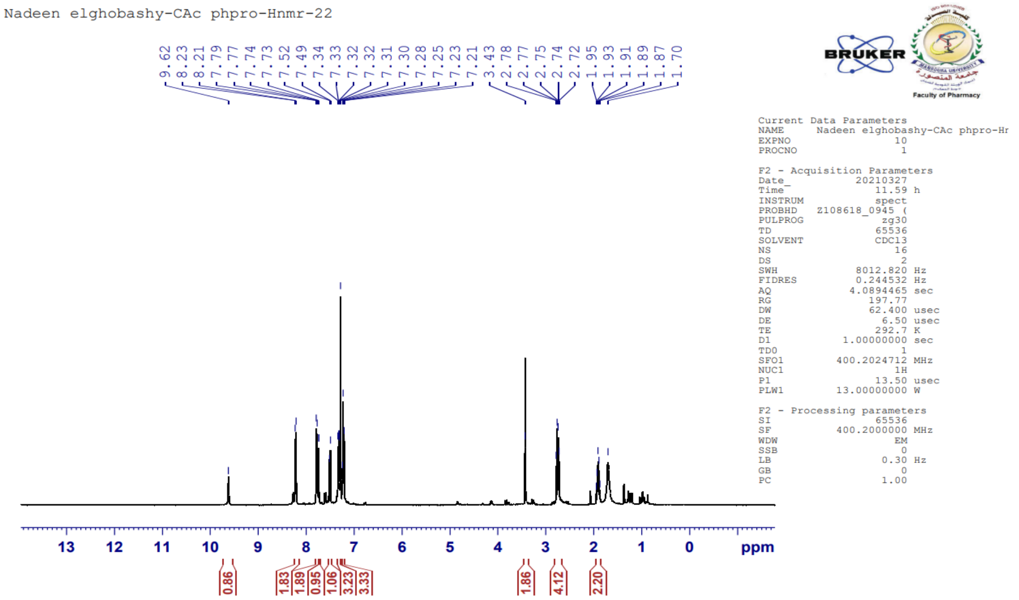

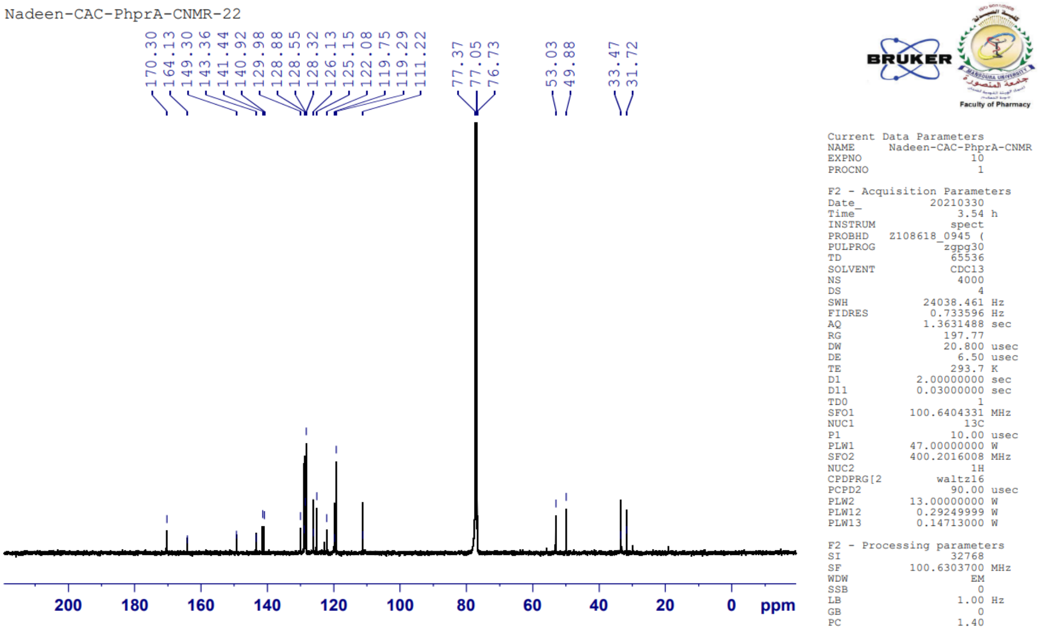

^1H NMR and ^{13}C NMR spectra of compound **23**

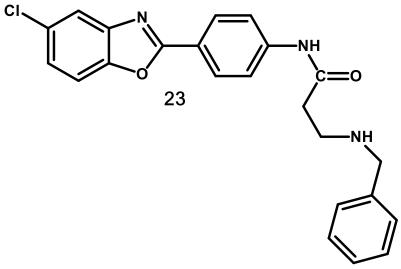

23

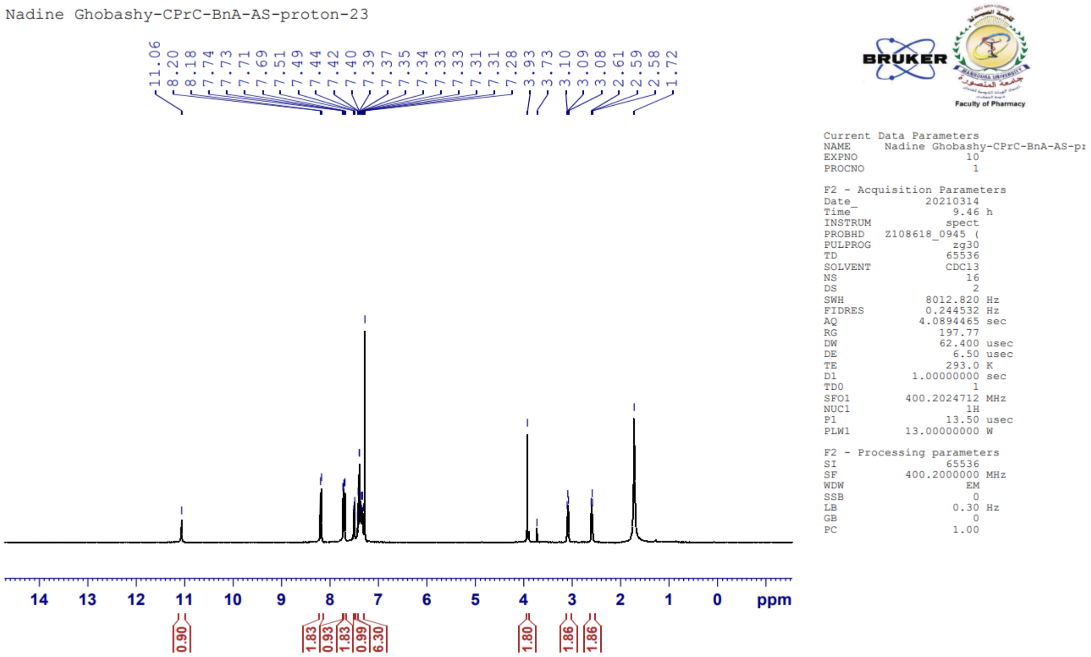

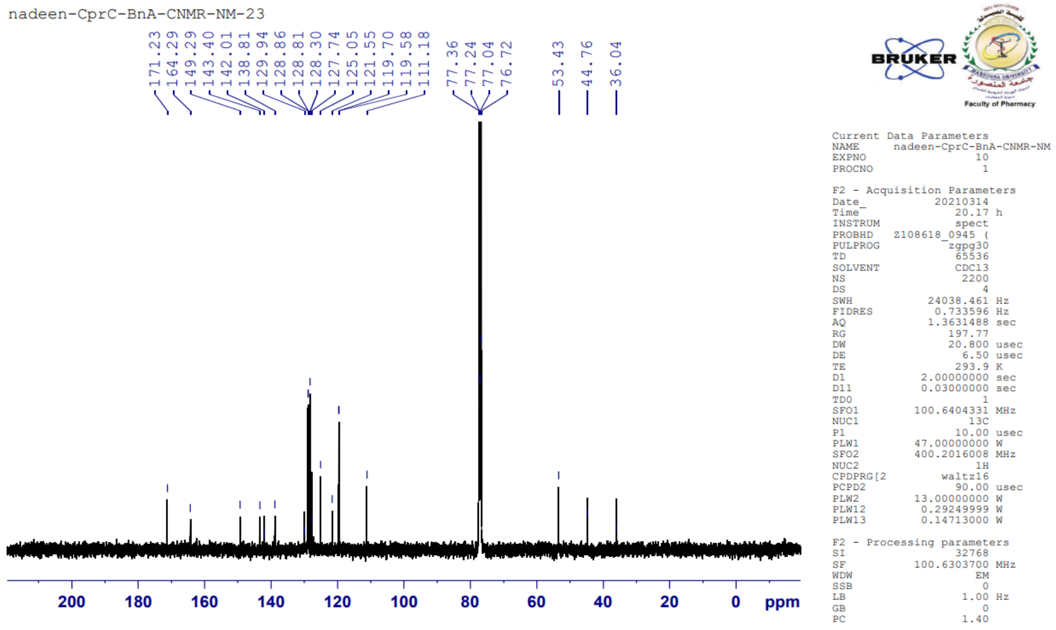

^{1}H NMR and ^{13}C NMR spectra of compound **24**

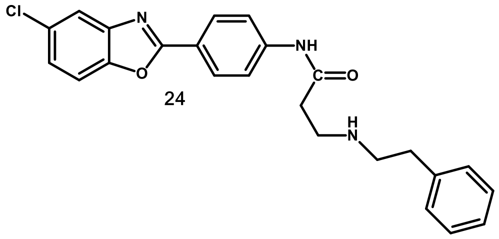

24

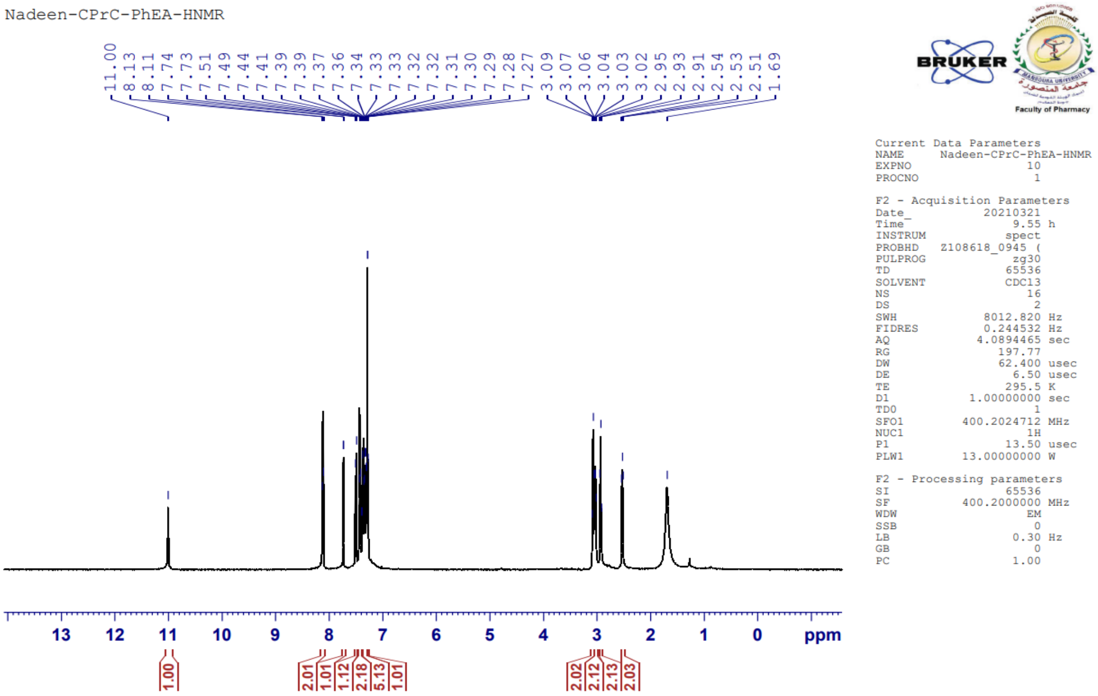

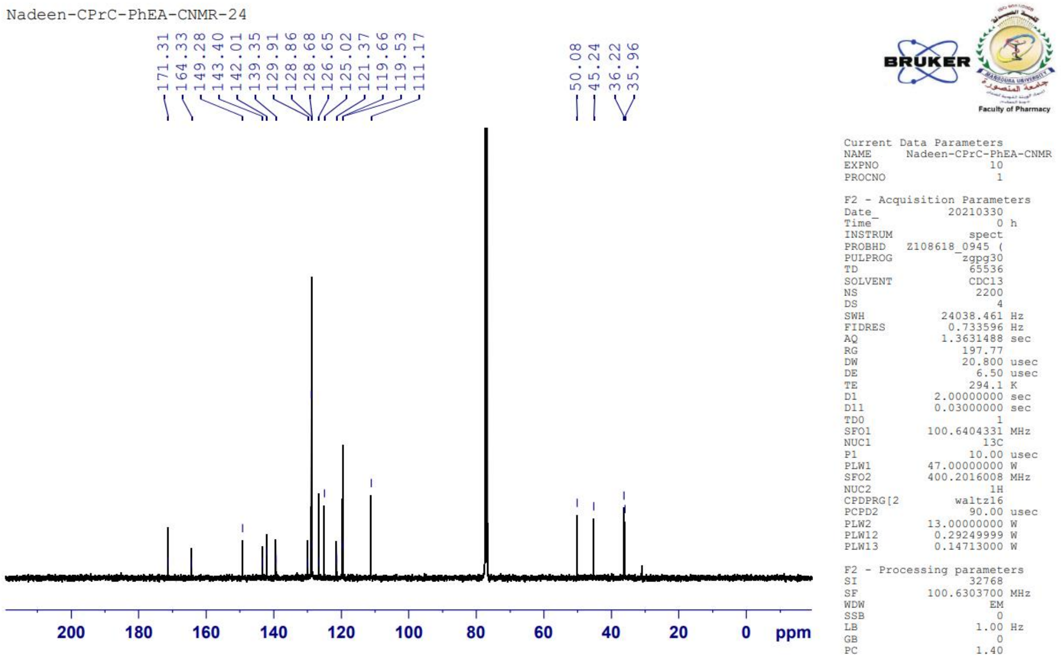

^{1}H NMR and ^{13}C NMR spectra of compound **25**

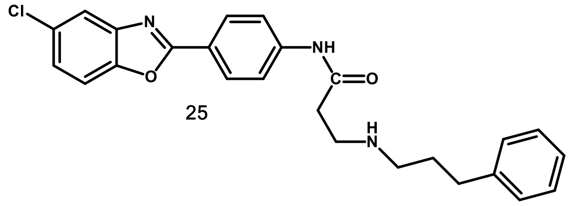

25

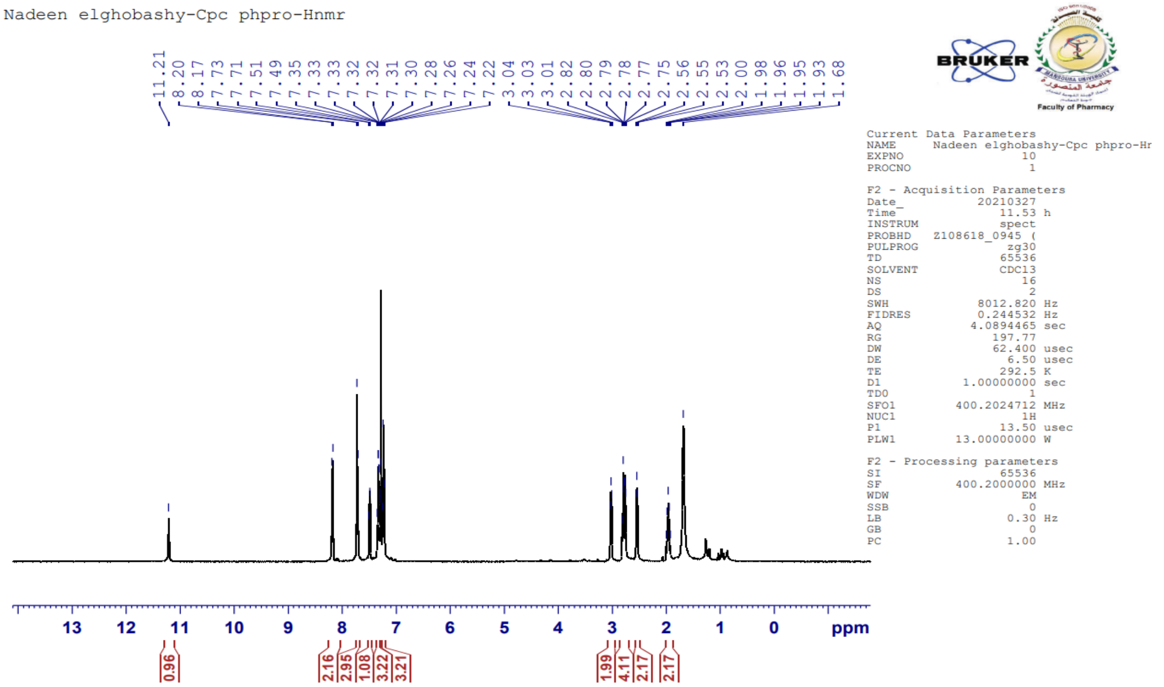

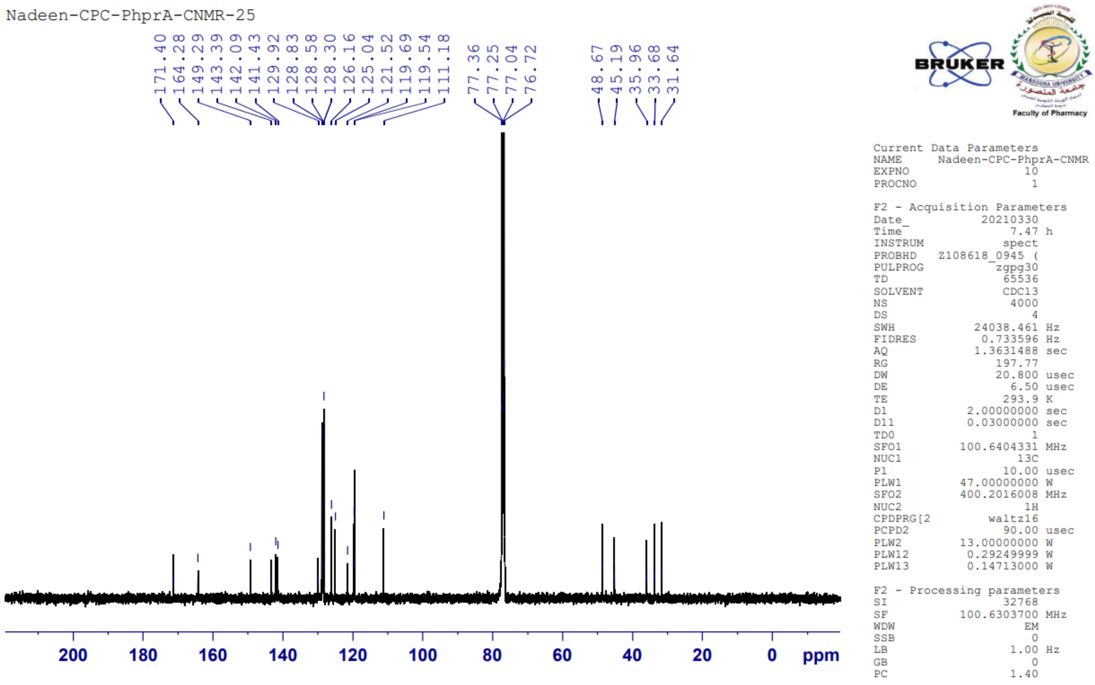

^{1}H NMR and ^{13}C NMR spectra of compound **26**

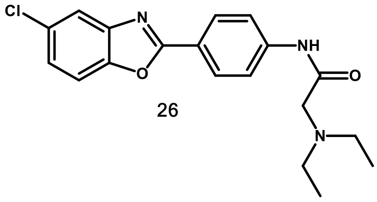

26

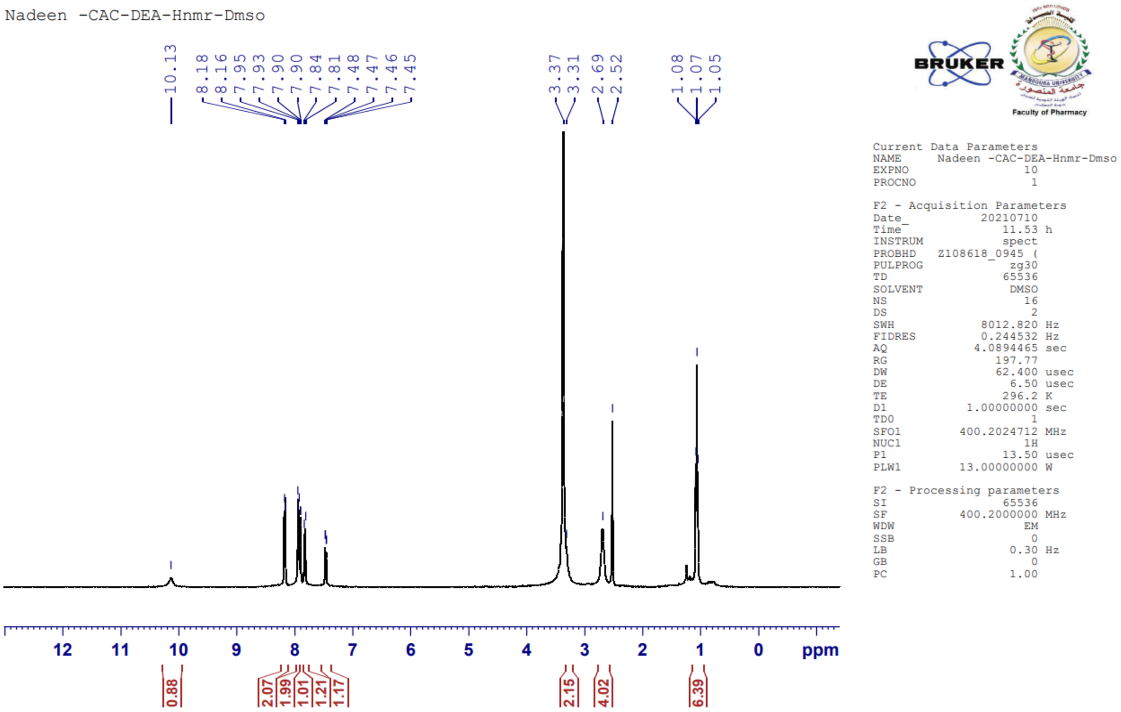

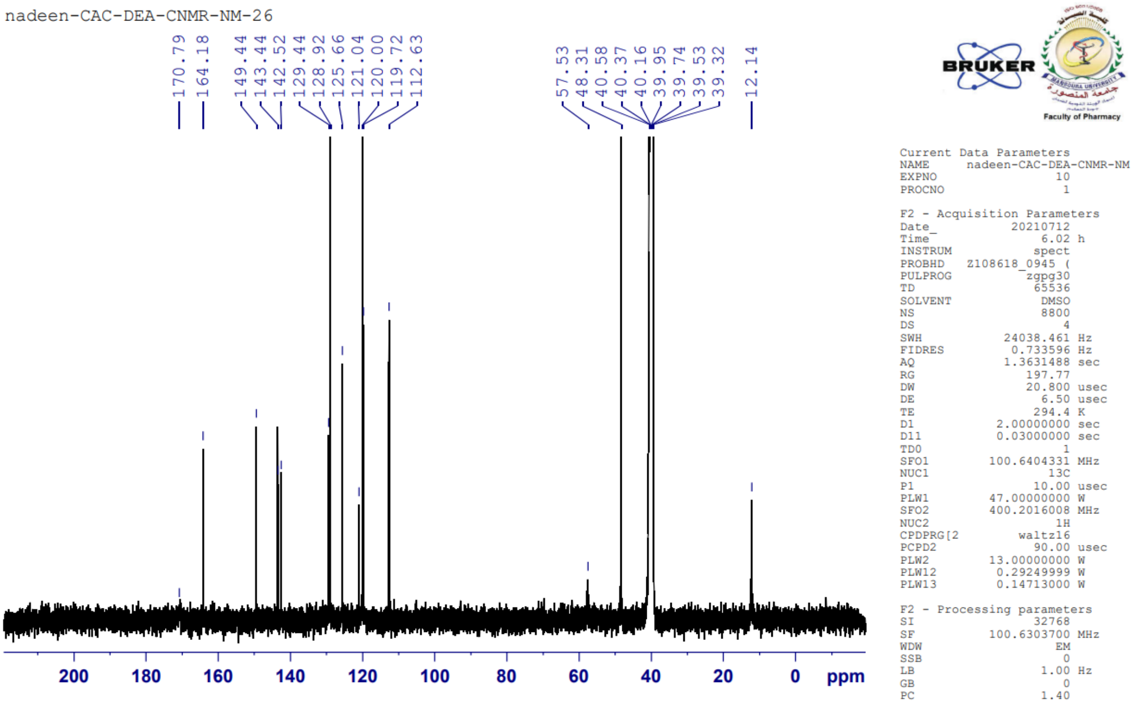

^{1}H NMR and ^{13}C NMR spectra of compound **27**

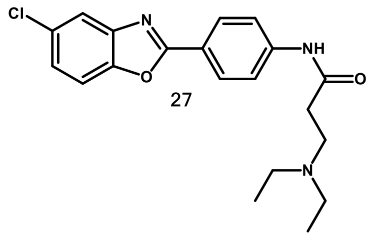

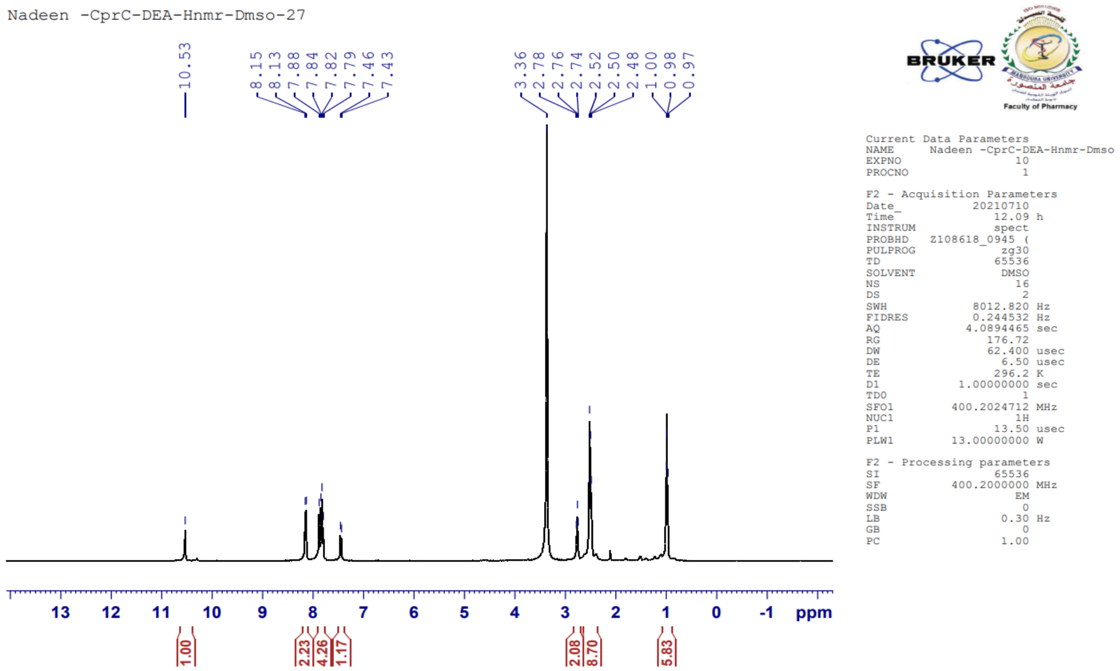

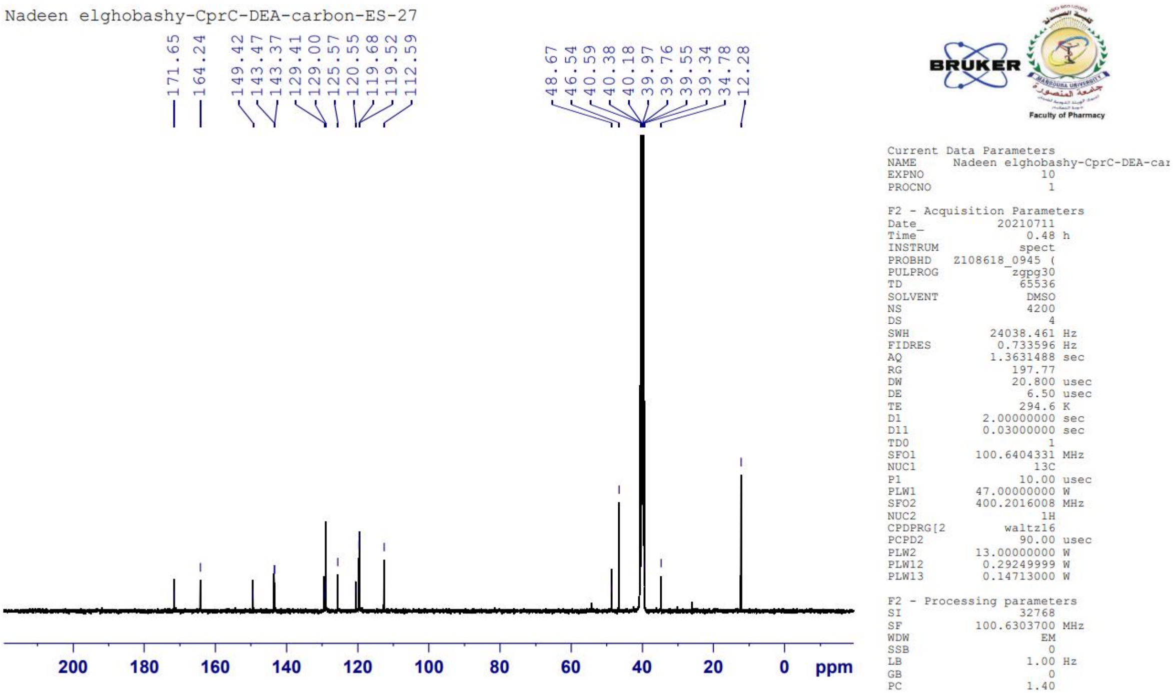

^{1}H NMR and IR spectra of compound **28**

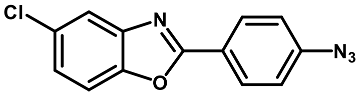

28

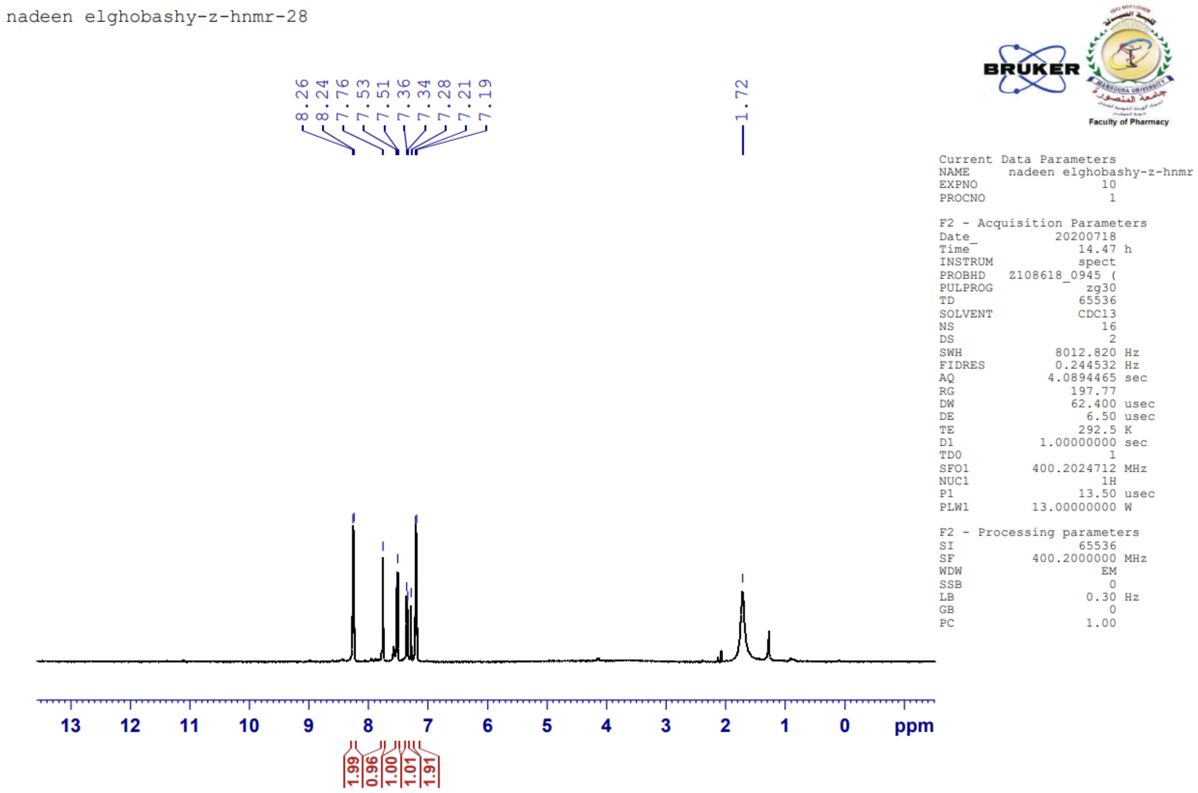

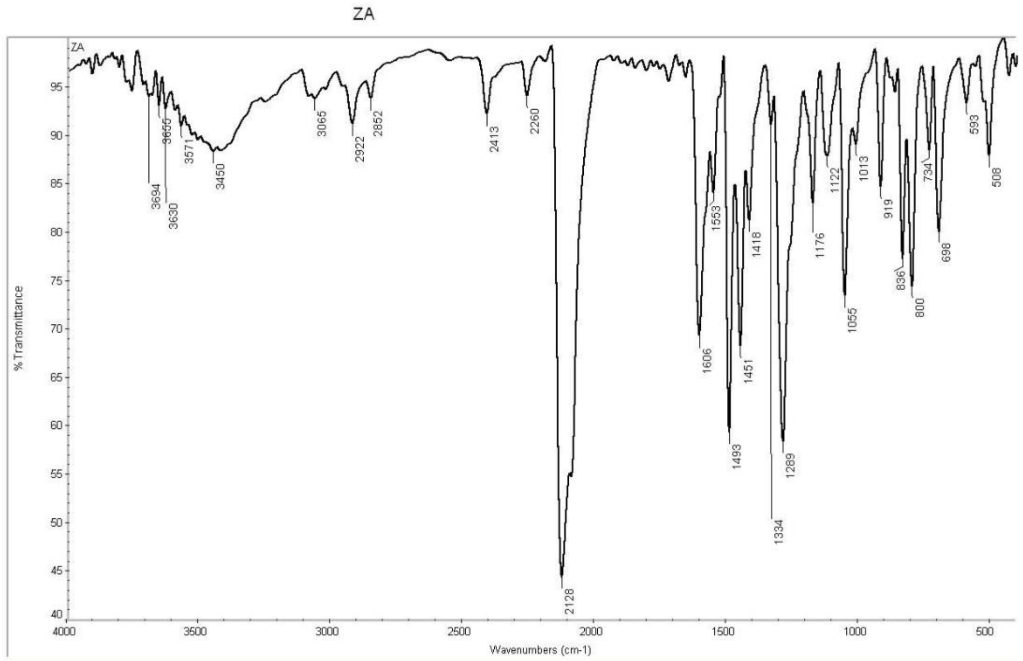

^{1}H NMR, ^{13}C NMR and IR spectra of compound **29**

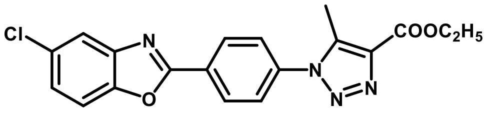

29

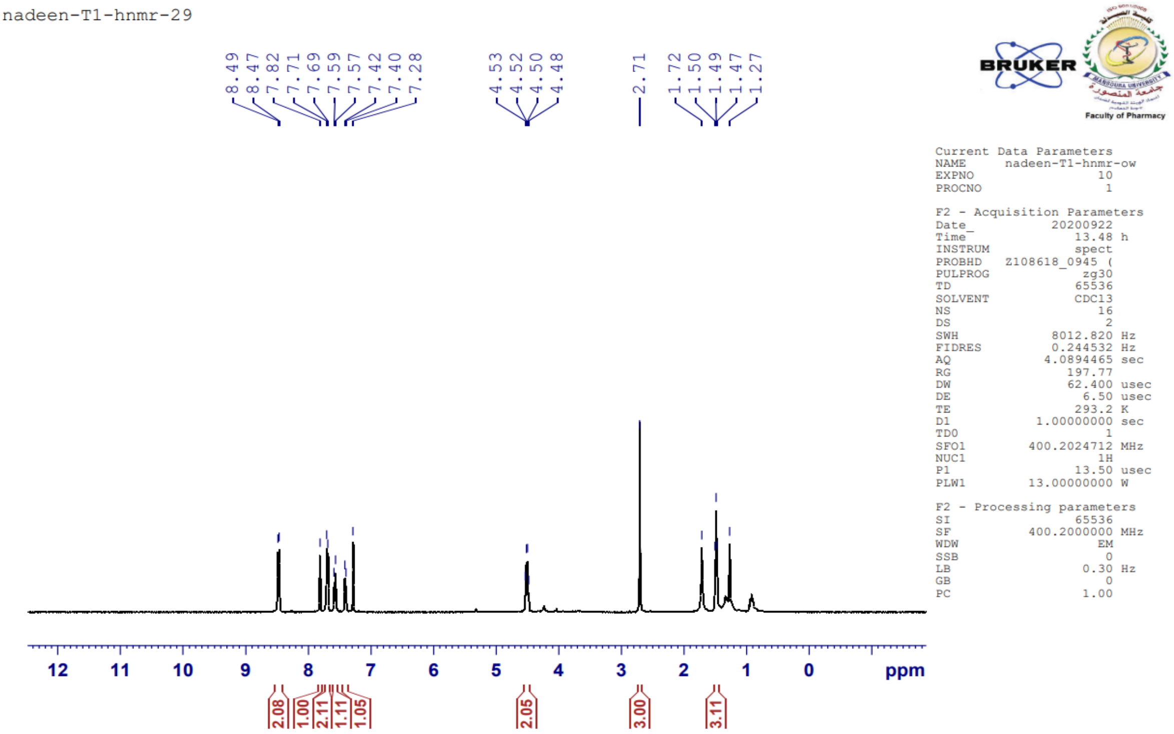

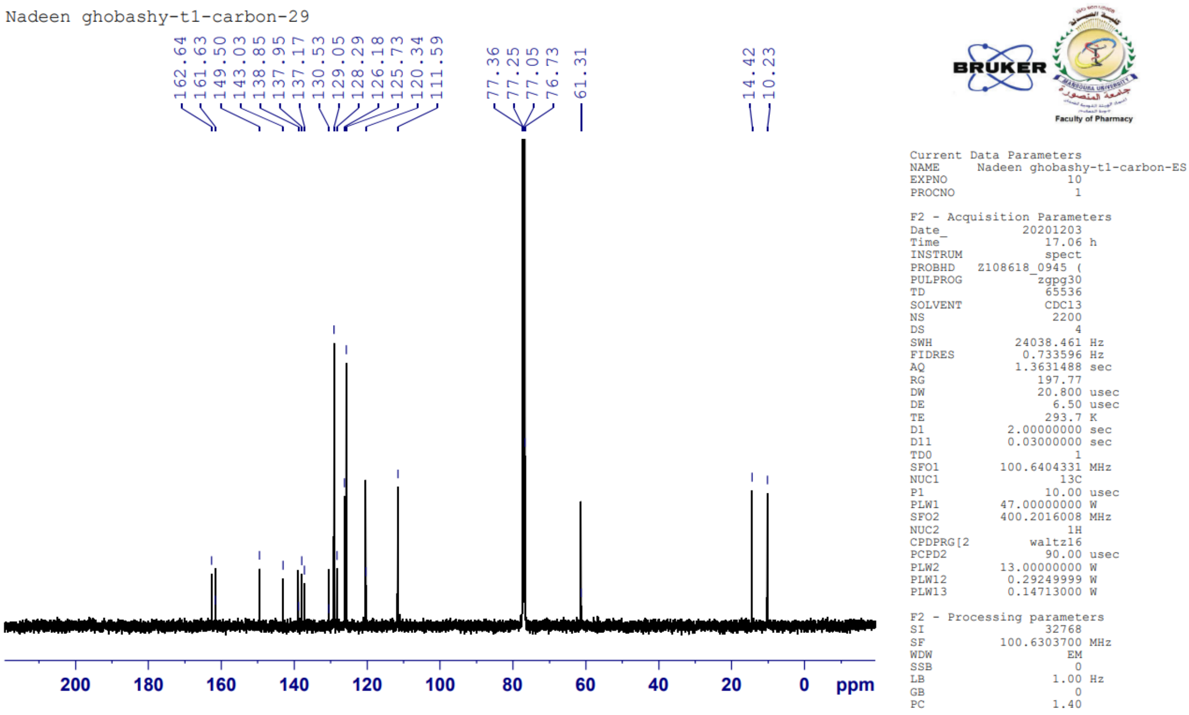

T1

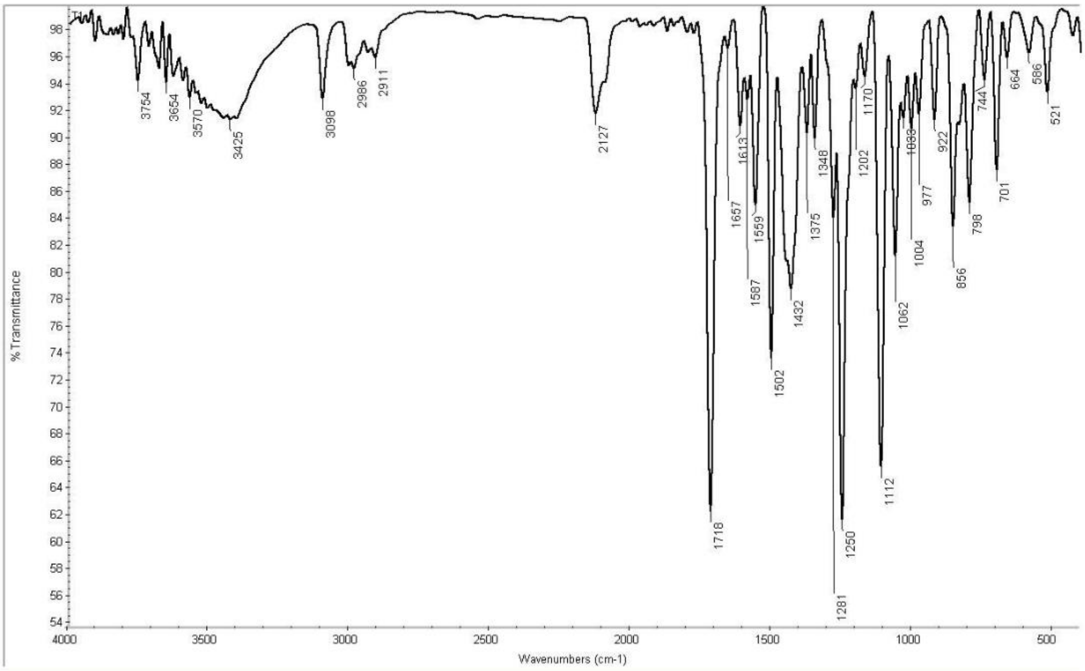
% Transmittance
Wavenumbers (cm-1)
3754
3654
3570
3425
3098
2986
2911
2127
1718
1657
1613
1587
1559
1502
1432
1375
1348
1281
1250
1202
1170
1112
1083
1062
1004
977
922
856
798
744
701
664
586
521

^{1}H NMR , ^{13}C NMR and IR spectra of compound **30**

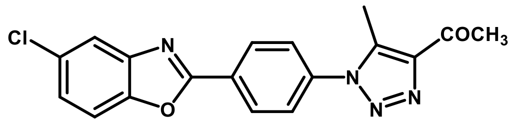

30

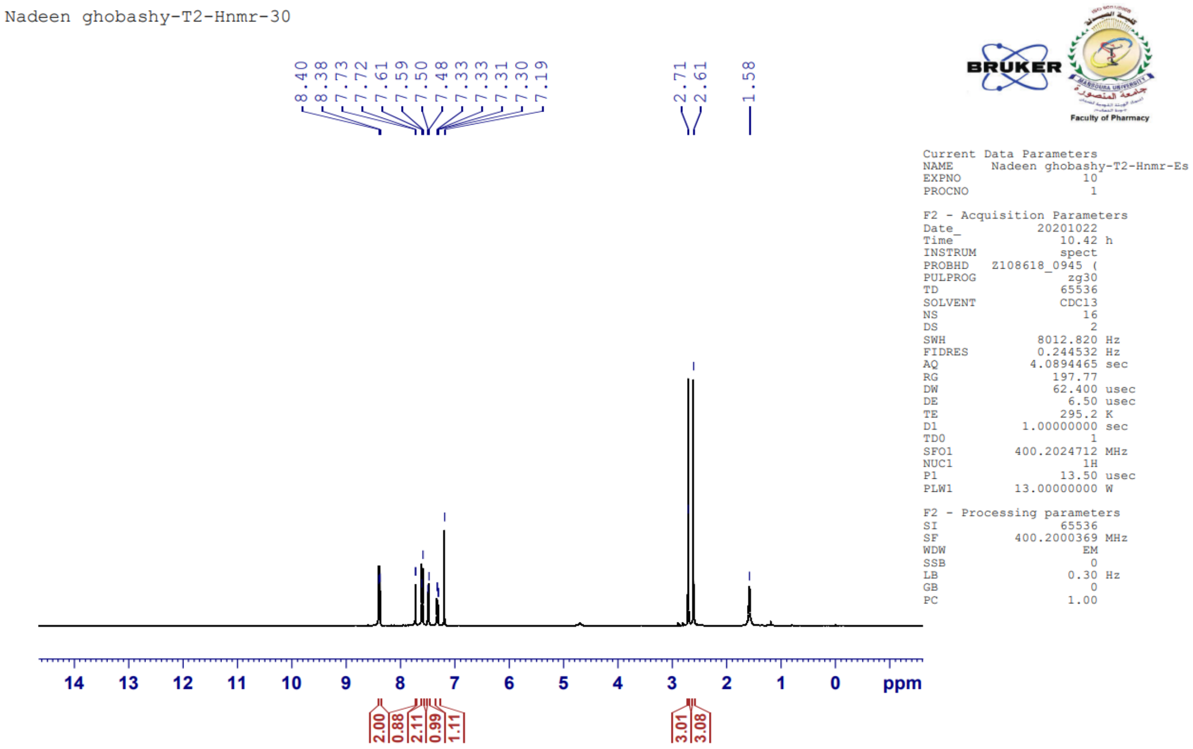

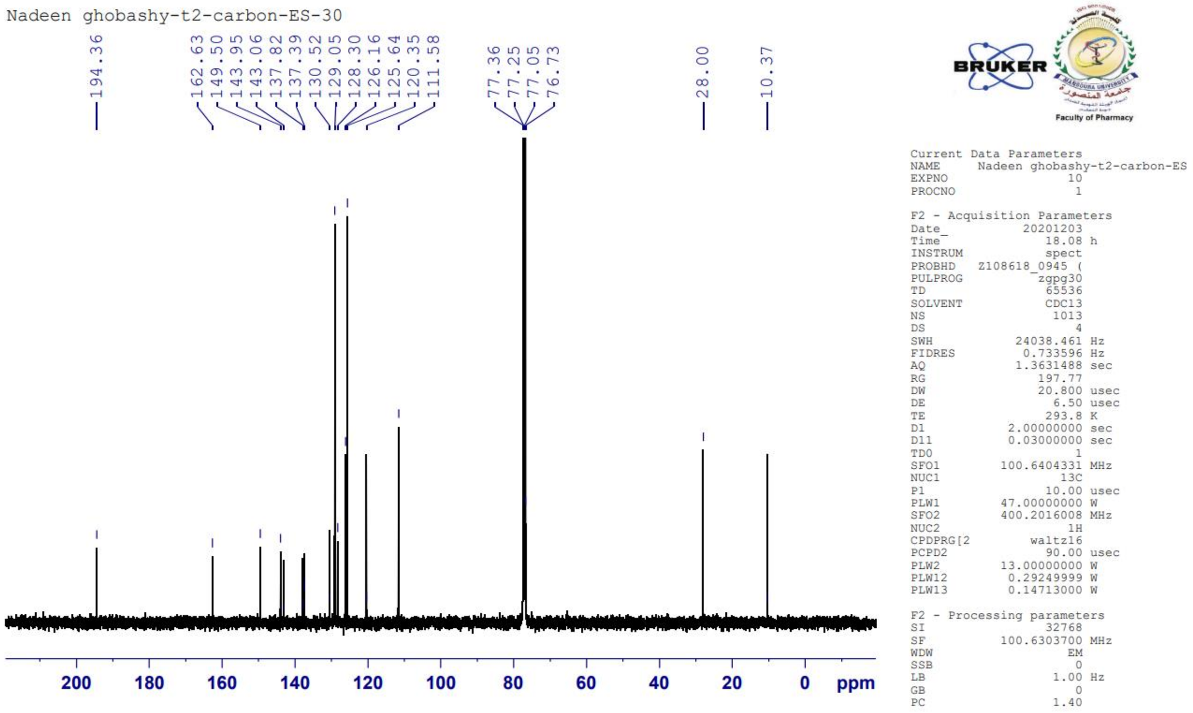

T2

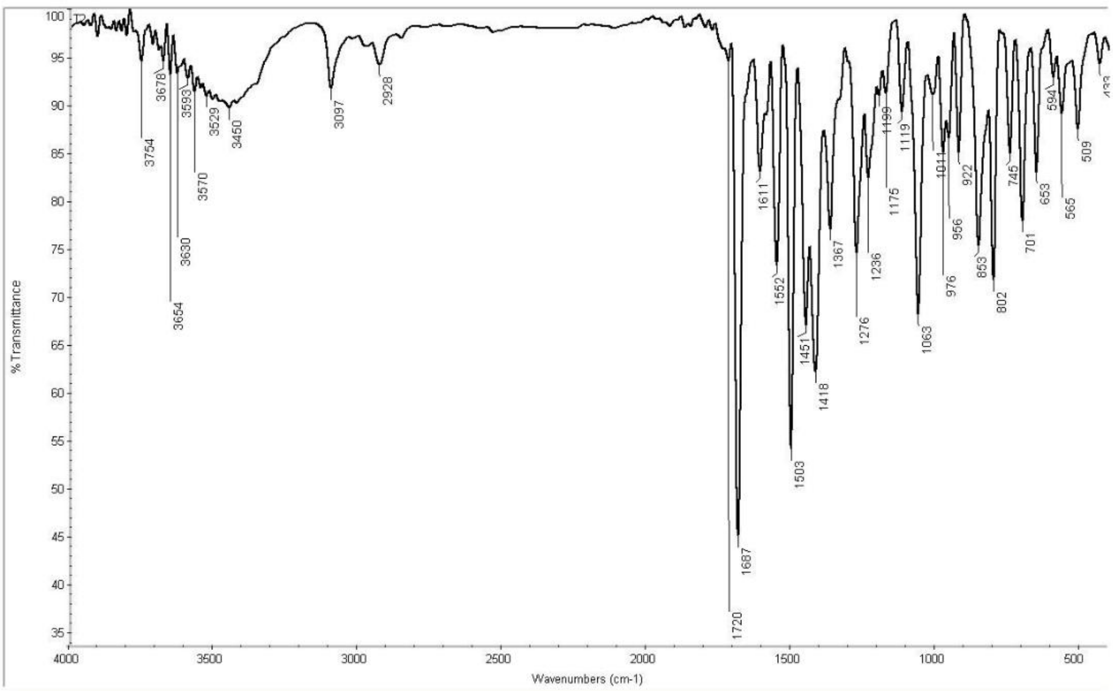
% Transmittance
Wavenumbers (cm-1)
100
95
90
85
80
75
70
65
60
55
50
45
40
35
4000
3500
3000
2500
2000
1500
1000
500
3754
3678
3654
3630
3593
3570
3529
3450
3097
2928
1720
1687
1611
1552
1503
1451
1418
1367
1276
1236
1199
1175
1119
1063
1011
976
956
922
853
802
745
701
653
594
565
509

Suppl. S2. *In vitro* PARP-2 enzyme inhibition assay

Researcher : Dr. Nadeen Ghobashy email:elghobashynadeen@gmail.com mob.01008873340

Assay : PARP-2

Samples : 09 Samples

cell line : ---

Ref. : ---

Date : 29-09-2021

Reader : ROBONIK P2000 Eliza Reader wl 450 nm

Kit used : ---

Solvent : DMSO

Assay samples : ---

Table S1. PARP-2 enzyme inhibition results (IC_{50} µM) of compounds **11-14, 21, 22, 25-27** and olaparib

Lab Report

Ser	sample	PARP-2	SD
	code	IC50	±
		uM	
1	s11	0.19	0.01
2	s12	0.07	0.004
3	s13	0.106	0.006
4	s14	0.084	0.005
5	s21	0.406	0.022
6	s22	0.267	0.015
7	s25	0.074	0.004
8	s26	0.292	0.016
9	s27	0.057	0.003
***	Olaparib	0.02	0.002

Table S2. Raw data of *in vitro* PARP-2 enzyme inhibition assay

Detailed results

PARP2												
code	IC50	conc	log	%inh	T2	T1	ΔT	RFU2	RFU1	ΔRFU	slope	K.Activity
s11		10	1	84.97	30	0	30	15.03	0	15.03	3.3333	18.0362
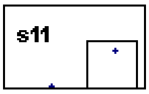		1	0	58.88	30	0	30	41.12	0	41.12	3.3333	49.3445
		0.1	-1	43.61	30	0	30	56.39	0	56.39	3.3333	67.6687
		0.01	-2	28.75	30	0	30	71.25	0	71.25	3.3333	85.5009
EC				0	30	0	30	100	0	100	3.3333	120

code	IC50	conc.ng/ml	log conc	%inh	T2	T1	ΔT	RFU2	RFU1	ΔRFU	slope	K.Activity
s12		10	1	90.18	30	0	30	9.82	0	9.82	3.3333	11.7841
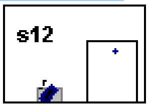		1	0	67.93	30	0	30	32.07	0	32.07	3.3333	38.4844
		0.1	-1	53.11	30	0	30	46.89	0	46.89	3.3333	56.2686
		0.01	-2	35.69	30	0	30	64.31	0	64.31	3.3333	77.1728
EC				0	30	0	30	100	0	100	3.3333	120

code	IC50	conc.ng/ml	log conc	%inh	T2	T1	ΔT	RFU2	RFU1	ΔRFU	slope	K.Activity
s13		10	1	88.69	30	0	30	11.31	0	11.31	3.3333	13.5721
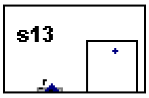		1	0	71.31	30	0	30	28.69	0	28.69	3.3333	34.4283
		0.1	-1	46.82	30	0	30	53.18	0	53.18	3.3333	63.8166
		0.01	-2	30.58	30	0	30	69.42	0	69.42	3.3333	83.3048
EC				0	30	0	30	100	0	100	3.3333	120

code	IC50	conc.ng/ml	log conc	%inh	T2	T1	ΔT	RFU2	RFU1	ΔRFU	slope	K.Activity
s14		10	1	90.54	30	0	30	9.46	0	9.46	3.3333	11.3521
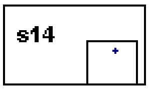		1	0	75.72	30	0	30	24.28	0	24.28	3.3333	29.1363
		0.1	-1	46.83	30	0	30	53.17	0	53.17	3.3333	63.8046
		0.01	-2	33.28	30	0	30	66.72	0	66.72	3.3333	80.0648

EC				0	30	0	30	100	0	100	3.3333	120

code	IC50	conc.ng/ml	log conc	%inh	T2	T1	ΔT	RFU2	RFU1	ΔRFU	slope	K.Activity
**s21**		10	1	81.94	30	0	30	18.06	0	18.06	3.3333	21.6722
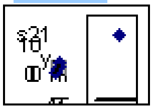		1	0	52.41	30	0	30	47.59	0	47.59	3.3333	57.1086
		0.1	-1	36.15	30	0	30	63.85	0	63.85	3.3333	76.6208
		0.01	-2	20.88	30	0	30	79.12	0	79.12	3.3333	94.9449
EC				0	30	0	30	100	0	100	3.3333	120

code	IC50	conc.ng/ml	log conc	%inh	T2	T1	ΔT	RFU2	RFU1	ΔRFU	slope	K.Activity
**s22**		10	1	86.49	30	0	30	13.51	0	13.51	3.3333	16.2122
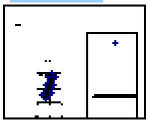		1	0	58.97	30	0	30	41.03	0	41.03	3.3333	49.2365
		0.1	-1	33.43	30	0	30	66.57	0	66.57	3.3333	79.8848
		0.01	-2	27.08	30	0	30	72.92	0	72.92	3.3333	87.5049
EC				0	30	0	30	100	0	100	3.3333	120

code	IC50	conc.ng/ml	log conc	%inh	T2	T1	ΔT	RFU2	RFU1	ΔRFU	slope	K.Activity
**s25**		10	1	88.59	30	0	30	11.41	0	11.41	3.3333	13.6921
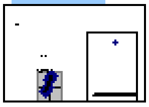		1	0	73.45	30	0	30	26.55	0	26.55	3.3333	31.8603
		0.1	-1	47.92	30	0	30	52.08	0	52.08	3.3333	62.4966
		0.01	-2	36.11	30	0	30	63.89	0	63.89	3.3333	76.6688
EC				0	30	0	30	100	0	100	3.3333	120

code	IC50	conc.ng/ml	log conc	%inh	T2	T1	ΔT	RFU2	RFU1	ΔRFU	slope	K.Activity
**s26**		10	1	86.92	30	0	30	13.08	0	13.08	3.3333	15.6962
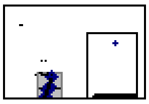		1	0	55.9	30	0	30	44.1	0	44.1	3.3333	52.9205
		0.1	-1	38.75	30	0	30	61.25	0	61.25	3.3333	73.5007
		0.01	-2	21.36	30	0	30	78.64	0	78.64	3.3333	94.3689
EC				0	30	0	30	100	0	100	3.3333	120

code	IC50	conc.ng/ml	log conc	%inh	T2	T1	ΔT	RFU2	RFU1	ΔRFU	slope	K.Activity
**s27**		10	1	88.84	30	0	30	11.16	0	11.16	3.3333	13.3921
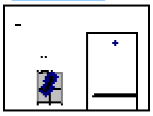		1	0	73.48	30	0	30	26.52	0	26.52	3.3333	31.8243
		0.1	-1	52.67	30	0	30	47.33	0	47.33	3.3333	56.7966
		0.01	-2	37.25	30	0	30	62.75	0	62.75	3.3333	75.3008
EC				0	30	0	30	100	0	100	3.3333	120

code	IC50	conc.ng/ml	log conc	%inh	T2	T1	ΔT	RFU2	RFU1	ΔRFU	slope	K.Activity
**Olaparib**		10	1	91.45	30	0	30	8.55	0	8.55	3.3333	10.2601
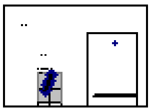		1	0	76.59	30	0	30	23.41	0	23.41	3.3333	28.0923
		0.1	-1	60.14	30	0	30	39.86	0	39.86	3.3333	47.8325
		0.01	-2	41.09	30	0	30	58.91	0	58.91	3.3333	70.6927
EC				0	30	0	30	100	0	100	3.3333	120

Fig. S1. Calculations of % inhibition of compounds**11-14, 21, 22**, **25-27** and olaparib

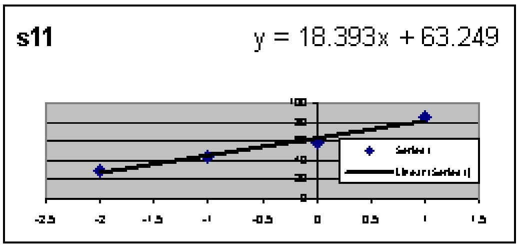

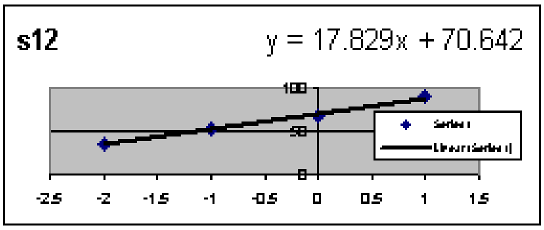

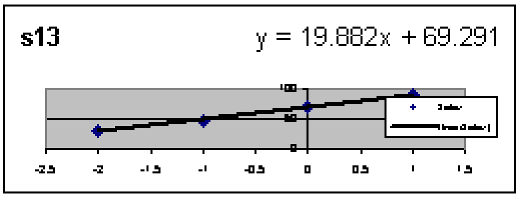

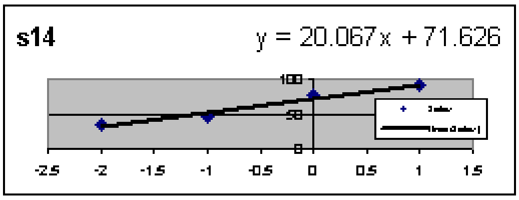

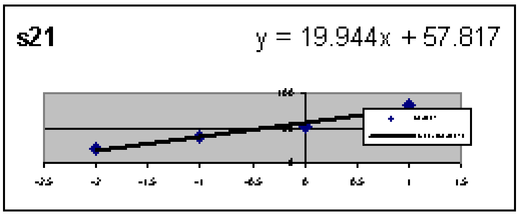

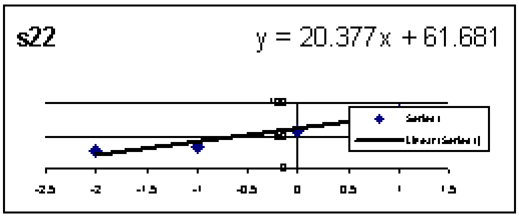

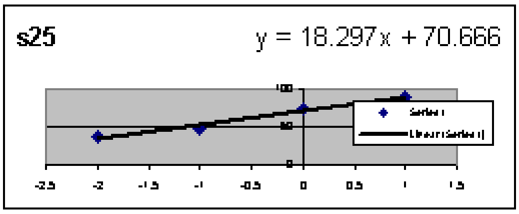

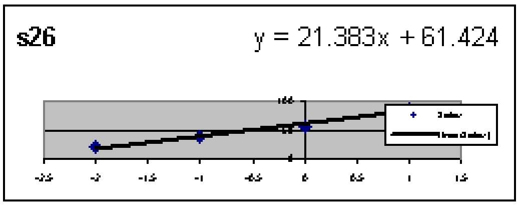

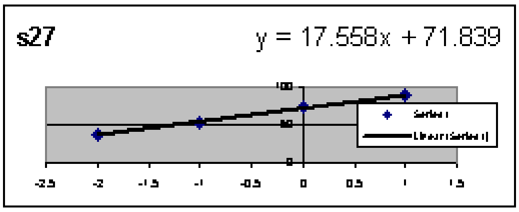

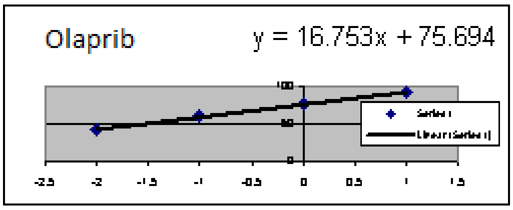

Suppl. S3. Cell cycle analysis for compounds **11**, **12**, **13** and **27**

**Researcher**	: Dr.Nadeen Ghobashy email : elghobashynadeen@gmail.com mob. 01008873340
Assay	: Cell Cycle Analysis
Samples	: 04 samples
cell line	: ---
Ref.	: ---
Date	: 24-10-2021
Reader	: BD FACSCalibur
Kit used	: ab139418_Propidium Iodide Flow Cytometry Kit/BD
Solvent	: DMSO
Assay samples	: Cell culture

Table S3. Cell cycle phase distribution in in MCF-7 cell line treated with vehicle control and the newly synthesised compounds; **11**, **12**, **13** and **27**.

Lab Report

ser	Sample data		Results DNA content				
	**code**	**IC50 uM**	**%G0-G1**	**%S**	**%G2/M**	**%Pre-G1**	**Comment**
1	**11/MCF7**	3	68.02	24.76	7.22	45.07	cell growth arrest@ G1
2	**12/MCF7**	6	41.78	26.18	32.04	33.47	cell growth arrest@ G2/M
3	**13/MCF7**	8	44.98	28.51	26.51	29.66	cell growth arrest@ G2/M
4	**27/MCF7**	16	62.38	33.16	4.46	25.91	cell growth arrest@ G1/S
5	**cont.MCF7**	---	58.21	29.95	11.84	1.85	---

Fig. S2. Cell cycle phase distribution in in MCF-7 cell line treated with vehicle control and the newly synthesised compounds; **11**, **12**, **13** and **27**.

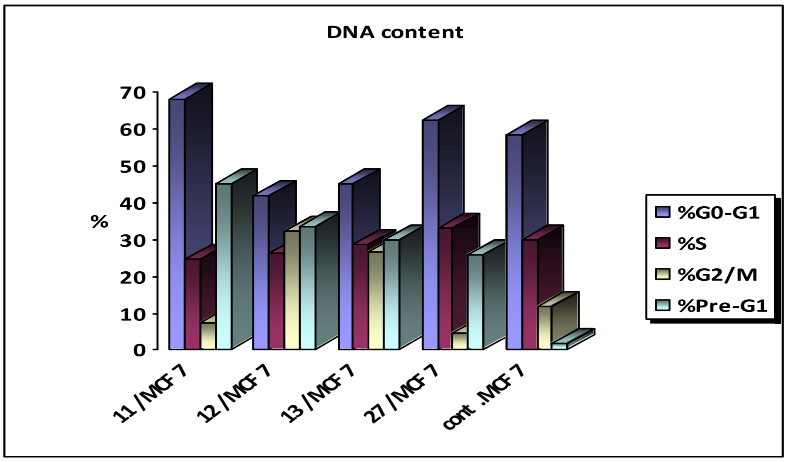

Suppl. S4. Detection of apoptosis and necrosis for compounds **11**, **12**, **13** and **27**

Table S4. Apoptosis percentage in MCF-7 cells treated with vehicle control and the newly synthesised compounds; **11, 12, 13** and **27**

s	code	Apoptosis			Necrosis
		Total	Early	Late	
1	**11/MCF7**	45.07	16.31	23.02	5.74
2	**12/MCF7**	33.47	22.52	3.72	7.23
3	**13/MCF7**	29.66	12.28	10.99	6.39
4	**27/MCF7**	25.91	19.26	4.58	2.07
5	**cont.MCF7**	1.85	0.37	0.33	1.15

Fig. S3. Apoptosis percentage in MCF-7 cells treated with vehicle control and the newly synthesised compounds; **11, 12, 13** and **27**

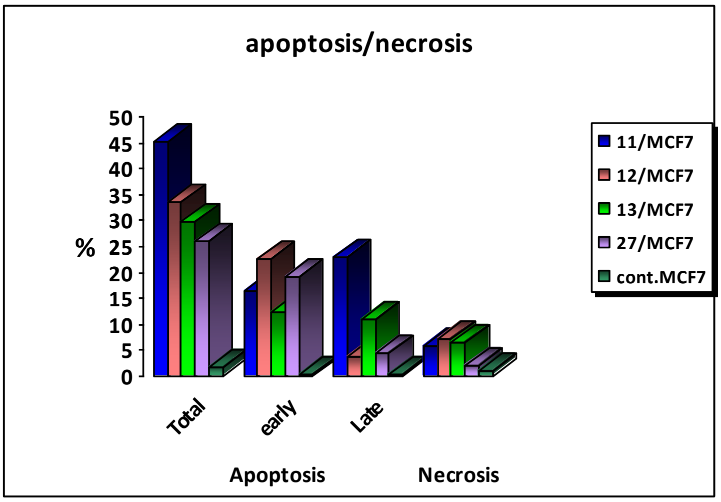

Supplement: Supplementary file 1 — Supplementary Information. [file 41598_2022_20260_MOESM1_ESM.pdf]
